# Species distribution models of European Turtle Doves in Germany are more reliable with presence only rather than presence absence data

**DOI:** 10.1038/s41598-018-35318-2

**Published:** 2018-11-15

**Authors:** Melanie Marx, Petra Quillfeldt

**Affiliations:** Department of Animal Ecology & Systematics, Justus-Liebig-University Giessen, Heinrich- Buff-Ring 38, 35392 Giessen, Germany

## Abstract

Species distribution models (SDMs) can help to describe potential occurrence areas and habitat requirements of a species. These data represent key information in ecology and conservation, particularly for rare or endangered species. Presence absence (PA) and presence only (PO) records of European Turtle Doves *Streptopelia turtur* in Germany were used to run SDMs, whilst climate and land coverage variables provided environmental information. GLM (Generalised Linear model), GBM (Generalised Boosted model), CTA (Classification Tree analysis), SRE (Surface Range Envelope) and RF (Random Forests) algorithms were run with both datasets. Best model quality was obtained with PO in the RF algorithm (AUC 0.83). PA and PO probability maps differed substantially, but both excluded mountainous regions as potential occurrence areas. However, PO probability maps were more discriminatory and highlighted a possible distribution of Turtle Doves near Saarbrucken, west of Dusseldorf, in the Black Forest lowlands and Lusatia. Mainly, the climate variables ‘minimum temperature in January’ and ‘precipitation of the warmest quarter’ shaped these results, but variables like soil type or agricultural management strategy could improve future SDMs to specify local habitat requirements and develop habitat management strategies. Eventually, the study demonstrated the utility of PO data in SDMs, particularly for scarce species.

## Introduction

Knowledge about species distributions and their habitat requirements is a key subject in ecology and conservation. Therefore, habitat suitability models or species distribution models (SDMs) have been used to describe species habitats or presence probabilities^1^. Many SDMs address questions about future predictions for species distributions regarding climate or land coverage changes^2,3^. Others study current distributions and the characteristics of the occupied habitats^4–6^, which provides important information for conservation management of detected key habitats^7^, such as assignment of nature reserves or special protection areas to provide localities where endangered or vulnerable species might be able to persist^8^.

To analyse those questions, different databases with spatial information about species distributions can be used. Possible sources include museum collections^2,9,10^, atlas distributions^11^, data from field surveys^12^, and also citizen-based species records collected via online platforms^13–16^.

To model species distributions, biotic and abiotic parameters can be included in the analysis: a.) Environmental parameters, i.e. inter- and intraspecific interactions, climate, land cover or topography and b.) Spatial records of the study species^5,17^. Abiotic parameters are often digitally available in large databases (e.g. climate data from www.worldclim.org)^18^. Considering species records, those can be available as presence absence (PA) or presence only (PO) data. PA datasets contain notifications of presence (P) and absence (A) of certain species in surveyed study sites. Absence data arise for a species when it was not detected during standardised field observations and therefore, did not occur in the study area. PO datasets provide only presence records of the target species and can be used to model the distribution of a species, but one needs to consider biases caused by observers (detection bias, recording or reporting bias, geographic bias), due to non-standardised sampling methods^16,19^.

Different modelling algorithms can be used in SDMs. Generalised Linear Models (GLM) or Generalised Additive Models (GAM) have been applied frequently to PA data to generate suitability models^20–22^. Other commonly applied model algorithms for PA datasets were e.g. Classification and Regression Tree analysis (CART^23–25^), Artificial Neural Networks (ANN^23^) and Multivariate Adaptive Regression Splines (MARS^23,26^). For PO data Ecological Niche Factor Analysis (ENFA^27,28^) and Maximum Entropy Method (MAXENT^29^) have been widely used, because these algorithms do not require absence data.

Generally, it is recommended to use PA data^1^, if available. However, some studies have shown that modelling approaches applied to PO datasets with ENFA or MAXENT provide equal or slightly better SDM performance than models conducted with PA data^5,12,27,30^. Also, the creation of simulated pseudo-absence points in PO datasets is possible and allows application of modelling algorithms usually used on PA data. These pseudo-absences may improve the model quality of the applied algorithm, because they can include background information about non-occupied environments^31,32^. Nevertheless, their application should be carefully assessed, because they might be biased – e.g. when a species is widespread or presence data is rare^31^. Thus, false pseudo-absences may be modelled into a possibly suitable area without species occurrence records, due to the scarcity of the species. However, in PA datasets false absence can also be generated, if a species was not detected during field work and therefore, was noted as absent although it inhabited the survey area^28^.

Due to the strong decline of more than 78% from the 1980s until present, the European Turtle Dove (*Streptopelia turtur*, hereafter referred to as Turtle Dove) is listed as a vulnerable species^33,34^. A simulation study from the UK, found an annual population decline of 17.5% due to a decreased number of fledglings^35^. The low breeding success in the UK has been linked to agricultural intensification and an intensified use of herbicides, which led to habitat loss and changes in food availability and quality on breeding grounds, not only in the UK but also in other European countries such as Germany^35–39^. To halt these severe population declines, it is important to discover key breeding habitats for Turtle Doves and to develop management plans for those areas.

In our study, two different datasets with Turtle Dove records from Germany were available – PA data from the ‘Monitoring of breeding birds’ scheme collected by the DDA (Dachverband Deutscher Avifaunisten e.V.) and citizen-based PO data from the online platform www.ornitho.de^40^ (ornitho-data), which is used to record bird sightings. On the website observers can input their bird data as sighting records, but there is no regulation to report every sighted species or to report species using a standardised field protocol^16^. Although observers can add checklists (standardised method) with records about observation time and date as well as presence and absence of certain species (see www.ornitho.de)^40^, it seems that these checklists are not commonly used (own observation, judging by the indications of the daily summaries in different regions), but instead non-standardised bird sightings are submitted. Also, there likely is a reporting bias caused by observers, with differing species identification skills, or easily detected birds are reported more frequently^13^.

In central Germany, one habitat suitability study was performed in the Wetterau, a small region of Hesse, where Turtle Doves were once known as common breeding birds. In 2012, the study again monitored all habitats known to be occupied in 1998/1999 and recorded presences and absences of Turtle Doves. Results indicated a decrease of breeding pairs by 50% in 2012 compared to 1998/99^6^. The ‘Monitoring of breeding birds’ scheme confirmed this decline in Germany, highlighting a loss of almost 33% of breeding pairs compared to the mid 1990s^41^.

Besides the recorded decline of breeding pairs, a strong positive effect of woodland and grassland was found when the effects of different environmental parameters were examined^6^. The study distinguished positive parameters for feeding and breeding habitat and revealed that dense deciduous forests and middle aged mixed forests were the most important parameters for the breeding areas. Regarding the feeding locations, the most positive parameters were grassland and forest glades.

However, it has been shown in other studies that the results of habitat models or evaluation of habitat preferences may come to other conclusions when processed on different scales^42,43^. For instance, in Great Britain (Ixworth Thorpe and Deeping St. Nicholas) habitat requirements for radio-tagged Turtle Doves were evaluated at two different scales^42^. A small scale assessment was conducted based on recorded positions of radio-tagged Turtle Doves, which were used to define home ranges. A larger scale assessment was based on a 50 m buffer around recorded locations^42^. Results showed a positive effect of pasture and a weaker effect of woodland for breeding grounds on the small scale, but was reversed when evaluated for the larger scale, with woodland as important factor and pasture with weaker effect on Turtle Dove occurrence^42^. However, the scale used depends on the aim of a study^43^. In the present study, German-wide key breeding sites and their environmental characteristics will be evaluated, which is why SDM will be conducted on a large country scale.

In our study, we aim to:

1. Compare the results of different model algorithms using first, a PA dataset and second, a PO dataset with introduced pseudo-absences
2. Compare the results of the present study to previous ones for the Wetterau in Hesse^6^ and other study sites in Europe^42^.

## Results

### PO and PA data

**Figure 1:**
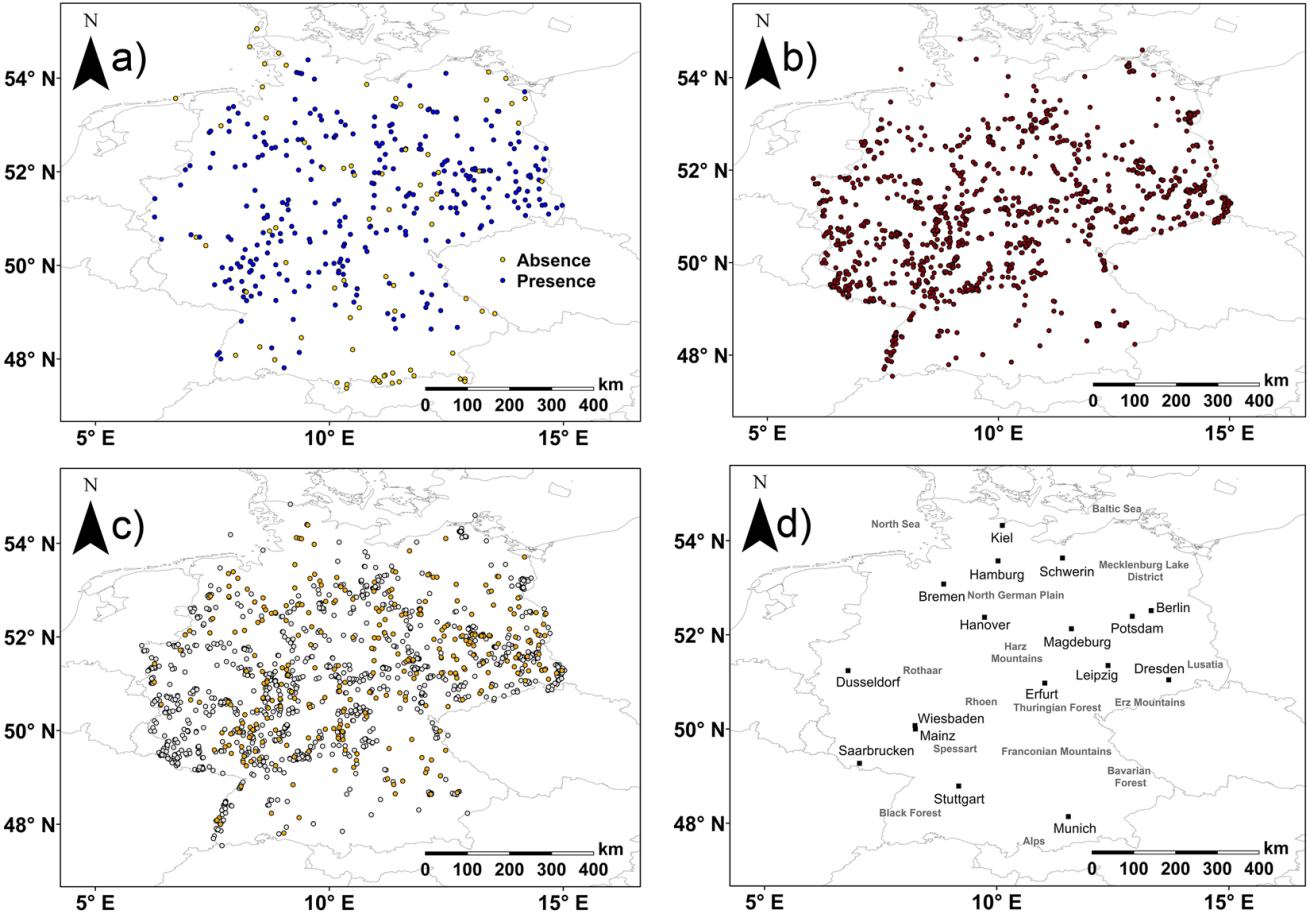
Overview maps. They show the distribution of species records of Turtle Doves for (**a**) PA (presence in blue and absence in yellow) and (**b**) PO (presence records are shown in dark red) data in Germany. (**c**) shows the positions of presence points recorded in PA and PO datasets. Presence points from PO data are given as light grey circles and the ones from PA data as yellow circles. Furthermore, (**d**) a map with Germany’s larger cities and landscapes for orientation in space is drawn according to https://www.diercke.de/content/deutschland-physische-karte-978-3-14-100800-5-19-2-190.

The model algorithms were run with two different species datasets. Both datasets were distributed across Germany, but only the PA dataset consisted of fixed study sites, which should be checked annually by volunteers. The presence points of PA and PO data showed overlaps in some regions, e.g. Potsdam, Wiesbaden and Mainz (Fig. 1). Nonetheless, there were more Turtle Dove presence points registered in the PO dataset (1168 presence points) than in the PA dataset (293 presence points). Furthermore, PO data were also registered in regions like Lusatia at the border to Poland and in western Germany at the border to France in the Black Forest or close to Saarbrucken. In these regions, the PA dataset had only a few or no registered Turtle dove presence points (Fig. 1).

### Model performance

Model performances for PA and PO data, judging by AUC value (Area under Receiver Operating Characteristic (ROC) curve^44^), sensitivity (percentage of presences correctly predicted) and specificity (percentage of absences or pseudo-absences correctly predicted), were similar within same model algorithms. Classification of model quality includes evaluation of all three values. AUC values between 0.5–0.6 describe models that failed, 0.6–0.7 represent poor model quality, 0.7–0.8 are models with fair performance and 0.8–0.9 describe a good model quality^44,45^. Here, models were of higher quality when sensitivity and specificity were similar. Overall, RF with PO data was of best quality (Table 1). The hierarchical model ranking from best to worse was identical between the PA and PO datasets and best quality was achieved with RF, followed by GBM, GLM, CTA and SRE (Table 1). Models showed better sensitivity with PA data, except SRE, but better specificity with PO data, except for SRE and GLM.

**Table 1:** Calculated AUC, sensitivity and specificity values of different SDM algorithms for European Turtle Doves.

Model	AUC PA data	Sensitivity PA data	Specificity PA data	AUC PO data	Sensitivity PO data	Specificity PO data
CTA	0.63	95.64	31.52	0.69	76.03	58.34
GBM	0.75	81.67	61.70	0.77	73.38	67.65
GLM	0.73	75.90	64.70	0.71	71.60	62.10
RF	0.76	82.35	61.96	0.83	72.34	78.07
SRE	0.60	54.65	65.91	0.56	62.97	47.54

For further description and evaluation, we only considered those model results with an AUC ≥ 0.7. Thus, we focus on RF, GBM and GLM in this study.

### Importance and influence of variables

Variable importance was evaluated for land cover and climate variables (variable names and attributes included are given in Table 2). The assessment of variable importance revealed climatic variables to be most important in the different model algorithms, particularly Bio 18 (both GBM algorithms and RF with PO data) and Bio 6 (both GLM algorithms) (Table 3). As the variable importance is computed according to Pearson’s correlation^45^, only one strong value was obtained for Bio 6 in GLM with PO data (0.84), moderate values (0.3 to 0.5) were given for Bio 18 in GBM with PO data and Bio 6 in GLM with PA data. Weak values (0.1–0.3), but of highest importance were computed for Bio 18 in GBM with PA data and RF run with PO data. Only once ‘forest’ was of highest importance in RF with PA data, but showed a weak value (0.11). Although ‘forest’ was of weak, but highest importance only in RF, it showed the second highest importance (0.20) in the GBM algorithm generated with PA data and was the third important variable in GLM run with PA data (Table 3).

**Table 2:** Variables used in SDMs for European Turtle Doves.

Variable	Includes following landscape types or climatic attributes
Wet areas	All wetlands and water bodies, including swamps and marshes
Permanent cultures	Wine, fruit orchards and berries
Forest	Deciduous forests, coniferous forests, mixed forests
Pasture	Grassland, meadows
Herbs and shrubs	Heathland, transitional woodland/shrub
No/little vegetation	Open land; e.g. beach, dunes, sandy or rocky areas, glaciers, burned regions
Urban areas	Cities, villages, industrial areas, haven, airports, dumps, excavation areas
Bio 2	Mean diurnal temperature range (Mean of daily (max temp - min temp))
Bio 6	Minimum temperature of the coldest month
Bio 7	Temperature annual range
Bio 8	Mean temperature of wettest quarter
Bio 9	Mean temperature of driest quarter
Bio 11	Mean temperature of coldest quarter
Bio 15	Precipitation seasonality
Bio 18	Precipitation of warmest quarter

**Table 3:** Variable importance for different habitat suitability models for European Turtle Doves.

Variable	GBM PA data	GBM PO data	GLM PA data	GLM PO data	RF PA data	RF PO data
Wet areas	<0.01	<0.01	0.05	0.01	<0.01	0.01
Permanent cultures	0.01	<0.01	0.02	0.01	0.01	<0.01
Forest	0.20	0.04	0.16	0.06	**0.11**	0.03
Pasture	0.01	0.02	0.14	0.01	0.02	0.05
Herbs and shrubs	<0.01	0.03	0.02	0.05	0.01	0.01
No/little vegetation	<0.01	<0.01	0.14	<0.01	0.01	<0.01
Urban areas	0.01	0.03	0.01	0.06	0.02	0.03
Bio 2	0.05	0.12	0.01	0.02	0.04	0.07
Bio 6	0.04	0.01	**0.39**	**0.84**	0.04	0.04
Bio 7	0.01	0.02	0.10	0.50	0.01	0.04
Bio 8	0.01	0.05	0.07	0.13	0.01	0.03
Bio 9	0.02	0.02	0.04	0.03	0.03	0.04
Bio 11	0.01	0.07	0.13	0.30	0.02	0.05
Bio 15	0.01	0.03	0.01	0.11	0.01	0.06
Bio 18	**0.25**	**0.34**	0.20	0.07	0.07	**0.14**

The highest variable importance value for each model is highlighted with bold and underlined numbers.

Due to the importance of Bio 6, Bio 18 and ‘forest’, their response plots were evaluated in more detail.

**Figure 2:**
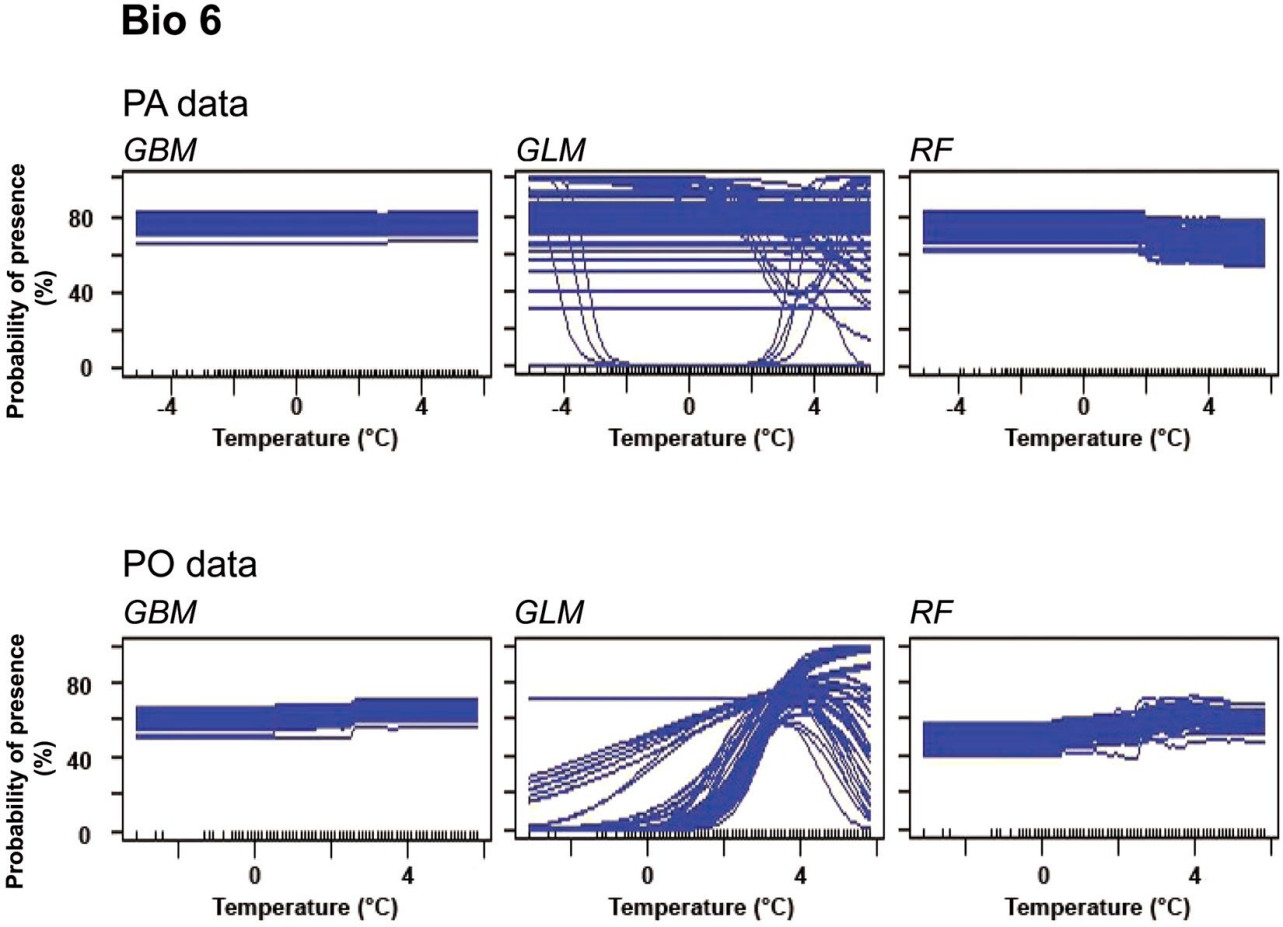
Response curves of variable Bio 6 (Minimum temperature of coldest month). The graphs were created for three species distribution models of Turtle Doves run with PA and PO data.

For Bio 6, response plots for GBMs and RFs run with PA and PO data mainly showed a constant course, but for PO data there was a slight increase of occurrence probability when the minimum temperature of the coldest month was higher than 1 °C. The response plot for GLM with PA data did not highlight any effect on the occurrence probability of Turtle Doves, but the one for PO data might show an optimum temperature of 4 °C (Fig. 2).

**Figure 3:**
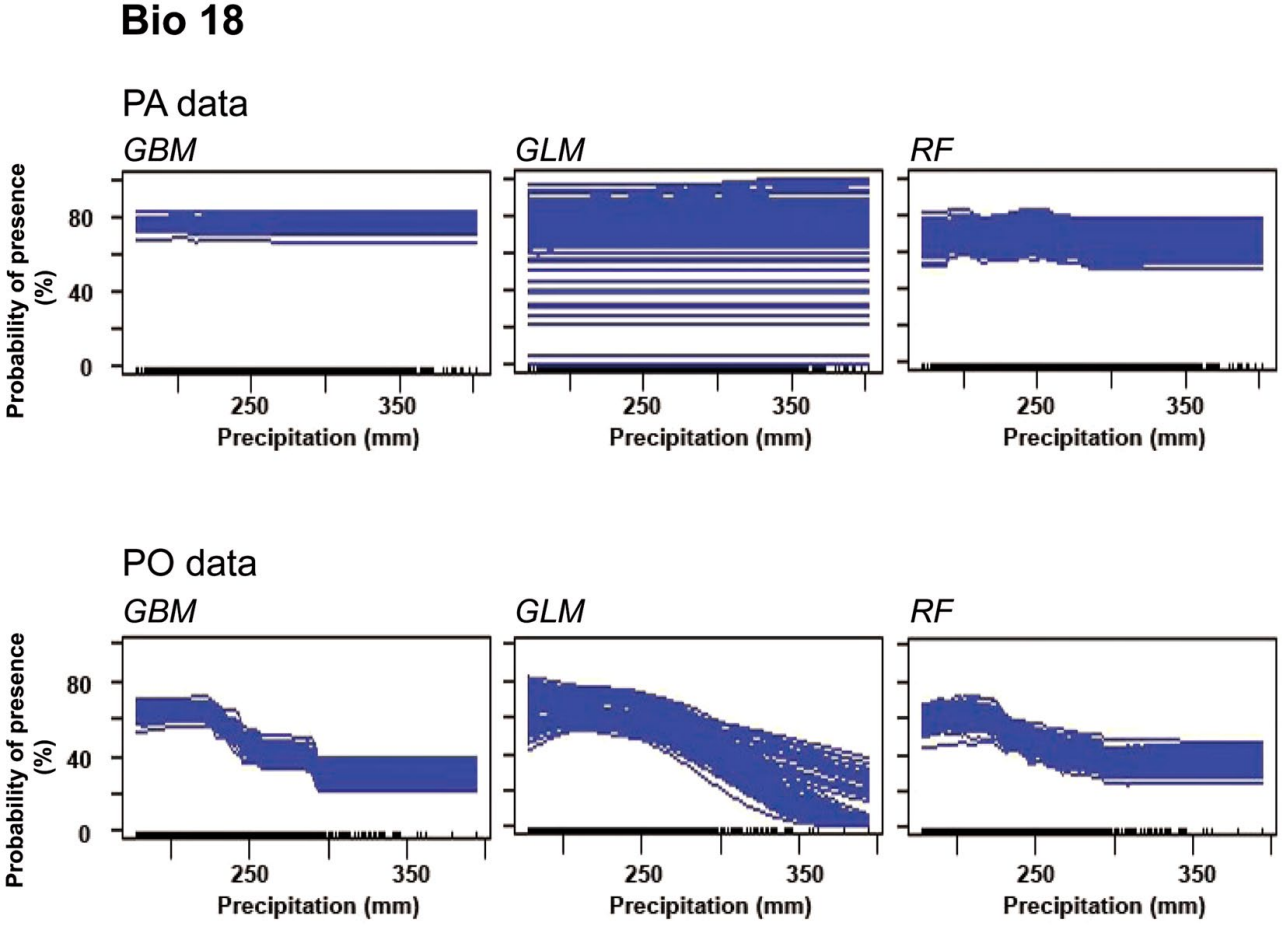
Response curves of variable Bio 18 (Precipitation of warmest quarter). The graphs were created for three species distribution models of Turtle Doves run with PA and PO data.

Regarding Bio 18, response curves of GBM and RF for PA data depicted a constant course and the GLM graph for PA did not show an impact of precipitation during the warmest quarter on Turtle Dove occurrence. For PO data, all response curves depicted a higher presence probability, when the precipitation of the warmest quarter was lower than 225 mm (Fig. 3).

**Figure 4:**
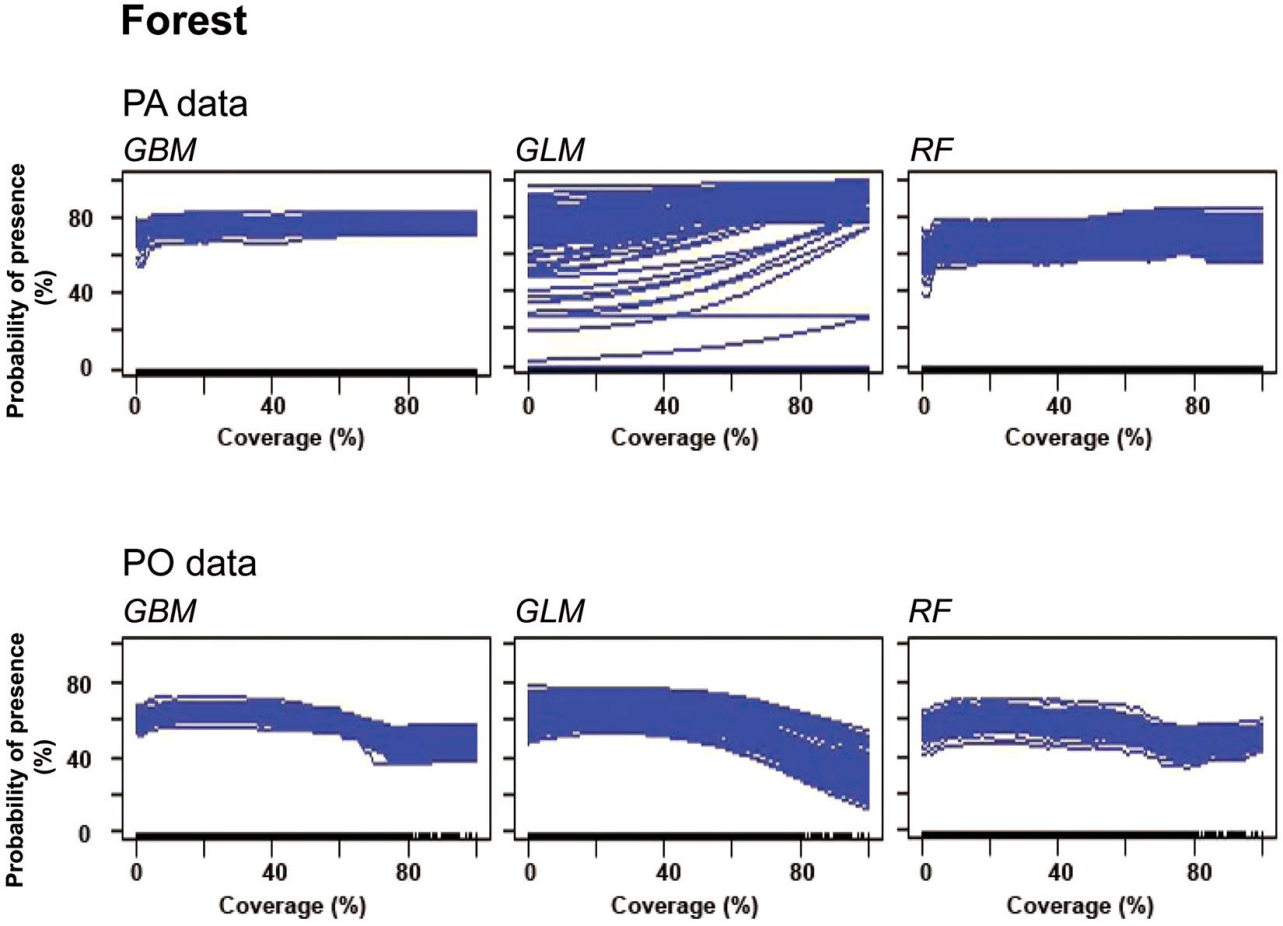
Response curves of the land coverage variable forest. The graphs were created for three species distribution models of Turtle Doves run with PA and PO data.

Furthermore,’forest’ response plots for PA data mainly showed a constant course, but GLM might depict a slight increase on the occurrence probability of Turtle Doves when forest coverage was higher than 40%. Response plots for GBM, GLM and RF modelled with PO data showed decreasing trends of Turtle Dove presence probabilities when forest coverage was higher than 60% (Fig. 4).

### Probability maps

**Figure 5:**
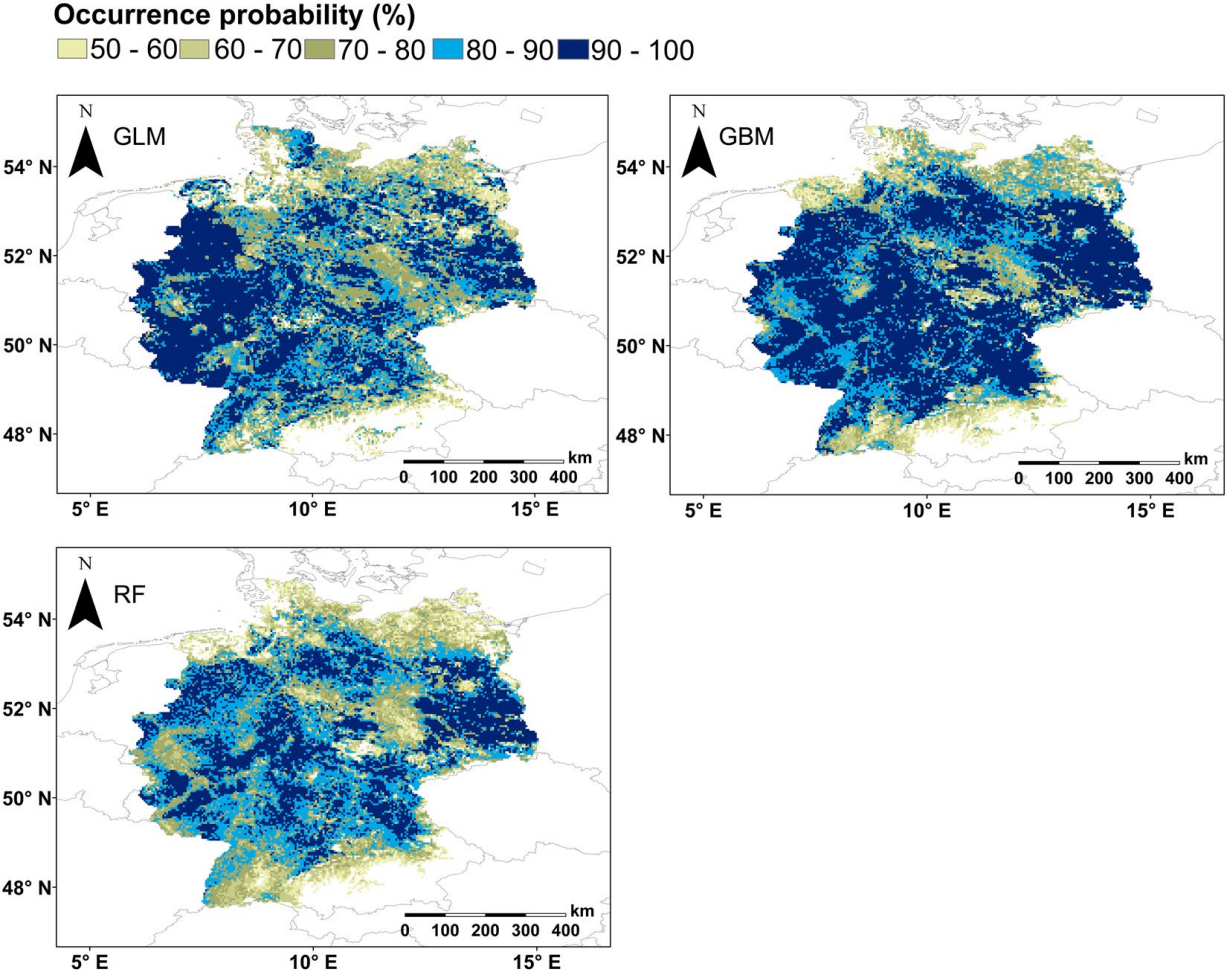
Probability maps generated for three species distribution models of Turtle Doves in Germany run with PA data. Only the areas with a probability ≥0.5 are presented. Probabilities of ≥0.8 were highlighted in blue shades and represent most likely regions for Turtle Dove occurrences and therefore those where adjusted land management would likely support breeding success of the species.

**Figure 6:**
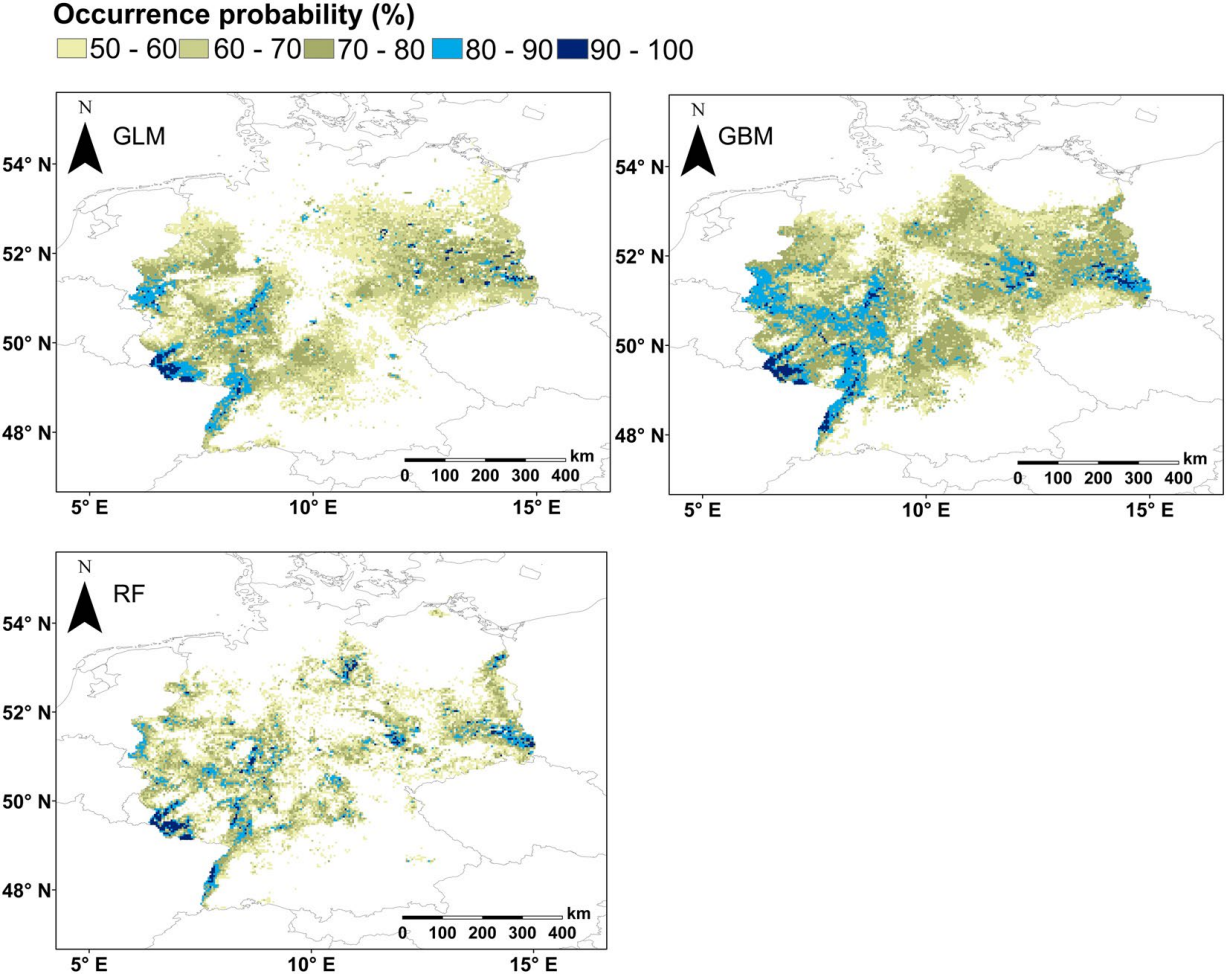
Probability maps generated for three species distribution models of Turtle Doves in Germany run with PO data. Only the areas with a probability ≥0.5 are presented. Probabilities of ≥0.8 were highlighted in blue shades and represent most likely regions for Turtle Dove occurrences and therefore those where adjusted land management would likely support breeding success of the species.

Probability maps for all models (Figs 5 and 6) highlighted areas with zero or little occurrence probability (<0.5) in the mountainous regions of Germany, but only maps generated for algorithms run with PO data also excluded coastal regions (Fig. 6). Additionally, maps for PO data indicated fewer regions with occurrence probabilities > 0.5 than the ones created with PA data. Furthermore, key breeding areas with probabilities > 0.8 were much smaller in maps with PO than with PA data (Figs 5 and 6). Generally, maps created with PA data indicated almost all of Germany, except for the south, as areas with high Turtle Dove occurrence probability (Fig. 5). PO based model probability maps mainly highlighted regions with high occurrence probabilities near Saarbrucken, west of Dusseldorf, in the lowlands of the Black Forest and in Lusatia (Fig. 6).

## Discussion

In this study, we were able to use two different Turtle Dove datasets (PA and PO) from Germany and run five different SDM algorithms to identify important climatic and land cover variables affecting species’ occurrence and we highlighted areas with high species presence probabilities. Evaluation of model performances unveiled an inadequacy of CTA and SRE algorithms for both species datasets (PA and PO). Generally, GBM and GLM performances for both species datasets were similar, but RF ran best with PO data.

Evaluation of variable importance and the corresponding response plots revealed a dependency of Turtle Dove presence on climatic variables. However, it was also obvious that, depending on the species dataset used, the importance of variables changed across model algorithms. Importance values of variables obtained from algorithms run with PO were usually higher than for the same variables obtained by the same algorithms run with PA. Furthermore, response plots for PO data showed clearer effects of variables on Turtle Dove occurrence probability than graphs created with PA data, because those mainly depicted constant response curves. This probably relates to higher numbers of species records in the PO than in the PA dataset, which might represent more evaluable data for modelling algorithms^46^.

Bio 6 codes the minimum temperature of the coldest month, which is January in Germany^46^. Regions with modelled favoured temperatures of 1–4 °C in January cover most of Germany except for the regions west of Bremen and north of Dusseldorf, alpine regions, as well as the mountainous areas Harz Mountains and Thuringian forest^46^. Those temperatures support the survival of food sources (weeds, seeds and cereals)^47–49^ and hedges as nesting sites, because temperatures lower than 0 °C (and higher than 40 °C) can lead to plant damage due to inhibited physiological processes^50^. Furthermore, temperatures between 0 °C and 10 °C are positively affecting hardening and frost resistance due to cold-acclimation and vernalisation^51–53^. Additionally, the highlighted temperatures were shown to kick-start the development of phenologically early stages of wheat, rape and other crop species^54,55^, which may lead to sufficient food availability during the early breeding season.

Bio 18 might be an important factor, because it covers the warmest months July to September^56^. July is one of the major important breeding months of Turtle Doves^57^ with high energetic costs for the birds as both adults and chicks need to access food frequently. It seems sensible that Turtle Doves would occur in regions with fewer heavy rainfall events. Heavy rain can result in higher mortality rates especially during breeding periods (particularly high mortality rates of chicks and juveniles), longer sheltering periods of adults to regulate the body temperature of their offspring and thus higher energy demands on adults, but also reduced feeding efficiency^58^. These effects have been demonstrated in raptors, Eagle Owls (*Bubo bubo*) and White Storks (*Ciconia ciconia*)^59–63^. Suitable regions with precipitation rates lower than 225 mm cover the western border of Germany, regions around Saarbrucken, Wiesbaden, Mainz, Spessart, Rhoen, the lowlands of the Thuringian Forest and Franconian Mountains, Leipzig, Dresden, Magdeburg as well as the Mecklenburg Lake District^46^.

Both climatic variables were able to describe a likely effect on the presence probability of Turtle Doves in Germany reasonably well, but further predictor variables can also heavily impact species occurrence, such as land type or soils^64–66^. For instance, ‘forest’ would be expected as an important land coverage variable due to its role as nesting site and shelter described in previous literature.^6,35,42,49,67–72^ However, only GLM with PA data demonstrated a positive effect on the occurrence probability when coverage was >40% and algorithms run with PO data highlighted negative effects when coverage was >60%. Mainly, forest coverages >60% are distributed in mountainous regions, e.g. the Spessart, the Alps, the Harz Mountains, the Thuringian Forest and the Erz Mountains^46^. The results for PO data were likely driven by the larger distribution and higher numbers of presences in regions with forest coverages of 20–60%^18^ than were presence records in PA data (Fig. 1). Furthermore, a forest coverage of >60% might devaluate a Turtle Dove habitat and can negatively affect species occurrence probability, because the spatial availability of feeding sites (farmland or pastures^6^) is reduced.

Probability maps created with PA and PO data showed pronounced differences (Figs 5 and 6). Coinciding with more or less constant response curves created with PA data, resulting probability maps also revealed little variation for Turtle Dove occurrence. Possible key breeding regions in Germany cannot be distinguished clearly and PA data probability maps are hard to interpret.

Probability maps drawn for PO data clearly distinguished areas with likelihoods of >0.8 (Fig. 6). Those areas are of interest for conservation management due to their likely importance for Turtle Dove occurrence. They combine the existence of optimum values for all three variables that were of importance in algorithms. Optimum values include minimum temperature in January between 1 °C – 4 °C (Bio 6), precipitation <225 mm during the breeding months (Bio 18) and forest coverage <60%^18,57^. Therefore, probabilities for survival of food sources, existence of nesting sites and high likelihood for survival of offspring may be highest in the highlighted regions^6,35,42,49,51–55,67–72^.

## Conclusions

Generally, model qualities, response curves and probability maps drawn for PA and PO data were different, but results agreed regarding the importance of climatic variables Bio 6 and Bio 18. Previously, climatic variables were used to describe species distributions reasonably well on large scales, either for an entire country like Finland^73^ or for a continent such as Europe^74,75^. In this study however, only PO data probability maps were able to specify regions of probably high conservation interest, although in other studies this type of data over-predicted the potential occurrence of species^31,76–78^. Regarding the different approaches with two datasets (PA and PO), citizen-based PO records might become of higher importance for suitability models in the future due to the strong decline of Turtle Doves. This might also be applicable for other scarce species.

Here, modelled important climate variables confirm the description of favoured dry and warm summer conditions of Turtle Doves in central Europe^49^. The temperature in January seems to play an important role for the survival and development of food sources as well as hedges and woodland as nesting sites^50–55^. These conditions support frequent feeding of offspring, prevention of hypothermia and reduced mortality rates of chicks, leading to higher nesting success of Turtle Doves. Although the importance of forests as nesting sites has been shown in other studies^6,35,42,49,67–72^, other land coverage variables did not prove to be of highest importance.

Differences between algorithms run with PA and PO data are likely linked to the wider distribution (Fig. 1) and larger numbers of species records in the PO dataset as larger sample sizes usually result in better model accurracy^46^. This supports the assumption that PO data (e.g. citizen-based) can serve as a good data basis and might become of higher importance for suitability models in the future, especially with regard to declining or rare species. Furthermore, data filtering according to e.g. accuracy of recorded location, breeding time and species’ territory size^79^, as we did here, probably constituted to the quality of used species records and reduced over-representation of one habitat^32^. Additionally, the inclusion of pseudo-absences was already shown to improve model quality when using PO data^31,32,79^, and probably also enhanced SDM quality in the present study.

While land coverage categories did not prove to be of highest importance in SDM and climate variables were able to describe the species distribution, but did not present detailed information about the specific characteristics of species’ habitats, future SDMs may be improved by the introduction of further variables. Generally, a major change of landscape types and their distribution did not occur across Germany, but management procedures, especially in agriculture, have been modified^35–37,39^. For instance, the intensified usage of herbicides or loss of field margins led to habitat loss and changes in availability and quality of food^35–39^. Therefore, variables like specified land coverage (e.g. forest edge, hedges), soil types, or information about agricultural management (e.g. usage of herbicides, existence of field margins or herbal vegetation, type of corn, conventional or organic farming, timing of harvest)^6,80–82^ could be added. To our knowledge, datasets containing suggested information are not available throughout Germany and investigation of all study sites from PO and PA datasets would require immense effort. Therefore, data could be gained on a smaller (territory sized) scale and conclusions could be drawn for Turtle Dove territories in Germany and implemented in management plans.

Suggestions for Turtle Dove supportive farming practices are already described in previous literature: A delay of harvest times until the end of August could improve the availability of food sources to raise late Turtle Dove broods^35^. Provision of supplementary food in the form of weeds and seeds would support a good body condition of adult Turtle Doves especially in the early breeding season^80,83^. These could be made available through e.g. unfertilised crop field margins, which favour diverse vegetation coverage^81,82^. It has also been shown that chicks in good conditions were mainly fed with seeds from arable plants^80^. This is why foraging sites of Turtle Doves should provide differing weeds and seeds of arable plants for adults and youngs^48,49,50,80^. Also, small patches with variable crops, peas and herbal sites with changing crop rotation and without usage of fertilizers to enrich the food plant diversity^35,81,82^ have been suggested to support Turtle Doves during the breeding season.

## Methods

### Study Species

Turtle Doves are migratory, granivorous birds that feed on weeds and seeds on the ground in agricultural landscapes and build their open nests preferably near the foraging site in protected vegetation such as dense bushes and hedges with a height of at least 4 m, and in forests^6,35,42,49,67–72^. Breeding pairs usually take up territories of ca. 1 km², but in some regions up to four pairs per km² have been recorded^49^. In general, they tend to breed in warm, temperate regions up to an altitude of 500 m in continental Europe^49,67^.

### Databases for environmental variables

Environmental variables consisted of climate and land cover data for Germany. Climate variables were downloaded from www.worldclim.org^18^. They were trimmed to the extent of Germany and exported as ascii files in DIVA-GIS following the tutorial for preparation of worldclim files for use in maxent (http://www.lep-net.org/wp-content/uploads/2016/08/WorldClim_to_MaxEnt_Tutorial.pdf)^84^. Corine land cover data (CLC 2006) for Germany came from the European Environment Agency (http://www.eea.europa.eu/legal/copyright)^85^. CLC 2006 codes 37 land cover categories.

ArcGIS 10.2.2 was used to create a fishnet, which based on the extent of trimmed climate grids and equalized the raster cell sizes of both environmental datasets. Therefore, land cover data and the fishnet were intersected. Area sizes and percentages of every land cover variable in each raster cell were calculated and joined to the fishnet according to raster cell IDs in ArcGIS 10.2.2. Furthermore, values of every climate variable in each raster cell were added to the attribute table. The final fishnet was opened in DIVA-GIS to create ascii files for each land cover variable.

All variables were checked for multi-collinearity. For that, we used the vif-function (variance inflation factor) in R 3.3.3^86^, which is embedded in the car-package, to check for collinearity between all variables included in the fishnet attribute table and excluded the ones with a vif >8. The procedure was repeated until all remaining variables had a vif of <8. The minimum threshold for vif to exclude collinearity is 10^87,88^, but values lower than 10 are assumed to be more precise^87^.

The resulting variable set was applied to each model. It consisted of seven landscape variables and eight climatic variables (Table 2). Land cover variables contained following categories: ‘urban areas’ included 11 categories (codes 111 to 142), ‘permanent cultures’ contained two categories (codes 221 and 222), ‘pasture’ contained only the category 231, ‘forest’ included three variables (codes 311 to 313), ‘herbs and shrubs’ contained three categories (codes 321 to 324), ‘no/little vegetation’ had also three categories (codes 331 to 333) and ‘wet areas’ contained nine categories (codes 411 to 523).

### PA data

The ‘Monitoring of breeding birds’ database contained PA data from 2005 to 2013. For this monitoring, 1394 study areas of the size of 1 km² are randomly distributed across Germany. Monitoring of these sites should be done annually by volunteering observers following a standardised field protocol. The protocol defines four surveys of the sites from March to June by one observer. Surveys start at sunrise under good weather conditions (no rain, low wind speed) and last for two to four hours. Observers follow a strict route of 3–4 km and record all breeding and territorial birds with registry of the position. Species that were not detected during surveys are noted as absent. When study sites were not checked, no data entries were recorded. Here, 1023 study areas contained no data entries throughout the available years, and were deleted. Therefore, we kept only those sites with at least one presence or absence data point during the years 2005 to 2013, obtaining a dataset of 371 sites, which was used for modelling. 293 sites had at least one Turtle Dove presence record and thus were accounted as Turtle Dove habitat. 78 studied sites were recorded for Turtle Dove absence. After filtering, monitoring places in 13 different states remained for model analyses (Fig. 1, Supplementary Table S1).

### PO data

The ornitho-dataset consisted of a total of 9064 PO records, in total. Observers can record the sighting localities with exact coordinates (exact locality), per district or municipality. In order to use the most accurate data, we filtered the ornitho dataset for exact localities and the breeding months June and July, because a previous ring re-encounter study showed that Turtle Doves are in their most northern distribution ranges, i.e. breeding areas, in June and July^56^. Furthermore, record locations were filtered for a minimum distance of at least 1 km between Turtle Dove records to remove over-sampled localities^32^ and to reduce possible multiple records of a single individual to only one data point, mirroring one record for one Turtle Dove territory (using ArcGIS 10.2.2, assuming a nest density of one per km^2^ ^49^). After filtering, the final dataset contained 1168 records from 14 states (Fig. 1, Supplementary Table S2).

To obtain an overview about spatial position of presence and absence points of PA and PO data we created another map containing the biggest cities and larger landscapes in Germany (Fig. 1d). Therefore, we depicted the position of cities and landscape names according to https://www.diercke.de/content/deutschland-physische-karte-978-3-14-100800-5-19-2-1^89^ in ArcGIS 10.2.2.

### Species distribution modelling

SDM was conducted using the Biomod 2 package (based on Biomod^64^) for R version 3.3.3^86^. Therefore, we mainly followed the SDM for Wolverines’ (*Gulo gulo*) current disctribution^87^ and the setups given in the package description^90^. Biomod 2 is able to build different model algorithms in one run for one species dataset^24,46,91^ and no expert knowledge is needed to determine the most appropriate modelling algorithm. Indeed, it is recommended to run a framework of different modelling algorithms^24^. The framework of present SDMs in Biomod 2 consists of three main modelling steps: (1) data formatting, (2) model computation and (3) projection of models^92^.

Biomod 2 was run for both, the PA and PO dataset separately, but the first step was equal for both datasets. Datasets were imported into Biomod 2. Then presence and absence or presence only data were defined for each location and environmental parameters were introduced as raster files following^92^. Raster files were stacked and then data were formatted according to the species dataset.

Data formatting for PA data was done using default settings as described in^92^. For PO data, we set the number of pseudo-absences to 1500 and Biomod 2 generated 949, thus the number of pseudo-absences was similar to the number of presences from the PO dataset. Pseudo-absence points had a minimum distance of at least 1 km to presence records and were created using the ‘disk’ algorithm^92^. The distance factor and the number of pseudo-absences were used to avoid pseudo-replication and also to prevent absences describing the same niche as presences (false absences)^90,93^.

Then, five different SDM algorithms were run for both datasets (PA and PO). The algorithms included were: Generalised Linear models (GLM), Generalised Boosted models (GBM), Classification Tree analysis (CTA), Surface Range Envelope (SRE similar to BIOCLIM) and Random Forests (RF). For model calibration, 70% of the data were used. The remaining data were used to test the models^91^. Every model algorithm was run 100 times.

For model evaluation, we calculated AUC-values of algorithms using the fBasics package^94^ and sensitivity and specificity using the get_evaluations function described in^92^ for both species datasets. Implemented variables were evaluated by variable importance and response curves, which were calculated and created with the associated functions embedded in Biomod 2^46,91,92^. The variable importance is given as a value between 0 and 1 with 1 as the highest possible value. The higher the value of a specific variable, the higher is its influence on the model. However, the calculation technique for variable importance (Pearson’s correlation) does not account for interactions between implemented variables and hence does not sum up to 1^46^. Response curves demonstrate the quantitative relationship between environmental variables and the logistic probability of the presence of the species (habitat suitability). Habitat probabilities per model algorithm were projected in Biomod 2^92^ using AUC as filter method. Then projections were stacked and subsets for each algorithm were generated. Model averages were built for every algorithm and according raster files were exported as ascii format. Final maps were generated in ArcGIS 10.2.2 for each model. We created maps depicting only those areas that had an occurrence probability of ≥0.5. Furthermore, areas with a probability ≥0.8 were considered key sites worthy of special management.

## Electronic supplementary material

Supplementary S1

Supplementary S2

## Data Availability

All data generated or analysed during this study are included in this published article and its Supplementary Information files. The raw datasets are from the DDA and restrictions apply to these data, which is why they are not publicly accessible, but can be obtained upon reasonable request and with permission from DDA and its teams responsible for the ‘Monitoring of breeding birds’ scheme and ornitho-data (Supplementary data S1 ‘Monitoring of breeding birds’ request date: 28.10.2014, permission date: 26.05.2015; Supplementary data S2 ornitho.de application numbers 2013.006 and 2013.006a from 29.01.2014 and updated at 06.11.2014).
