# Supplementary material for: Species distribution models of European Turtle Doves in Germany are more reliable with presence only rather than presence absence data
Source: Sci Rep. 2018 Nov 15;8:16898. doi: 10.1038/s41598-018-35318-2 (PMC6237818; doi:10.1038/s41598-018-35318-2)
Supplement: Supplementary file 1 — Supplementary S1 [file 41598_2018_35318_MOESM1_ESM.pdf]

Species distribution models of European Turtle Doves in Germany are more reliable with presence only rather than presence absence data

Melanie Marx[1*], Petra Quillfeldt[1]

Supplementary Table S1: List of analysed PA data from the 'Monitoring of breeding birds' dataset (Supplementary data S1_Table_PA data.pdf).

FID	code_site	lat	long	2005	2006	2007	2008	2009	2010	2011	2012	2013	PA
1	bb106	52.5909	13.8736	NA	1	0	0	0	0	0	0	0	1
2	bb111	52.6789	13.5105	NA	0	1	0	0	0	0	0	NA	1
3	bb118	52.6399	14.0531	NA	1	1	1	1	1	1	0	0	1
4	bb121	52.5177	14.4219	NA	NA	NA	0	0	0	0	1	0	1
5	bb124	52.4963	14.6313	NA	0	0	1	0	0	0	0	0	1
6	bb125	52.2437	12.5185	NA	0	0	0	0	0	1	0	0	1
7	bb128	52.2084	12.4621	NA	NA	1	0	0	0	1	0	0	1
8	bb129	52.1476	12.4108	NA	NA	0	0	0	1	0	0	0	1
9	bb138	52.1886	12.7867	NA	0	1	1	1	0	0	0	0	1
10	bb14	53.4562	13.9008	0	0	0	0	0	0	0	0	0	0
11	bb141	52.0706	13.1118	NA	0	0	NA	NA	0	1	0	1	1
12	bb142	52.0498	13.0586	NA	1	1	NA	NA	1	0	1	0	1
13	bb143	52.026	12.8493	NA	1	0	1	0	1	0	1	1	1
14	bb144	52.0191	13.2978	NA	NA	1	1	1	NA	NA	NA	NA	1
15	bb145	52.0166	13.2559	NA	NA	0	1	1	NA	NA	NA	NA	1
16	bb146	52.0131	13.2018	0	0	0	0	0	0	0	0	0	0
17	bb147	52.0368	13.118	NA	NA	0	0	0	0	0	0	1	1
18	bb152	52.1685	13.8503	NA	0	1	1	1	1	NA	1	1	1

FID	code_site	lat	long	2005	2006	2007	2008	2009	2010	2011	2012	2013	PA
19	bb153	52.0183	13.4438	NA	NA	1	1	0	1	1	1	1	1
20	bb160	52.0332	13.4201	NA	NA	0	1	0	1	1	0	0	1
21	bb164	52.2691	14.5805	NA	0	1	1	0	0	0	0	1	1
22	bb165	52.0446	14.4663	NA	1	1	1	1	1	0	1	1	1
23	bb168	52.1266	14.4114	NA	0	1	1	0	0	0	0	0	1
24	bb169	52.0272	14.6079	NA	NA	NA	1	1	0	NA	0	0	1
25	bb175	51.6212	13.0977	NA	NA	1	NA	NA	NA	NA	NA	NA	1
26	bb176	51.9981	13.9828	NA	1	0	0	0	1	NA	0	0	1
27	bb180	51.7906	13.9851	NA	NA	0	0	1	0	1	1	1	1
28	bb181	51.8535	13.6125	NA	0	1	0	0	0	0	0	0	1
29	bb185	51.6296	13.4142	NA	0	0	0	NA	1	NA	NA	NA	1
30	bb188	51.8772	14.3018	NA	0	NA	1	NA	0	0	0	NA	1
31	bb189	51.9266	14.2909	NA	NA	1	0	0	1	NA	NA	1	1
32	bb19	53.2928	13.5934	NA	NA	NA	1	1	0	1	0	1	1
33	bb190	51.9103	14.2564	NA	NA	1	1	1	1	NA	1	1	1
34	bb192	51.8245	14.4801	NA	0	0	1	0	0	0	0	0	1
35	bb195	51.7929	14.5413	0	0	0	0	0	0	0	0	0	0
36	bb198	51.6323	14.483	0	0	1	0	0	NA	0	0	NA	1
37	bb200	51.6312	14.0557	NA	NA	1	1	0	0	1	1	1	1
38	bb201	51.611	14.0469	NA	1	1	0	1	0	1	1	0	1
39	bb203	51.4455	13.2905	NA	NA	1	1	1	1	1	0	1	1
40	bb204	51.5537	13.987	NA	0	1	0	1	1	0	NA	0	1
41	bb206	51.4855	13.8033	NA	1	0	0	0	0	0	0	NA	1
42	bb21	53.2887	13.6218	1	NA	NA	NA	0	0	1	1	0	1
43	bb210	51.5805	14.2329	0	0	1	0	NA	NA	NA	NA	NA	1
44	bb221	52.4822	13.045	NA	NA	NA	0	0	0	1	0	0	1
45	bb225	53.0378	14.0442	0	0	0	0	0	0	0	0	0	0
46	bb245	52.4913	13.9512	NA	NA	NA	NA	NA	NA	1	1	NA	1

FID	code_site	lat	long	2005	2006	2007	2008	2009	2010	2011	2012	2013	PA
47	bb3	53.3389	12.1346	NA	NA	1	0	1	1	1	1	0	1
48	bb45	53.1447	12.7056	0	0	0	0	0	0	0	0	0	0
49	bb48	53.1218	12.7137	NA	1	0	0	0	0	0	0	0	1
50	bb5	53.3231	12.1078	NA	NA	1	0	0	0	1	0	0	1
51	bb6	53.2756	12.2194	NA	NA	0	0	1	0	0	1	1	1
52	bb62	53.1063	13.3468	NA	NA	NA	NA	0	1	0	0	0	1
53	bb70	52.9787	13.4637	NA	NA	NA	NA	1	0	1	1	0	1
54	bb81	52.8286	14.0353	NA	0	1	0	0	0	0	0	0	1
55	bb9	53.2739	12.303	NA	NA	1	0	0	0	0	0	0	1
56	bb96	52.7684	12.7355	NA	NA	0	1	0	0	0	NA	NA	1
57	bw10	48.1399	9.3561	1	1	1	1	0	0	1	1	NA	1
58	BW125	48.8443	9.2262	0	0	0	0	0	0	1	0	0	1
59	bw150	49.1527	10.1248	NA	NA	NA	NA	0	1	0	0	0	1
60	bw257	48.1354	7.6471	NA	1	1	0	0	0	0	0	0	1
61	bw262	48.0859	7.6007	NA	NA	NA	NA	1	1	0	0	0	1
62	bw264	48.0791	7.9939	0	0	0	0	0	0	0	0	0	0
63	bw268	48.0004	7.6771	1	1	1	1	1	1	1	1	1	1
64	bw276	48.2576	8.5116	0	0	0	0	0	0	0	0	0	0
65	bw298	48.235	8.7653	NA	NA	1	NA	NA	NA	NA	NA	NA	1
66	bw30	49.5556	8.4178	NA	NA	NA	NA	1	NA	1	NA	NA	1
67	bw358	47.9845	8.8318	0	0	0	0	0	0	0	0	0	0
68	BW36	49.3957	8.5113	NA	NA	NA	NA	NA	1	1	1	1	1
69	bw368	47.8116	9.0315	NA	NA	1	0	NA	NA	NA	NA	NA	1
70	bw42	49.4388	9.2533	NA	NA	NA	NA	NA	1	1	0	NA	1
71	bw52	49.2507	8.8547	NA	NA	1	NA	NA	NA	NA	NA	NA	1
72	bw58	49.5529	9.5596	NA	NA	1	0	0	1	NA	NA	NA	1
73	bw6	48.4546	9.3989	0	0	0	0	0	0	0	0	0	0
74	bw97	48.8063	8.1156	NA	0	1	0	0	0	0	0	0	1

**FID**	**code_site**	**lat**	**long**	**2005**	**2006**	**2007**	**2008**	**2009**	**2010**	**2011**	**2012**	**2013**	**PA**
75	by104	49.9896	12.3681	NA	NA	NA	NA	1	1	1	NA	NA	1
76	by109	49.5148	10.1106	0	0	0	0	0	0	0	0	0	0
77	by11	50.0595	9.0701	0	0	0	0	0	0	0	0	0	0
78	by110	49.5817	10.3425	1	1	0	0	0	1	1	0	0	1
79	by111	49.5017	10.5931	NA	NA	0	1	1	0	0	0	0	1
80	by114	49.3637	10.5806	NA	1	1	1	1	1	1	1	1	1
81	by117	49.2139	10.5233	NA	NA	NA	1	0	0	NA	NA	NA	1
82	by135	49.4232	11.9807	NA	NA	NA	1	1	1	0	NA	NA	1
83	by142	49.5782	12.4879	NA	NA	NA	NA	1	NA	NA	NA	NA	1
84	by152	49.288	12.9211	0	0	0	0	0	0	0	0	0	0
85	by153	49.0942	10.6377	0	0	0	0	0	0	0	0	0	0
86	by154	48.8819	10.4465	0	0	0	0	0	0	0	0	0	0
87	by163	48.9701	11.2467	NA	1	NA	1	0	0	0	0	0	1
88	by171	49.022	11.3991	0	0	0	0	0	0	0	0	0	0
89	by172	48.9216	11.5718	NA	0	0	0	0	1	1	1	1	1
90	by175	48.8491	11.379	NA	NA	1	1	NA	NA	NA	NA	NA	1
91	by180	49.1787	12.0894	NA	NA	NA	0	1	0	0	NA	NA	1
92	by182	49.1002	12.0614	NA	NA	NA	0	1	1	0	NA	NA	1
93	by184	49.0327	12.3779	NA	NA	0	NA	1	NA	NA	NA	NA	1
94	by19	50.0273	9.9162	NA	NA	NA	NA	0	0	1	0	NA	1
95	by193	49.019	13.2663	0	0	0	0	0	0	0	0	0	0
96	by199	48.9673	13.5353	0	0	0	0	0	0	0	0	0	0
97	by225	48.6497	11.4111	NA	1	0	0	0	0	0	0	0	1
98	by241	48.6383	12.1735	NA	NA	1	1	1	0	0	1	0	1
99	by251	48.6744	12.735	NA	1	1	1	0	0	1	1	NA	1
100	by27	50.103	10.3885	NA	NA	NA	0	0	1	0	0	0	1
101	by271	48.1987	10.4949	0	0	0	0	0	0	0	0	0	0
102	by28	50.1307	10.2425	NA	0	0	1	0	0	NA	NA	NA	1

FID	code_site	lat	long	2005	2006	2007	2008	2009	2010	2011	2012	2013	PA
103	by309	48.1232	12.6352	0	0	0	0	0	0	0	0	0	0
104	by32	50.0646	10.2149	NA	NA	0	0	0	0	0	1	0	1
105	by34	50.0424	10.0971	NA	0	0	1	0	0	0	0	0	1
106	by350	47.6536	11.1933	0	0	0	0	0	0	0	0	0	0
107	by351	47.6376	11.1678	0	0	0	0	0	0	0	0	0	0
108	by36	50.2956	10.8018	NA	NA	NA	NA	0	0	0	0	1	1
109	by367	47.7629	11.7344	0	0	0	0	0	0	0	0	0	0
110	by369	47.7009	11.3529	0	0	0	0	0	0	0	0	0	0
111	by372	47.6415	11.8075	0	0	0	0	0	0	0	0	0	0
112	by402	47.4922	10.1611	0	0	0	0	0	0	0	0	0	0
113	by408	47.375	10.3635	0	0	0	0	0	0	0	0	0	0
114	by414	47.5036	11.0778	0	0	0	0	0	0	0	0	0	0
115	by415	47.5937	10.9801	0	0	0	0	0	0	0	0	0	0
116	by420	47.5408	10.9429	0	0	0	0	0	0	0	0	0	0
117	by421	47.5382	11.0495	0	0	0	0	0	0	0	0	0	0
118	by434	47.5119	11.4914	0	0	0	0	0	0	0	0	0	0
119	by435	47.485	11.3437	0	0	0	0	0	0	0	0	0	0
120	by452	49.3712	12.1852	NA	NA	NA	NA	1	0	0	0	NA	1
121	by453	49.5721	11.374	0	0	0	0	0	0	0	0	0	0
122	by461	47.4558	10.3757	0	0	0	0	0	0	0	0	0	0
123	by475	47.5747	12.7966	0	0	0	0	0	0	0	0	0	0
124	by476	47.5474	12.8834	0	0	0	0	0	0	0	0	0	0
125	by478	47.5707	12.8993	0	0	0	0	0	0	0	0	0	0
126	by486	47.5214	12.8968	0	0	0	0	0	0	0	0	0	0
127	by5	50.4557	10.3226	NA	1	0	0	0	NA	NA	NA	NA	1
128	by62	49.684	9.0929	NA	1	1	NA	NA	NA	NA	NA	NA	1
129	by65	49.854	9.7747	NA	0	0	0	0	1	0	0	0	1
130	by66	49.8424	9.5561	NA	NA	NA	NA	0	0	0	0	1	1

FID	code_site	lat	long	2005	2006	2007	2008	2009	2010	2011	2012	2013	PA
131	by69	49.979	10.2835	NA	1	1	1	1	1	1	1	1	1
132	by7	50.4094	10.3007	NA	NA	1	0	0	0	NA	NA	NA	1
133	by72	49.8806	10.1991	NA	NA	NA	1	0	0	1	0	1	1
134	by74	49.8078	10.2954	NA	NA	NA	0	0	1	0	0	1	1
135	by75	49.8383	10.1922	NA	NA	NA	NA	NA	NA	1	0	0	1
136	by80	49.6742	10.3087	0	0	0	0	0	0	0	0	0	0
137	by87	49.7608	11.2069	0	0	0	0	0	0	0	0	0	0
138	by91	49.6165	11.2798	NA	NA	NA	NA	NA	1	0	0	0	1
139	by98	49.8104	11.7647	NA	NA	NA	NA	1	NA	NA	NA	NA	1
140	hb30	53.1476	8.6488	0	0	0	0	0	0	0	0	0	0
141	he100	50.5591	9.5698	0	1	1	0	0	0	0	0	0	1
142	he101	50.4962	9.5884	NA	NA	0	1	NA	0	NA	1	0	1
143	he111	50.337	8.197	1	1	1	1	NA	NA	NA	NA	NA	1
144	he115	50.1619	8.1516	0	1	1	NA	1	NA	NA	NA	NA	1
145	he116	50.1304	8.4385	1	1	NA	NA	NA	NA	NA	NA	NA	1
146	he119	50.0892	8.3123	0	0	0	0	0	0	0	0	0	0
147	he12	51.3399	8.9975	NA	1	0	0	0	0	0	NA	1	1
148	he120	50.0719	8.309	0	NA	NA	NA	NA	1	0	NA	NA	1
149	he122	50.0351	8.0329	NA	NA	NA	NA	NA	0	1	1	1	1
150	he124	50.0169	8.3674	NA	1	NA	NA	NA	NA	NA	NA	NA	1
151	he130	50.2646	8.6737	NA	NA	NA	NA	1	1	0	0	0	1
152	he137	50.0123	8.8535	0	0	1	NA	0	0	0	NA	0	1
153	he144	49.7547	8.5728	1	1	1	1	1	1	1	1	1	1
154	he150	49.8149	8.9269	0	1	0	0	NA	0	0	0	0	1
155	he16	51.3017	9.0916	1	NA	1	NA	NA	NA	NA	NA	NA	1
156	he38	51.0058	8.6059	0	NA	1	1	0	NA	NA	NA	NA	1
157	he42	51.1849	9.1196	0	0	0	0	0	0	1	0	0	1
158	he44	51.0706	8.6719	0	NA	1	0	1	NA	1	NA	NA	1

FID	code_site	lat	long	2005	2006	2007	2008	2009	2010	2011	2012	2013	PA
159	he46	51.0348	8.6746	0	1	0	0	0	0	0	NA	NA	1
160	he47	50.987	8.9789	0	0	0	1	NA	NA	NA	NA	1	1
161	he51	50.8317	9.1943	1	0	0	0	0	0	0	NA	0	1
162	he52	50.8031	8.8527	0	0	0	0	0	0	0	0	0	0
163	he70	50.6975	8.5851	0	0	1	NA	NA	NA	NA	NA	NA	1
164	he77	50.4342	8.4539	0	0	0	1	NA	NA	NA	NA	NA	1
165	he82	50.7323	8.7186	0	0	0	0	0	0	0	0	0	0
166	he84	50.6877	9.261	0	0	1	1	1	1	1	NA	1	1
167	he85	50.6782	8.9495	0	1	0	0	NA	0	0	0	0	1
168	he86	50.6421	8.7811	NA	NA	NA	NA	0	0	1	0	0	1
169	he9	51.3974	9.1202	NA	0	0	NA	NA	0	0	1	0	1
170	he93	50.5156	8.9599	NA	NA	NA	NA	NA	0	1	0	0	1
171	he94	50.4797	8.6972	NA	1	0	NA	NA	NA	NA	NA	NA	1
172	he96	50.7051	9.9068	0	0	0	0	0	1	0	0	0	1
173	mv109	53.7069	14.1737	0	0	0	0	0	0	0	1	0	1
174	mv121	53.5629	11.419	0	0	0	0	0	0	0	0	0	0
175	mv122	53.4418	11.5234	0	0	0	0	0	0	0	0	0	0
176	mv123	53.4067	11.3614	NA	0	0	0	0	1	0	1	0	1
177	mv124	53.4023	11.9548	0	0	0	0	0	0	0	0	0	0
178	mv126	53.3862	11.4193	NA	1	1	1	0	1	0	0	0	1
179	mv158	53.5607	14.184	0	0	0	0	0	0	0	0	0	0
180	mv164	53.5363	13.3572	0	0	0	0	0	0	0	0	0	0
181	mv24	54.1003	12.491	NA	NA	NA	NA	0	0	1	NA	NA	1
182	mv40	54.1291	13.4042	0	0	0	0	0	0	0	0	0	0
183	mv47	53.866	10.7965	0	0	0	0	0	0	0	0	0	0
184	mv53	53.8369	11.7171	NA	1	NA	NA	NA	NA	NA	NA	NA	1
185	mv92	53.9898	13.773	0	0	0	0	0	0	0	0	0	0
186	ni106	51.3395	9.6961	1	NA	NA	NA	NA	NA	NA	NA	NA	1

FID	code_site	lat	long	2005	2006	2007	2008	2009	2010	2011	2012	2013	PA
187	ni128	53.5639	6.7111	0	0	0	0	0	0	0	0	0	0
188	ni134	53.3505	7.8463	NA	NA	0	0	1	0	NA	NA	NA	1
189	ni136	53.3903	7.9479	NA	0	1	0	0	0	NA	NA	NA	1
190	ni146	53.2506	8.0465	1	1	1	1	1	1	0	1	0	1
191	ni150	53.5443	9.2651	1	0	0	0	0	0	0	0	0	1
192	ni166	53.3361	9.6897	NA	1	0	0	0	0	0	0	0	1
193	ni167	53.2457	9.5901	1	1	1	0	0	0	0	0	0	1
194	ni185	52.8606	7.8559	0	0	0	1	NA	NA	NA	NA	NA	1
195	ni186	52.8378	7.4297	1	1	1	0	1	0	0	0	0	1
196	ni191	53.0184	8.2491	0	0	1	0	0	0	0	0	NA	1
197	ni193	52.832	8.586	NA	NA	NA	NA	0	1	1	1	1	1
198	ni196	53.0444	9.2645	0	1	0	1	0	0	0	NA	NA	1
199	ni198	53.1941	9.7997	NA	0	1	0	0	0	0	0	0	1
200	ni2	53.8124	8.5673	0	0	0	0	0	0	0	0	0	0
201	ni203	53.1057	9.8311	NA	NA	NA	NA	0	0	0	0	1	1
202	ni218	52.9964	10.3383	NA	1	1	1	1	NA	NA	NA	NA	1
203	ni225	52.9726	10.9564	NA	1	1	1	0	1	1	0	0	1
204	ni227	52.9831	11.2877	1	NA	NA	NA	NA	NA	NA	NA	NA	1
205	ni228	53.0141	11.3385	NA	1	1	1	1	NA	NA	NA	NA	1
206	ni24	53.2683	10.1617	1	1	0	0	0	0	0	NA	NA	1
207	ni240	52.7456	8.4088	1	0	0	1	0	1	0	0	0	1
208	ni241	52.638	8.6059	1	NA	NA	NA	NA	NA	NA	NA	NA	1
209	ni243	52.5764	8.6405	1	0	0	NA	0	0	0	0	NA	1
210	ni246	52.6863	9.3296	1	1	1	1	1	1	NA	1	1	1
211	ni248	52.665	9.0801	NA	NA	NA	NA	0	1	1	1	1	1
212	ni252	52.5022	8.7647	NA	NA	1	1	0	1	1	0	0	1
213	ni260	52.687	9.4181	1	1	1	1	1	1	1	NA	NA	1
214	ni263	52.5154	9.5626	1	1	1	1	1	1	1	0	0	1

FID	code_site	lat	long	2005	2006	2007	2008	2009	2010	2011	2012	2013	PA
215	NI28	53.1679	10.9667	0	NA	NA	0	0	0	1	1	1	1
216	ni285	52.2156	9.6265	1	0	0	0	0	0	0	0	0	1
217	ni288	52.0348	9.3407	NA	0	1	NA	NA	NA	NA	NA	NA	1
218	ni298	51.8328	9.6073	NA	NA	NA	NA	1	1	1	0	1	1
219	ni37	53.0798	7.8583	0	0	0	1	0	0	0	NA	NA	1
220	ni38	53.0697	10.2459	1	1	0	1	0	NA	NA	NA	NA	1
221	ni43	52.9791	7.6822	0	0	0	0	0	0	0	0	0	0
222	ni435	52.7323	10.1005	NA	0	0	1	1	0	0	0	0	1
223	ni49	52.8686	7.4628	1	1	1	1	1	1	0	1	1	1
224	ni56	52.8061	10.5053	1	0	NA	NA	NA	NA	NA	NA	NA	1
225	ni57	52.7743	10.1337	1	1	1	NA	NA	NA	NA	NA	NA	1
226	ni59	52.7581	9.9258	1	0	NA	0	NA	NA	NA	NA	NA	1
227	ni66	52.626	9.4652	0	0	0	0	0	0	0	0	0	0
228	ni71	52.519	8.8453	0	1	1	1	1	0	1	0	1	1
229	NI76	52.3928	7.5088	NA	NA	NA	NA	0	1	0	0	0	1
230	ni78	52.3483	9.0807	1	1	1	1	1	NA	NA	NA	NA	1
231	ni86	52.1388	8.1893	0	0	1	NA	NA	NA	NA	NA	NA	1
232	ni87	52.1235	10.4677	0	0	0	0	0	0	0	0	0	0
233	ni88	52.1013	7.9856	0	0	1	0	NA	NA	NA	NA	NA	1
234	ni89	52.1027	9.8247	0	0	1	0	1	0	0	0	0	1
235	ni90	52.0714	10.3054	0	0	0	0	0	0	0	0	0	0
236	ni91	52.0665	9.8678	0	0	0	0	0	0	0	0	0	0
237	ni93	51.9881	10.5069	1	1	1	1	0	0	0	NA	NA	1
238	ni95	51.9248	10.5339	0	0	0	0	0	0	0	0	0	0
239	nw112	51.6012	8.4073	0	1	1	1	NA	NA	NA	NA	NA	1
240	nw13	52.1288	7.0192	NA	NA	NA	1	1	0	NA	NA	NA	1
241	nw130	52.0855	7.4491	NA	1	0	0	NA	0	0	NA	0	1
242	nw135	50.6537	7.0395	0	1	1	1	1	1	1	0	0	1

FID	code_site	lat	long	2005	2006	2007	2008	2009	2010	2011	2012	2013	PA
243	nw140	52.3085	8.4761	NA	1	0	0	0	0	0	0	NA	1
244	nw143	51.0321	6.2615	1	1	1	1	1	1	0	1	0	1
245	nw173	52.3535	8.5664	NA	0	0	0	0	1	0	0	0	1
246	nw183	51.2601	8.5722	NA	0	0	NA	0	0	1	0	NA	1
247	nw184	50.5589	6.4075	NA	NA	1	1	1	0	0	NA	NA	1
248	nw20	51.4248	6.2679	NA	1	1	1	1	NA	NA	NA	NA	1
249	nw25	52.0411	6.92	NA	1	0	1	1	0	NA	NA	NA	1
250	nw27	51.7163	6.8176	1	0	0	0	0	0	NA	NA	NA	1
251	nw91	51.2664	8.2284	NA	NA	NA	0	0	1	0	1	0	1
252	rp102	49.9723	8.1042	NA	NA	NA	NA	0	1	0	1	0	1
253	rp103	49.9652	8.08	NA	NA	NA	NA	NA	1	1	1	1	1
254	rp105	49.8916	8.3292	NA	NA	NA	NA	NA	NA	0	1	1	1
255	rp106	49.8558	8.2935	NA	1	NA	NA	NA	NA	NA	NA	NA	1
256	rp117	49.6562	8.1272	NA	NA	NA	NA	1	0	0	NA	NA	1
257	rp120	49.5856	7.5447	NA	NA	NA	NA	1	0	1	NA	NA	1
258	rp121	49.5116	7.9073	0	1	1	1	1	NA	NA	NA	NA	1
259	rp139	49.4517	8.1808	0	0	0	0	1	NA	NA	NA	NA	1
260	rp140	49.4314	8.2231	0	0	0	0	0	0	0	0	0	0
261	rp142	49.3412	8.3276	NA	NA	1	NA	NA	NA	NA	NA	NA	1
262	rp144	49.3255	8.1592	NA	NA	NA	NA	NA	1	NA	NA	NA	1
263	rp146	49.2471	8.2284	NA	NA	1	0	1	0	0	NA	NA	1
264	rp20	50.6072	7.8371	NA	NA	NA	NA	NA	NA	NA	NA	1	1
265	rp21	50.5499	7.8953	NA	0	0	NA	0	0	1	NA	0	1
266	rp24	50.4205	7.3563	0	0	0	0	0	0	0	0	0	0
267	rp4	50.6005	7.1496	0	0	0	0	0	0	0	0	0	0
268	rp5	50.6035	7.2248	NA	NA	NA	NA	NA	NA	0	NA	1	1
269	rp84	49.9517	7.7075	1	1	1	NA	NA	NA	NA	NA	NA	1
270	rp86	49.9295	7.8964	NA	NA	NA	NA	NA	NA	NA	NA	1	1

FID	code_site	lat	long	2005	2006	2007	2008	2009	2010	2011	2012	2013	PA
271	rp94	49.6876	7.8586	NA	NA	NA	NA	NA	NA	0	1	1	1
272	rp99	49.6526	7.7738	NA	NA	NA	NA	NA	NA	0	0	1	1
273	sh16	54.6688	8.2947	0	0	0	0	0	0	0	0	0	0
274	sh2	55.0506	8.4482	0	0	0	0	0	0	0	0	0	0
275	sh23	54.5334	8.9162	0	0	0	0	0	0	0	0	0	0
276	sh42	54.303	8.6149	0	0	0	0	0	0	0	0	0	0
277	sh47	54.2742	9.0759	0	0	0	0	0	0	0	0	0	0
278	sh51	54.1193	9.2989	NA	NA	0	0	0	1	0	0	0	1
279	sh53	54.3885	9.5414	NA	1	1	1	1	NA	NA	NA	NA	1
280	sh63	54.1048	9.3654	NA	NA	0	0	0	1	0	0	0	1
281	sh65	54.0947	9.4073	NA	NA	0	0	0	0	0	1	0	1
282	sh89	53.9765	9.679	NA	NA	0	1	0	0	1	1	0	1
283	sn11	51.4146	12.1949	0	0	0	0	0	0	0	0	0	0
284	sn111	51.0955	14.6871	NA	NA	NA	NA	NA	NA	NA	1	0	1
285	sn120	50.5118	12.5394	NA	NA	NA	0	0	0	0	0	1	1
286	sn121	50.5326	12.0649	NA	NA	NA	NA	NA	NA	NA	1	1	1
287	sn123	50.7939	13.2199	NA	NA	NA	NA	NA	NA	NA	NA	1	1
288	sn131	50.6568	12.8507	NA	1	1	0	1	NA	1	1	1	1
289	sn141	51.2257	12.6995	NA	1	0	0	NA	NA	NA	NA	NA	1
290	sn148	51.5159	12.725	NA	NA	NA	NA	NA	NA	NA	0	1	1
291	sn149	51.3071	12.8995	NA	NA	NA	NA	NA	NA	NA	1	1	1
292	sn15	51.2299	12.3968	NA	NA	NA	NA	NA	NA	0	1	NA	1
293	sn151	51.2836	14.5469	NA	NA	NA	NA	NA	NA	1	1	1	1
294	sn16	51.23	12.5903	NA	NA	NA	NA	0	NA	NA	1	0	1
295	sn160	50.9334	13.4637	NA	NA	NA	NA	NA	NA	1	NA	NA	1
296	sn164	51.0644	14.1904	NA	NA	NA	NA	NA	NA	1	1	1	1
297	sn28	51.3388	12.8785	NA	NA	NA	1	1	1	0	0	0	1
298	sn35	51.362	13.956	NA	NA	NA	NA	0	0	1	0	1	1

FID	code_site	lat	long	2005	2006	2007	2008	2009	2010	2011	2012	2013	PA
299	sn4	51.6111	13.0809	NA	NA	NA	NA	NA	NA	NA	1	1	1
300	sn44	51.2292	14.0328	NA	NA	1	NA	NA	NA	NA	NA	NA	1
301	sn45	51.2386	13.3967	NA	0	0	0	1	NA	NA	NA	NA	1
302	sn5	51.5928	12.6556	NA	1	NA	NA	NA	NA	NA	NA	NA	1
303	sn51	51.4069	14.4929	NA	1	NA	NA	NA	NA	NA	1	1	1
304	sn52	51.3953	14.1861	NA	NA	NA	NA	NA	NA	NA	1	1	1
305	sn54	51.293	14.6108	NA	NA	NA	NA	NA	NA	1	1	1	1
306	sn57	51.2742	14.2611	NA	NA	NA	NA	NA	NA	NA	NA	1	1
307	sn67	51.2824	14.9747	NA	NA	NA	NA	NA	NA	NA	1	1	1
308	sn69	51.2265	14.8596	NA	1	0	1	1	0	0	0	1	1
309	sn75	51.1224	12.7275	NA	NA	NA	NA	NA	NA	NA	1	1	1
310	sn8	51.5238	12.3013	0	0	0	0	0	0	0	0	0	0
311	sn83	50.8477	13.2528	NA	NA	1	1	1	NA	NA	NA	NA	1
312	sn9	51.5683	12.3918	NA	NA	NA	NA	1	0	1	1	1	1
313	st101	51.7148	11.9445	0	0	0	0	0	0	0	0	0	0
314	st108	51.636	11.4689	1	0	1	0	1	0	0	0	0	1
315	st109	51.9846	12.3294	0	0	0	0	0	0	0	0	0	0
316	st110	51.9475	12.2376	NA	NA	NA	NA	1	1	1	1	1	1
317	st114	51.7453	12.3537	NA	0	1	1	1	0	0	0	0	1
318	st121	51.9227	12.9247	0	1	1	1	1	1	1	1	1	1
319	st122	51.9478	12.6888	NA	1	0	0	0	1	NA	NA	NA	1
320	st124	51.8473	12.979	NA	NA	0	1	1	1	1	1	1	1
321	st125	51.8121	13.0876	1	1	1	1	0	0	0	0	0	1
322	st135	51.5406	11.716	1	0	1	0	0	0	1	0	0	1
323	st137	51.4723	11.6448	1	0	0	0	0	0	0	0	0	1
324	st139	51.5098	11.7948	NA	NA	NA	1	1	1	1	1	0	1
325	st140	51.5047	11.6844	1	1	1	1	0	1	1	1	1	1
326	st143	51.267	11.6018	NA	1	1	1	1	1	NA	NA	0	1

FID	code_site	lat	long	2005	2006	2007	2008	2009	2010	2011	2012	2013	PA
327	st145	51.2189	11.8629	NA	NA	NA	1	0	0	0	0	0	1
328	st150	51.1142	11.8121	NA	0	1	1	1	1	NA	NA	NA	1
329	st160	52.37	11.1938	NA	NA	NA	NA	NA	1	1	1	0	1
330	st167	51.8977	13.0107	NA	NA	NA	NA	0	1	1	1	1	1
331	st19	52.6061	11.1296	NA	NA	NA	NA	NA	NA	1	0	1	1
332	ST20	52.6102	11.0087	NA	NA	NA	NA	NA	NA	NA	1	1	1
333	st21	52.5467	11.0629	NA	NA	0	0	0	0	0	1	0	1
334	st26	52.6933	11.3999	NA	NA	0	0	0	1	0	0	0	1
335	st3	52.8515	11.3173	NA	NA	NA	NA	1	NA	NA	NA	NA	1
336	st30	52.5827	11.9413	NA	NA	NA	0	0	1	0	0	0	1
337	st32	52.4907	11.6332	NA	NA	1	1	1	1	0	1	0	1
338	st34	52.4849	11.9173	NA	NA	NA	1	0	1	1	1	0	1
339	st37	52.4666	11.5482	1	0	0	0	0	NA	0	0	0	1
340	st40	52.4717	11.629	0	0	0	0	0	0	0	0	0	0
341	st43	52.4777	11.9615	0	0	1	0	NA	0	0	NA	NA	1
342	st49	52.756	12.0526	0	0	0	1	0	0	0	0	0	1
343	st56	52.2549	11.0737	1	1	1	1	1	1	1	1	1	1
344	st60	52.0879	11.1843	NA	NA	1	1	1	1	0	1	1	1
345	st72	52.0617	11.3661	NA	NA	NA	NA	NA	NA	1	1	NA	1
346	st78	52.3661	12.1073	0	0	0	0	0	0	0	0	0	0
347	st82	52.0164	12.4552	NA	NA	0	0	0	1	0	0	0	1
348	st85	51.9537	11.1245	0	0	0	0	0	0	0	0	0	0
349	st87	51.981	10.9647	0	1	0	0	0	0	NA	NA	NA	1
350	st88	51.9758	11.0559	NA	NA	NA	NA	NA	NA	0	1	NA	1
351	st90	51.7447	10.9511	1	0	1	0	0	0	1	1	1	1
352	st92	51.7573	10.9089	0	0	1	1	0	0	1	1	0	1
353	th100	50.6935	10.9998	NA	NA	NA	NA	1	NA	0	0	0	1
354	th111	50.725	11.6265	NA	0	1	1	1	0	0	0	0	1

**FID**	**code_site**	**lat**	**long**	**2005**	**2006**	**2007**	**2008**	**2009**	**2010**	**2011**	**2012**	**2013**	**PA**
355	th112	50.6486	11.6316	1	1	1	1	0	0	0	0	0	1
356	th121	50.3812	10.766	NA	1	1	1	1	1	1	1	1	1
357	th123	50.9864	10.8524	0	0	0	0	0	0	0	0	0	0
358	th16	51.3887	10.1941	NA	NA	NA	NA	NA	0	0	1	0	1
359	th19	51.3123	10.1111	1	1	1	1	0	1	1	1	1	1
360	th27	51.492	10.9587	NA	1	1	NA	NA	NA	NA	NA	NA	1
361	th35	51.2313	10.9402	0	0	0	1	1	NA	NA	NA	NA	1
362	th39	51.0282	10.3359	NA	0	0	0	0	0	1	NA	0	1
363	th41	50.9417	10.2205	NA	0	1	0	1	0	0	0	0	1
364	th45	50.8391	10.2216	NA	1	1	1	1	1	1	1	1	1
365	th50	51.1893	11.2771	0	0	0	0	0	0	0	0	0	0
366	th61	50.8355	10.8805	0	1	1	0	1	1	1	1	1	1
367	th62	50.8195	10.8625	NA	1	1	1	1	1	0	1	1	1
368	th73	50.8057	11.5084	NA	NA	NA	NA	1	1	NA	0	1	1
369	th76	50.878	12.1884	0	0	0	0	0	0	0	0	0	0
370	th82	50.6617	10.4477	NA	NA	1	NA	NA	NA	NA	NA	NA	1
371	th99	50.7537	11.2215	NA	0	0	1	0	0	0	0	0	1
