# Supplementary material for: Species distribution models of European Turtle Doves in Germany are more reliable with presence only rather than presence absence data
Source: Sci Rep. 2018 Nov 15;8:16898. doi: 10.1038/s41598-018-35318-2 (PMC6237818; doi:10.1038/s41598-018-35318-2)
Supplement: Supplementary file 2 — Supplementary S2 [file 41598_2018_35318_MOESM2_ESM.pdf]

**Species distribution models of European Turtle Doves in Germany are more reliable with presence only rather than presence absence data**

Melanie Marx[1*], Petra Quillfeldt[1]

**Supplementary Table S2:** List of analysed PO data from the ornitho-dataset (Supplementary data S2_Table_PO data.pdf).

| **ID** | **species** | **Country** | **long** | **lat** | **PO** |
|---|---|---|---|---|---|
| 9734894 | *Streptopelia turtur* | Ausschließliche Wirtschaftszone | 7.89 | 54.18 | 1 |
| 2652133 | *Streptopelia turtur* | Hamburg | 10.18 | 53.61 | 1 |
| 409111 | *Streptopelia turtur* | Baden-Württemberg | 7.61 | 47.88 | 1 |
| 1733976 | *Streptopelia turtur* | Baden-Württemberg | 9.57 | 49.39 | 1 |
| 1826673 | *Streptopelia turtur* | Baden-Württemberg | 9.53 | 49.51 | 1 |
| 1832419 | *Streptopelia turtur* | Baden-Württemberg | 9.49 | 49.41 | 1 |
| 1842788 | *Streptopelia turtur* | Baden-Württemberg | 8.57 | 49.42 | 1 |
| 1867265 | *Streptopelia turtur* | Baden-Württemberg | 9.30 | 49.38 | 1 |
| 1875122 | *Streptopelia turtur* | Baden-Württemberg | 8.60 | 49.33 | 1 |
| 1886634 | *Streptopelia turtur* | Baden-Württemberg | 8.68 | 49.15 | 1 |
| 1893118 | *Streptopelia turtur* | Baden-Württemberg | 7.78 | 48.43 | 1 |
| 1897546 | *Streptopelia turtur* | Baden-Württemberg | 7.54 | 47.75 | 1 |

| **ID** | **species** | **Country** | **long** | **lat** | **PO** |
|---|---|---|---|---|---|
| 1920117 | *Streptopelia turtur* | Baden-Württemberg | 9.34 | 49.56 | 1 |
| 1961264 | *Streptopelia turtur* | Baden-Württemberg | 9.28 | 49.38 | 1 |
| 1972572 | *Streptopelia turtur* | Baden-Württemberg | 7.52 | 47.70 | 1 |
| 1996082 | *Streptopelia turtur* | Baden-Württemberg | 8.51 | 49.36 | 1 |
| 2040714 | *Streptopelia turtur* | Baden-Württemberg | 9.32 | 49.56 | 1 |
| 2243044 | *Streptopelia turtur* | Baden-Württemberg | 7.77 | 48.40 | 1 |
| 2243051 | *Streptopelia turtur* | Baden-Württemberg | 7.77 | 48.40 | 1 |
| 3888136 | *Streptopelia turtur* | Baden-Württemberg | 8.77 | 49.29 | 1 |
| 5332528 | *Streptopelia turtur* | Baden-Württemberg | 8.55 | 49.43 | 1 |
| 5337631 | *Streptopelia turtur* | Baden-Württemberg | 9.53 | 49.51 | 1 |
| 5339743 | *Streptopelia turtur* | Baden-Württemberg | 7.76 | 48.43 | 1 |
| 5350319 | *Streptopelia turtur* | Baden-Württemberg | 9.08 | 47.94 | 1 |
| 5385395 | *Streptopelia turtur* | Baden-Württemberg | 8.12 | 48.85 | 1 |
| 5390631 | *Streptopelia turtur* | Baden-Württemberg | 7.62 | 47.88 | 1 |
| 5397557 | *Streptopelia turtur* | Baden-Württemberg | 8.47 | 49.57 | 1 |
| 5397561 | *Streptopelia turtur* | Baden-Württemberg | 8.45 | 49.58 | 1 |
| 5437387 | *Streptopelia turtur* | Baden-Württemberg | 8.84 | 49.17 | 1 |
| 5441345 | *Streptopelia turtur* | Baden-Württemberg | 7.79 | 48.49 | 1 |

| **ID** | **species** | **Country** | **long** | **lat** | **PO** |
|---|---|---|---|---|---|
| 5442659 | *Streptopelia turtur* | Baden-Württemberg | 9.31 | 49.38 | 1 |
| 5448163 | *Streptopelia turtur* | Baden-Württemberg | 7.74 | 48.41 | 1 |
| 5454924 | *Streptopelia turtur* | Baden-Württemberg | 8.35 | 49.07 | 1 |
| 5469648 | *Streptopelia turtur* | Baden-Württemberg | 7.76 | 48.41 | 1 |
| 5499568 | *Streptopelia turtur* | Baden-Württemberg | 7.75 | 48.36 | 1 |
| 5516316 | *Streptopelia turtur* | Baden-Württemberg | 7.78 | 48.48 | 1 |
| 5555837 | *Streptopelia turtur* | Baden-Württemberg | 8.39 | 49.05 | 1 |
| 5584447 | *Streptopelia turtur* | Baden-Württemberg | 8.66 | 49.43 | 1 |
| 5604084 | *Streptopelia turtur* | Baden-Württemberg | 8.16 | 48.90 | 1 |
| 5605902 | *Streptopelia turtur* | Baden-Württemberg | 8.52 | 49.26 | 1 |
| 5607021 | *Streptopelia turtur* | Baden-Württemberg | 7.77 | 48.44 | 1 |
| 5631888 | *Streptopelia turtur* | Baden-Württemberg | 9.16 | 49.41 | 1 |
| 5633496 | *Streptopelia turtur* | Baden-Württemberg | 9.16 | 49.39 | 1 |
| 5653368 | *Streptopelia turtur* | Baden-Württemberg | 9.41 | 49.49 | 1 |
| 5661594 | *Streptopelia turtur* | Baden-Württemberg | 9.43 | 49.39 | 1 |
| 5667351 | *Streptopelia turtur* | Baden-Württemberg | 10.06 | 49.49 | 1 |
| 5691443 | *Streptopelia turtur* | Baden-Württemberg | 7.71 | 48.30 | 1 |
| 5939835 | *Streptopelia turtur* | Baden-Württemberg | 9.03 | 49.10 | 1 |

| ID | species | Country | long | lat | PO |
|---|---|---|---|---|---|
| 6732772 | *Streptopelia turtur* | Baden-Württemberg | 7.69 | 47.86 | 1 |
| 6732931 | *Streptopelia turtur* | Baden-Württemberg | 8.67 | 49.01 | 1 |
| 9496191 | *Streptopelia turtur* | Baden-Württemberg | 8.73 | 48.52 | 1 |
| 9532969 | *Streptopelia turtur* | Baden-Württemberg | 9.55 | 49.44 | 1 |
| 9540523 | *Streptopelia turtur* | Baden-Württemberg | 9.80 | 49.47 | 1 |
| 9551335 | *Streptopelia turtur* | Baden-Württemberg | 9.31 | 49.40 | 1 |
| 9565656 | *Streptopelia turtur* | Baden-Württemberg | 8.90 | 48.52 | 1 |
| 9566748 | *Streptopelia turtur* | Baden-Württemberg | 7.67 | 48.00 | 1 |
| 9566767 | *Streptopelia turtur* | Baden-Württemberg | 7.67 | 47.99 | 1 |
| 9566797 | *Streptopelia turtur* | Baden-Württemberg | 7.71 | 48.05 | 1 |
| 9567276 | *Streptopelia turtur* | Baden-Württemberg | 7.72 | 48.20 | 1 |
| 9592510 | *Streptopelia turtur* | Baden-Württemberg | 7.90 | 48.40 | 1 |
| 9598797 | *Streptopelia turtur* | Baden-Württemberg | 8.63 | 49.24 | 1 |
| 9605179 | *Streptopelia turtur* | Baden-Württemberg | 9.10 | 48.49 | 1 |
| 9634052 | *Streptopelia turtur* | Baden-Württemberg | 9.65 | 49.65 | 1 |
| 9634648 | *Streptopelia turtur* | Baden-Württemberg | 9.52 | 49.43 | 1 |
| 9639520 | *Streptopelia turtur* | Baden-Württemberg | 9.29 | 49.41 | 1 |
| 9640729 | *Streptopelia turtur* | Baden-Württemberg | 9.60 | 49.66 | 1 |

| ID | species | Country | long | lat | PO |
|---|---|---|---|---|---|
| 9663924 | *Streptopelia turtur* | Baden-Württemberg | 8.10 | 48.76 | 1 |
| 9706239 | *Streptopelia turtur* | Baden-Württemberg | 9.47 | 49.42 | 1 |
| 9714061 | *Streptopelia turtur* | Baden-Württemberg | 7.65 | 48.05 | 1 |
| 9714588 | *Streptopelia turtur* | Baden-Württemberg | 7.66 | 48.05 | 1 |
| 9716000 | *Streptopelia turtur* | Baden-Württemberg | 9.44 | 49.47 | 1 |
| 9725186 | *Streptopelia turtur* | Baden-Württemberg | 9.58 | 49.57 | 1 |
| 9726727 | *Streptopelia turtur* | Baden-Württemberg | 9.54 | 49.42 | 1 |
| 9733747 | *Streptopelia turtur* | Baden-Württemberg | 7.58 | 48.07 | 1 |
| 9733748 | *Streptopelia turtur* | Baden-Württemberg | 7.58 | 48.07 | 1 |
| 9738325 | *Streptopelia turtur* | Baden-Württemberg | 7.74 | 48.09 | 1 |
| 9745115 | *Streptopelia turtur* | Baden-Württemberg | 7.59 | 47.91 | 1 |
| 9756197 | *Streptopelia turtur* | Baden-Württemberg | 7.71 | 47.54 | 1 |
| 9768904 | *Streptopelia turtur* | Baden-Württemberg | 7.92 | 48.39 | 1 |
| 9770524 | *Streptopelia turtur* | Baden-Württemberg | 9.27 | 49.47 | 1 |
| 9779261 | *Streptopelia turtur* | Baden-Württemberg | 8.77 | 48.51 | 1 |
| 9813325 | *Streptopelia turtur* | Baden-Württemberg | 9.42 | 49.44 | 1 |
| 9815768 | *Streptopelia turtur* | Baden-Württemberg | 8.69 | 48.41 | 1 |
| 9815878 | *Streptopelia turtur* | Baden-Württemberg | 8.71 | 48.41 | 1 |

| **ID** | **species** | **Country** | **long** | **lat** | **PO** |
|---|---|---|---|---|---|
| 9829628 | *Streptopelia turtur* | Baden-Württemberg | 9.29 | 49.42 | 1 |
| 9836247 | *Streptopelia turtur* | Baden-Württemberg | 7.78 | 48.46 | 1 |
| 9860034 | *Streptopelia turtur* | Baden-Württemberg | 7.62 | 47.89 | 1 |
| 9885297 | *Streptopelia turtur* | Baden-Württemberg | 8.07 | 48.76 | 1 |
| 9885306 | *Streptopelia turtur* | Baden-Württemberg | 8.09 | 48.79 | 1 |
| 9943754 | *Streptopelia turtur* | Baden-Württemberg | 9.66 | 48.01 | 1 |
| 9989243 | *Streptopelia turtur* | Baden-Württemberg | 9.36 | 49.40 | 1 |
| 10000462 | *Streptopelia turtur* | Baden-Württemberg | 7.79 | 48.24 | 1 |
| 10031653 | *Streptopelia turtur* | Baden-Württemberg | 9.66 | 48.01 | 1 |
| 10041200 | *Streptopelia turtur* | Baden-Württemberg | 9.23 | 49.36 | 1 |
| 10054962 | *Streptopelia turtur* | Baden-Württemberg | 8.99 | 49.38 | 1 |
| 10079319 | *Streptopelia turtur* | Baden-Württemberg | 7.78 | 48.34 | 1 |
| 10099472 | *Streptopelia turtur* | Baden-Württemberg | 8.94 | 49.16 | 1 |
| 11064907 | *Streptopelia turtur* | Baden-Württemberg | 8.92 | 47.79 | 1 |
| 492975 | *Streptopelia turtur* | Bayern | 12.64 | 48.65 | 1 |
| 493149 | *Streptopelia turtur* | Bayern | 12.64 | 48.65 | 1 |
| 1754264 | *Streptopelia turtur* | Bayern | 10.40 | 49.50 | 1 |
| 1757488 | *Streptopelia turtur* | Bayern | 11.02 | 49.78 | 1 |

| ID | species | Country | long | lat | PO |
|---|---|---|---|---|---|
| 1780570 | *Streptopelia turtur* | Bayern | 10.20 | 50.41 | 1 |
| 1831901 | *Streptopelia turtur* | Bayern | 10.54 | 49.58 | 1 |
| 1835797 | *Streptopelia turtur* | Bayern | 10.75 | 49.65 | 1 |
| 1855645 | *Streptopelia turtur* | Bayern | 12.14 | 48.69 | 1 |
| 1863094 | *Streptopelia turtur* | Bayern | 10.33 | 49.51 | 1 |
| 1894590 | *Streptopelia turtur* | Bayern | 9.07 | 50.10 | 1 |
| 1921413 | *Streptopelia turtur* | Bayern | 9.13 | 49.93 | 1 |
| 1928823 | *Streptopelia turtur* | Bayern | 10.28 | 47.85 | 1 |
| 2003675 | *Streptopelia turtur* | Bayern | 10.30 | 49.97 | 1 |
| 2004256 | *Streptopelia turtur* | Bayern | 12.72 | 48.63 | 1 |
| 2009140 | *Streptopelia turtur* | Bayern | 9.10 | 50.09 | 1 |
| 2009347 | *Streptopelia turtur* | Bayern | 9.06 | 50.05 | 1 |
| 2034415 | *Streptopelia turtur* | Bayern | 9.09 | 50.09 | 1 |
| 2039114 | *Streptopelia turtur* | Bayern | 9.06 | 50.08 | 1 |
| 2056734 | *Streptopelia turtur* | Bayern | 9.09 | 50.09 | 1 |
| 2066629 | *Streptopelia turtur* | Bayern | 9.08 | 50.08 | 1 |
| 2077038 | *Streptopelia turtur* | Bayern | 10.38 | 50.32 | 1 |
| 2081373 | *Streptopelia turtur* | Bayern | 9.07 | 50.07 | 1 |

| **ID** | **species** | **Country** | **long** | **lat** | **PO** |
|---|---|---|---|---|---|
| 2082839 | *Streptopelia turtur* | Bayern | 10.18 | 50.35 | 1 |
| 2091332 | *Streptopelia turtur* | Bayern | 12.49 | 48.95 | 1 |
| 2191816 | *Streptopelia turtur* | Bayern | 9.05 | 50.06 | 1 |
| 2204214 | *Streptopelia turtur* | Bayern | 9.70 | 49.75 | 1 |
| 2216042 | *Streptopelia turtur* | Bayern | 9.83 | 49.84 | 1 |
| 2216224 | *Streptopelia turtur* | Bayern | 9.86 | 49.67 | 1 |
| 2233080 | *Streptopelia turtur* | Bayern | 9.11 | 50.11 | 1 |
| 2430124 | *Streptopelia turtur* | Bayern | 9.09 | 50.11 | 1 |
| 5310787 | *Streptopelia turtur* | Bayern | 10.83 | 49.78 | 1 |
| 5315201 | *Streptopelia turtur* | Bayern | 10.78 | 49.67 | 1 |
| 5320131 | *Streptopelia turtur* | Bayern | 12.54 | 49.90 | 1 |
| 5352944 | *Streptopelia turtur* | Bayern | 10.75 | 49.82 | 1 |
| 5360530 | *Streptopelia turtur* | Bayern | 10.20 | 49.96 | 1 |
| 5364753 | *Streptopelia turtur* | Bayern | 10.38 | 48.64 | 1 |
| 5365641 | *Streptopelia turtur* | Bayern | 12.30 | 49.90 | 1 |
| 5365643 | *Streptopelia turtur* | Bayern | 11.36 | 48.91 | 1 |
| 5372379 | *Streptopelia turtur* | Bayern | 10.78 | 49.83 | 1 |
| 5386297 | *Streptopelia turtur* | Bayern | 10.59 | 49.29 | 1 |

| **ID** | **species** | **Country** | **long** | **lat** | **PO** |
|---|---|---|---|---|---|
| 5409874 | *Streptopelia turtur* | Bayern | 12.18 | 48.66 | 1 |
| 5414601 | *Streptopelia turtur* | Bayern | 12.29 | 49.97 | 1 |
| 5426470 | *Streptopelia turtur* | Bayern | 11.09 | 48.53 | 1 |
| 5428902 | *Streptopelia turtur* | Bayern | 10.50 | 50.21 | 1 |
| 5428903 | *Streptopelia turtur* | Bayern | 10.52 | 50.21 | 1 |
| 5430633 | *Streptopelia turtur* | Bayern | 10.73 | 49.66 | 1 |
| 5433287 | *Streptopelia turtur* | Bayern | 10.87 | 49.98 | 1 |
| 5433551 | *Streptopelia turtur* | Bayern | 11.20 | 48.95 | 1 |
| 5444850 | *Streptopelia turtur* | Bayern | 9.82 | 49.92 | 1 |
| 5455010 | *Streptopelia turtur* | Bayern | 12.25 | 50.05 | 1 |
| 5481814 | *Streptopelia turtur* | Bayern | 10.71 | 49.78 | 1 |
| 5496725 | *Streptopelia turtur* | Bayern | 12.13 | 48.69 | 1 |
| 5523645 | *Streptopelia turtur* | Bayern | 12.78 | 48.70 | 1 |
| 5527793 | *Streptopelia turtur* | Bayern | 10.86 | 49.77 | 1 |
| 5577367 | *Streptopelia turtur* | Bayern | 10.34 | 49.67 | 1 |
| 5631858 | *Streptopelia turtur* | Bayern | 12.63 | 48.66 | 1 |
| 5648200 | *Streptopelia turtur* | Bayern | 10.92 | 49.74 | 1 |
| 5686753 | *Streptopelia turtur* | Bayern | 10.93 | 49.74 | 1 |

| **ID** | **species** | **Country** | **long** | **lat** | **PO** |
|---|---|---|---|---|---|
| 5711368 | *Streptopelia turtur* | Bayern | 12.14 | 48.63 | 1 |
| 5726296 | *Streptopelia turtur* | Bayern | 10.44 | 49.45 | 1 |
| 5738791 | *Streptopelia turtur* | Bayern | 12.75 | 48.69 | 1 |
| 5749204 | *Streptopelia turtur* | Bayern | 12.28 | 49.99 | 1 |
| 9493846 | *Streptopelia turtur* | Bayern | 10.90 | 48.20 | 1 |
| 9515728 | *Streptopelia turtur* | Bayern | 9.79 | 49.98 | 1 |
| 9542428 | *Streptopelia turtur* | Bayern | 9.79 | 49.83 | 1 |
| 9548175 | *Streptopelia turtur* | Bayern | 12.68 | 48.64 | 1 |
| 9561416 | *Streptopelia turtur* | Bayern | 12.32 | 49.85 | 1 |
| 9572524 | *Streptopelia turtur* | Bayern | 10.27 | 50.40 | 1 |
| 9574926 | *Streptopelia turtur* | Bayern | 12.95 | 48.23 | 1 |
| 9591693 | *Streptopelia turtur* | Bayern | 12.18 | 48.64 | 1 |
| 9603509 | *Streptopelia turtur* | Bayern | 11.21 | 49.16 | 1 |
| 9605283 | *Streptopelia turtur* | Bayern | 12.20 | 50.16 | 1 |
| 9657246 | *Streptopelia turtur* | Bayern | 12.17 | 48.65 | 1 |
| 9657253 | *Streptopelia turtur* | Bayern | 12.15 | 48.65 | 1 |
| 9667866 | *Streptopelia turtur* | Bayern | 10.29 | 49.88 | 1 |
| 9679639 | *Streptopelia turtur* | Bayern | 10.24 | 49.87 | 1 |

| **ID** | **species** | **Country** | **long** | **lat** | **PO** |
|---|---|---|---|---|---|
| 9687019 | *Streptopelia turtur* | Bayern | 10.59 | 50.32 | 1 |
| 9728241 | *Streptopelia turtur* | Bayern | 10.76 | 49.82 | 1 |
| 9751546 | *Streptopelia turtur* | Bayern | 10.23 | 50.21 | 1 |
| 9760349 | *Streptopelia turtur* | Bayern | 10.94 | 50.04 | 1 |
| 9788654 | *Streptopelia turtur* | Bayern | 10.25 | 49.88 | 1 |
| 9800679 | *Streptopelia turtur* | Bayern | 10.57 | 49.08 | 1 |
| 9812911 | *Streptopelia turtur* | Bayern | 10.21 | 49.68 | 1 |
| 9832484 | *Streptopelia turtur* | Bayern | 11.91 | 48.31 | 1 |
| 9866542 | *Streptopelia turtur* | Bayern | 10.09 | 49.69 | 1 |
| 9917602 | *Streptopelia turtur* | Bayern | 12.28 | 49.84 | 1 |
| 9928572 | *Streptopelia turtur* | Bayern | 10.44 | 48.75 | 1 |
| 9957638 | *Streptopelia turtur* | Bayern | 12.29 | 50.04 | 1 |
| 9967547 | *Streptopelia turtur* | Bayern | 11.19 | 49.17 | 1 |
| 9967784 | *Streptopelia turtur* | Bayern | 10.17 | 49.97 | 1 |
| 10083130 | *Streptopelia turtur* | Bayern | 10.22 | 49.81 | 1 |
| 397437 | *Streptopelia turtur* | Brandenburg | 13.03 | 52.08 | 1 |
| 574938 | *Streptopelia turtur* | Brandenburg | 14.44 | 51.57 | 1 |
| 574956 | *Streptopelia turtur* | Brandenburg | 14.44 | 51.56 | 1 |

| **ID** | **species** | **Country** | **long** | **lat** | **PO** |
|---|---|---|---|---|---|
| 1422600 | *Streptopelia turtur* | Brandenburg | 14.29 | 51.63 | 1 |
| 1724396 | *Streptopelia turtur* | Brandenburg | 12.90 | 52.00 | 1 |
| 1790846 | *Streptopelia turtur* | Brandenburg | 14.21 | 52.52 | 1 |
| 1793208 | *Streptopelia turtur* | Brandenburg | 14.07 | 52.84 | 1 |
| 1803748 | *Streptopelia turtur* | Brandenburg | 14.14 | 53.21 | 1 |
| 1820347 | *Streptopelia turtur* | Brandenburg | 14.50 | 52.03 | 1 |
| 1850814 | *Streptopelia turtur* | Brandenburg | 14.10 | 53.22 | 1 |
| 1934604 | *Streptopelia turtur* | Brandenburg | 12.54 | 52.27 | 1 |
| 1944330 | *Streptopelia turtur* | Brandenburg | 13.25 | 51.42 | 1 |
| 1949973 | *Streptopelia turtur* | Brandenburg | 14.06 | 51.55 | 1 |
| 1962479 | *Streptopelia turtur* | Brandenburg | 12.99 | 51.95 | 1 |
| 1968631 | *Streptopelia turtur* | Brandenburg | 13.27 | 51.43 | 1 |
| 1972268 | *Streptopelia turtur* | Brandenburg | 14.59 | 52.60 | 1 |
| 1989827 | *Streptopelia turtur* | Brandenburg | 14.61 | 52.48 | 1 |
| 2001539 | *Streptopelia turtur* | Brandenburg | 13.01 | 51.94 | 1 |
| 2047658 | *Streptopelia turtur* | Brandenburg | 13.12 | 51.92 | 1 |
| 2092858 | *Streptopelia turtur* | Brandenburg | 13.27 | 51.49 | 1 |
| 2093299 | *Streptopelia turtur* | Brandenburg | 13.64 | 51.40 | 1 |

| **ID** | **species** | **Country** | **long** | **lat** | **PO** |
|---|---|---|---|---|---|
| 2101732 | *Streptopelia turtur* | Brandenburg | 13.68 | 51.47 | 1 |
| 2118572 | *Streptopelia turtur* | Brandenburg | 14.08 | 51.55 | 1 |
| 2153957 | *Streptopelia turtur* | Brandenburg | 12.69 | 52.53 | 1 |
| 3801239 | *Streptopelia turtur* | Brandenburg | 14.42 | 51.66 | 1 |
| 3801642 | *Streptopelia turtur* | Brandenburg | 14.46 | 51.64 | 1 |
| 4150905 | *Streptopelia turtur* | Brandenburg | 13.09 | 52.20 | 1 |
| 5277553 | *Streptopelia turtur* | Brandenburg | 14.16 | 53.01 | 1 |
| 5299227 | *Streptopelia turtur* | Brandenburg | 14.13 | 52.44 | 1 |
| 5305124 | *Streptopelia turtur* | Brandenburg | 14.12 | 53.05 | 1 |
| 5306259 | *Streptopelia turtur* | Brandenburg | 14.31 | 53.25 | 1 |
| 5348806 | *Streptopelia turtur* | Brandenburg | 14.17 | 53.18 | 1 |
| 5388465 | *Streptopelia turtur* | Brandenburg | 13.36 | 51.49 | 1 |
| 5388499 | *Streptopelia turtur* | Brandenburg | 13.30 | 51.47 | 1 |
| 5390618 | *Streptopelia turtur* | Brandenburg | 12.30 | 52.32 | 1 |
| 5393067 | *Streptopelia turtur* | Brandenburg | 12.72 | 52.54 | 1 |
| 5405312 | *Streptopelia turtur* | Brandenburg | 13.60 | 51.41 | 1 |
| 5405324 | *Streptopelia turtur* | Brandenburg | 13.58 | 51.42 | 1 |
| 5407336 | *Streptopelia turtur* | Brandenburg | 14.17 | 53.15 | 1 |

| **ID** | **species** | **Country** | **long** | **lat** | **PO** |
|---|---|---|---|---|---|
| 5422217 | *Streptopelia turtur* | Brandenburg | 14.17 | 53.21 | 1 |
| 5422244 | *Streptopelia turtur* | Brandenburg | 14.17 | 53.21 | 1 |
| 5422632 | *Streptopelia turtur* | Brandenburg | 14.14 | 53.22 | 1 |
| 5426434 | *Streptopelia turtur* | Brandenburg | 13.12 | 52.19 | 1 |
| 5472728 | *Streptopelia turtur* | Brandenburg | 13.08 | 52.05 | 1 |
| 5478732 | *Streptopelia turtur* | Brandenburg | 14.20 | 51.55 | 1 |
| 5483504 | *Streptopelia turtur* | Brandenburg | 14.13 | 53.15 | 1 |
| 5490450 | *Streptopelia turtur* | Brandenburg | 13.88 | 53.26 | 1 |
| 5538578 | *Streptopelia turtur* | Brandenburg | 14.13 | 53.24 | 1 |
| 5539413 | *Streptopelia turtur* | Brandenburg | 14.16 | 53.23 | 1 |
| 5542257 | *Streptopelia turtur* | Brandenburg | 12.15 | 52.92 | 1 |
| 5558481 | *Streptopelia turtur* | Brandenburg | 14.72 | 52.08 | 1 |
| 5559428 | *Streptopelia turtur* | Brandenburg | 14.10 | 51.53 | 1 |
| 5559467 | *Streptopelia turtur* | Brandenburg | 14.11 | 51.52 | 1 |
| 5561331 | *Streptopelia turtur* | Brandenburg | 14.13 | 53.04 | 1 |
| 5576184 | *Streptopelia turtur* | Brandenburg | 13.90 | 53.08 | 1 |
| 5577404 | *Streptopelia turtur* | Brandenburg | 12.67 | 52.23 | 1 |
| 5611888 | *Streptopelia turtur* | Brandenburg | 14.13 | 51.57 | 1 |

| ID | species | Country | long | lat | PO |
|---|---|---|---|---|---|
| 5611900 | *Streptopelia turtur* | Brandenburg | 14.15 | 51.57 | 1 |
| 5612790 | *Streptopelia turtur* | Brandenburg | 14.24 | 53.23 | 1 |
| 5613237 | *Streptopelia turtur* | Brandenburg | 14.14 | 53.04 | 1 |
| 5657818 | *Streptopelia turtur* | Brandenburg | 14.47 | 51.68 | 1 |
| 5673234 | *Streptopelia turtur* | Brandenburg | 14.25 | 53.19 | 1 |
| 5674184 | *Streptopelia turtur* | Brandenburg | 14.13 | 53.23 | 1 |
| 5684565 | *Streptopelia turtur* | Brandenburg | 12.99 | 52.20 | 1 |
| 5695775 | *Streptopelia turtur* | Brandenburg | 14.68 | 52.20 | 1 |
| 5704230 | *Streptopelia turtur* | Brandenburg | 13.21 | 51.42 | 1 |
| 5710287 | *Streptopelia turtur* | Brandenburg | 14.41 | 51.59 | 1 |
| 5736587 | *Streptopelia turtur* | Brandenburg | 13.35 | 51.96 | 1 |
| 5752382 | *Streptopelia turtur* | Brandenburg | 13.00 | 52.18 | 1 |
| 5754610 | *Streptopelia turtur* | Brandenburg | 14.44 | 51.65 | 1 |
| 6336662 | *Streptopelia turtur* | Brandenburg | 14.31 | 51.64 | 1 |
| 6337049 | *Streptopelia turtur* | Brandenburg | 14.34 | 51.62 | 1 |
| 7082708 | *Streptopelia turtur* | Brandenburg | 13.74 | 51.78 | 1 |
| 7551357 | *Streptopelia turtur* | Brandenburg | 13.28 | 52.06 | 1 |
| 9034639 | *Streptopelia turtur* | Brandenburg | 12.89 | 52.61 | 1 |

| ID | species | Country | long | lat | PO |
|---|---|---|---|---|---|
| 9532897 | *Streptopelia turtur* | Brandenburg | 12.82 | 52.01 | 1 |
| 9544110 | *Streptopelia turtur* | Brandenburg | 14.22 | 51.55 | 1 |
| 9547705 | *Streptopelia turtur* | Brandenburg | 13.29 | 52.03 | 1 |
| 9550329 | *Streptopelia turtur* | Brandenburg | 13.41 | 52.96 | 1 |
| 9565498 | *Streptopelia turtur* | Brandenburg | 13.91 | 51.81 | 1 |
| 9566454 | *Streptopelia turtur* | Brandenburg | 13.25 | 52.03 | 1 |
| 9569445 | *Streptopelia turtur* | Brandenburg | 14.04 | 53.10 | 1 |
| 9583908 | *Streptopelia turtur* | Brandenburg | 14.17 | 53.21 | 1 |
| 9591204 | *Streptopelia turtur* | Brandenburg | 14.22 | 52.19 | 1 |
| 9595426 | *Streptopelia turtur* | Brandenburg | 14.13 | 51.56 | 1 |
| 9607875 | *Streptopelia turtur* | Brandenburg | 14.15 | 53.05 | 1 |
| 9614353 | *Streptopelia turtur* | Brandenburg | 13.52 | 51.79 | 1 |
| 9618618 | *Streptopelia turtur* | Brandenburg | 12.97 | 52.18 | 1 |
| 9618900 | *Streptopelia turtur* | Brandenburg | 12.97 | 52.17 | 1 |
| 9624655 | *Streptopelia turtur* | Brandenburg | 14.32 | 51.59 | 1 |
| 9629735 | *Streptopelia turtur* | Brandenburg | 14.30 | 51.61 | 1 |
| 9634440 | *Streptopelia turtur* | Brandenburg | 14.25 | 51.68 | 1 |
| 9636259 | *Streptopelia turtur* | Brandenburg | 13.08 | 52.23 | 1 |

| **ID** | **species** | **Country** | **long** | **lat** | **PO** |
| --- | --- | --- | --- | --- | --- |
| 9665577 | *Streptopelia turtur* | Brandenburg | 13.70 | 51.54 | 1 |
| 9672023 | *Streptopelia turtur* | Brandenburg | 13.30 | 52.02 | 1 |
| 9672030 | *Streptopelia turtur* | Brandenburg | 13.23 | 52.02 | 1 |
| 9678482 | *Streptopelia turtur* | Brandenburg | 13.54 | 51.79 | 1 |
| 9694369 | *Streptopelia turtur* | Brandenburg | 13.26 | 52.04 | 1 |
| 9742027 | *Streptopelia turtur* | Brandenburg | 13.46 | 51.64 | 1 |
| 9762207 | *Streptopelia turtur* | Brandenburg | 13.79 | 51.66 | 1 |
| 9762219 | *Streptopelia turtur* | Brandenburg | 13.77 | 51.58 | 1 |
| 9771669 | *Streptopelia turtur* | Brandenburg | 14.23 | 51.55 | 1 |
| 9778867 | *Streptopelia turtur* | Brandenburg | 13.53 | 51.55 | 1 |
| 9808680 | *Streptopelia turtur* | Brandenburg | 14.47 | 51.76 | 1 |
| 9808918 | *Streptopelia turtur* | Brandenburg | 13.55 | 51.77 | 1 |
| 9871343 | *Streptopelia turtur* | Brandenburg | 14.17 | 53.21 | 1 |
| 9872641 | *Streptopelia turtur* | Brandenburg | 13.11 | 52.07 | 1 |
| 9872708 | *Streptopelia turtur* | Brandenburg | 13.25 | 52.04 | 1 |
| 9884668 | *Streptopelia turtur* | Brandenburg | 13.83 | 51.67 | 1 |
| 9900575 | *Streptopelia turtur* | Brandenburg | 13.84 | 52.04 | 1 |
| 9908080 | *Streptopelia turtur* | Brandenburg | 13.57 | 51.78 | 1 |

| **ID** | **species** | **Country** | **long** | **lat** | **PO** |
|---|---|---|---|---|---|
| 9914448 | *Streptopelia turtur* | Brandenburg | 14.56 | 51.89 | 1 |
| 9925193 | *Streptopelia turtur* | Brandenburg | 14.34 | 51.61 | 1 |
| 9931326 | *Streptopelia turtur* | Brandenburg | 13.05 | 52.06 | 1 |
| 9957014 | *Streptopelia turtur* | Brandenburg | 13.72 | 51.57 | 1 |
| 9957057 | *Streptopelia turtur* | Brandenburg | 13.89 | 51.82 | 1 |
| 9957440 | *Streptopelia turtur* | Brandenburg | 13.70 | 51.54 | 1 |
| 9960010 | *Streptopelia turtur* | Brandenburg | 13.82 | 51.98 | 1 |
| 9966494 | *Streptopelia turtur* | Brandenburg | 14.45 | 51.59 | 1 |
| 9966628 | *Streptopelia turtur* | Brandenburg | 14.25 | 51.53 | 1 |
| 9976637 | *Streptopelia turtur* | Brandenburg | 14.16 | 53.20 | 1 |
| 9991336 | *Streptopelia turtur* | Brandenburg | 14.04 | 51.98 | 1 |
| 10054068 | *Streptopelia turtur* | Brandenburg | 13.92 | 51.76 | 1 |
| 10065073 | *Streptopelia turtur* | Brandenburg | 14.09 | 51.58 | 1 |
| 10068784 | *Streptopelia turtur* | Brandenburg | 13.84 | 51.84 | 1 |
| 10094083 | *Streptopelia turtur* | Brandenburg | 14.30 | 51.57 | 1 |
| 10185161 | *Streptopelia turtur* | Brandenburg | 13.03 | 51.93 | 1 |
| 10185188 | *Streptopelia turtur* | Brandenburg | 12.92 | 52.01 | 1 |
| 10696293 | *Streptopelia turtur* | Brandenburg | 14.31 | 52.51 | 1 |

| **ID** | **species** | **Country** | **long** | **lat** | **PO** |
|---|---|---|---|---|---|
| 10708492 | *Streptopelia turtur* | Brandenburg | 14.30 | 52.54 | 1 |
| 10709137 | *Streptopelia turtur* | Brandenburg | 14.28 | 52.54 | 1 |
| 1708621 | *Streptopelia turtur* | Hessen | 10.19 | 51.13 | 1 |
| 1711918 | *Streptopelia turtur* | Hessen | 9.16 | 50.45 | 1 |
| 1720267 | *Streptopelia turtur* | Hessen | 9.22 | 50.47 | 1 |
| 1737665 | *Streptopelia turtur* | Hessen | 9.00 | 49.88 | 1 |
| 1767013 | *Streptopelia turtur* | Hessen | 9.35 | 51.11 | 1 |
| 1771280 | *Streptopelia turtur* | Hessen | 8.75 | 51.18 | 1 |
| 1773085 | *Streptopelia turtur* | Hessen | 9.32 | 51.54 | 1 |
| 1798621 | *Streptopelia turtur* | Hessen | 8.77 | 50.28 | 1 |
| 1810854 | *Streptopelia turtur* | Hessen | 8.68 | 50.50 | 1 |
| 1831218 | *Streptopelia turtur* | Hessen | 8.69 | 50.69 | 1 |
| 1858639 | *Streptopelia turtur* | Hessen | 8.63 | 50.60 | 1 |
| 1868535 | *Streptopelia turtur* | Hessen | 8.87 | 49.80 | 1 |
| 1875157 | *Streptopelia turtur* | Hessen | 10.14 | 51.15 | 1 |
| 1889905 | *Streptopelia turtur* | Hessen | 10.16 | 51.15 | 1 |
| 1895059 | *Streptopelia turtur* | Hessen | 10.18 | 51.14 | 1 |
| 1908970 | *Streptopelia turtur* | Hessen | 10.05 | 51.16 | 1 |

| ID | species | Country | long | lat | PO |
|---|---|---|---|---|---|
| 1917099 | *Streptopelia turtur* | Hessen | 9.62 | 51.58 | 1 |
| 1940559 | *Streptopelia turtur* | Hessen | 9.72 | 51.10 | 1 |
| 1969727 | *Streptopelia turtur* | Hessen | 8.69 | 50.67 | 1 |
| 2000487 | *Streptopelia turtur* | Hessen | 8.96 | 50.66 | 1 |
| 2057774 | *Streptopelia turtur* | Hessen | 8.57 | 49.68 | 1 |
| 2100409 | *Streptopelia turtur* | Hessen | 8.66 | 50.94 | 1 |
| 2166024 | *Streptopelia turtur* | Hessen | 8.84 | 49.78 | 1 |
| 2258102 | *Streptopelia turtur* | Hessen | 9.10 | 50.12 | 1 |
| 2780673 | *Streptopelia turtur* | Hessen | 9.32 | 51.12 | 1 |
| 3260380 | *Streptopelia turtur* | Hessen | 9.09 | 50.96 | 1 |
| 3482235 | *Streptopelia turtur* | Hessen | 9.17 | 50.84 | 1 |
| 3601037 | *Streptopelia turtur* | Hessen | 9.18 | 50.84 | 1 |
| 3601063 | *Streptopelia turtur* | Hessen | 9.23 | 51.00 | 1 |
| 3601065 | *Streptopelia turtur* | Hessen | 9.38 | 51.13 | 1 |
| 3750608 | *Streptopelia turtur* | Hessen | 8.95 | 49.77 | 1 |
| 5285215 | *Streptopelia turtur* | Hessen | 7.99 | 50.01 | 1 |
| 5314223 | *Streptopelia turtur* | Hessen | 8.55 | 50.44 | 1 |
| 5327742 | *Streptopelia turtur* | Hessen | 9.13 | 51.19 | 1 |

| **ID** | **species** | **Country** | **long** | **lat** | **PO** |
|---|---|---|---|---|---|
| 5342159 | *Streptopelia turtur* | Hessen | 8.58 | 50.68 | 1 |
| 5349851 | *Streptopelia turtur* | Hessen | 8.83 | 49.80 | 1 |
| 5357411 | *Streptopelia turtur* | Hessen | 8.65 | 50.35 | 1 |
| 5357436 | *Streptopelia turtur* | Hessen | 8.95 | 50.45 | 1 |
| 5357443 | *Streptopelia turtur* | Hessen | 8.96 | 50.45 | 1 |
| 5380715 | *Streptopelia turtur* | Hessen | 10.16 | 51.19 | 1 |
| 5388892 | *Streptopelia turtur* | Hessen | 8.46 | 50.38 | 1 |
| 5397430 | *Streptopelia turtur* | Hessen | 8.62 | 50.38 | 1 |
| 5406498 | *Streptopelia turtur* | Hessen | 8.57 | 50.12 | 1 |
| 5424923 | *Streptopelia turtur* | Hessen | 8.59 | 50.70 | 1 |
| 5443209 | *Streptopelia turtur* | Hessen | 9.13 | 50.36 | 1 |
| 5465002 | *Streptopelia turtur* | Hessen | 9.31 | 50.41 | 1 |
| 5474173 | *Streptopelia turtur* | Hessen | 8.86 | 50.98 | 1 |
| 5477732 | *Streptopelia turtur* | Hessen | 8.69 | 50.88 | 1 |
| 5482631 | *Streptopelia turtur* | Hessen | 8.80 | 50.40 | 1 |
| 5487809 | *Streptopelia turtur* | Hessen | 9.28 | 50.95 | 1 |
| 5489334 | *Streptopelia turtur* | Hessen | 8.95 | 50.54 | 1 |
| 5494808 | *Streptopelia turtur* | Hessen | 8.68 | 50.70 | 1 |

| ID | species | Country | long | lat | PO |
|---|---|---|---|---|---|
| 5503150 | *Streptopelia turtur* | Hessen | 9.11 | 51.19 | 1 |
| 5503159 | *Streptopelia turtur* | Hessen | 9.11 | 51.18 | 1 |
| 5503166 | *Streptopelia turtur* | Hessen | 9.14 | 51.18 | 1 |
| 5503183 | *Streptopelia turtur* | Hessen | 9.10 | 51.17 | 1 |
| 5503188 | *Streptopelia turtur* | Hessen | 9.13 | 51.17 | 1 |
| 5503191 | *Streptopelia turtur* | Hessen | 9.14 | 51.16 | 1 |
| 5503194 | *Streptopelia turtur* | Hessen | 9.17 | 51.17 | 1 |
| 5523897 | *Streptopelia turtur* | Hessen | 8.39 | 50.01 | 1 |
| 5560786 | *Streptopelia turtur* | Hessen | 8.70 | 50.89 | 1 |
| 5560801 | *Streptopelia turtur* | Hessen | 8.68 | 50.90 | 1 |
| 5565154 | *Streptopelia turtur* | Hessen | 9.01 | 49.97 | 1 |
| 5574669 | *Streptopelia turtur* | Hessen | 8.95 | 50.41 | 1 |
| 5583558 | *Streptopelia turtur* | Hessen | 8.39 | 50.85 | 1 |
| 5583871 | *Streptopelia turtur* | Hessen | 8.93 | 50.51 | 1 |
| 5584932 | *Streptopelia turtur* | Hessen | 8.61 | 50.40 | 1 |
| 5603699 | *Streptopelia turtur* | Hessen | 8.87 | 51.00 | 1 |
| 5620294 | *Streptopelia turtur* | Hessen | 9.21 | 50.48 | 1 |
| 5628726 | *Streptopelia turtur* | Hessen | 8.69 | 50.92 | 1 |

| **ID** | **species** | **Country** | **long** | **lat** | **PO** |
| --- | --- | --- | --- | --- | --- |
| 5635533 | *Streptopelia turtur* | Hessen | 8.92 | 50.85 | 1 |
| 5641396 | *Streptopelia turtur* | Hessen | 8.63 | 50.90 | 1 |
| 5658340 | *Streptopelia turtur* | Hessen | 9.21 | 51.46 | 1 |
| 5693224 | *Streptopelia turtur* | Hessen | 8.03 | 50.41 | 1 |
| 5794086 | *Streptopelia turtur* | Hessen | 8.86 | 49.79 | 1 |
| 7381048 | *Streptopelia turtur* | Hessen | 9.28 | 50.81 | 1 |
| 9485555 | *Streptopelia turtur* | Hessen | 8.89 | 50.97 | 1 |
| 9486935 | *Streptopelia turtur* | Hessen | 9.16 | 50.86 | 1 |
| 9498358 | *Streptopelia turtur* | Hessen | 9.28 | 50.95 | 1 |
| 9506193 | *Streptopelia turtur* | Hessen | 8.04 | 50.03 | 1 |
| 9547867 | *Streptopelia turtur* | Hessen | 9.34 | 50.63 | 1 |
| 9550269 | *Streptopelia turtur* | Hessen | 9.44 | 51.15 | 1 |
| 9551057 | *Streptopelia turtur* | Hessen | 8.76 | 51.15 | 1 |
| 9551081 | *Streptopelia turtur* | Hessen | 8.76 | 51.17 | 1 |
| 9551243 | *Streptopelia turtur* | Hessen | 8.83 | 51.06 | 1 |
| 9557731 | *Streptopelia turtur* | Hessen | 8.36 | 50.39 | 1 |
| 9568519 | *Streptopelia turtur* | Hessen | 8.95 | 50.76 | 1 |
| 9574500 | *Streptopelia turtur* | Hessen | 9.61 | 50.54 | 1 |

| **ID** | **species** | **Country** | **long** | **lat** | **PO** |
|---|---|---|---|---|---|
| 9574511 | *Streptopelia turtur* | Hessen | 9.57 | 50.53 | 1 |
| 9584310 | *Streptopelia turtur* | Hessen | 9.13 | 51.19 | 1 |
| 9587020 | *Streptopelia turtur* | Hessen | 8.43 | 49.61 | 1 |
| 9592054 | *Streptopelia turtur* | Hessen | 9.17 | 50.72 | 1 |
| 9603631 | *Streptopelia turtur* | Hessen | 9.02 | 49.89 | 1 |
| 9605449 | *Streptopelia turtur* | Hessen | 9.78 | 51.12 | 1 |
| 9612192 | *Streptopelia turtur* | Hessen | 8.00 | 50.46 | 1 |
| 9616337 | *Streptopelia turtur* | Hessen | 9.20 | 50.83 | 1 |
| 9617138 | *Streptopelia turtur* | Hessen | 8.86 | 50.97 | 1 |
| 9630984 | *Streptopelia turtur* | Hessen | 8.94 | 50.53 | 1 |
| 9630999 | *Streptopelia turtur* | Hessen | 8.95 | 50.52 | 1 |
| 9640240 | *Streptopelia turtur* | Hessen | 9.14 | 50.44 | 1 |
| 9653210 | *Streptopelia turtur* | Hessen | 8.88 | 50.50 | 1 |
| 9663947 | *Streptopelia turtur* | Hessen | 9.53 | 51.54 | 1 |
| 9676918 | *Streptopelia turtur* | Hessen | 8.85 | 51.07 | 1 |
| 9682585 | *Streptopelia turtur* | Hessen | 8.99 | 49.96 | 1 |
| 9685757 | *Streptopelia turtur* | Hessen | 8.92 | 50.45 | 1 |
| 9692773 | *Streptopelia turtur* | Hessen | 8.61 | 49.78 | 1 |

| **ID** | **species** | **Country** | **long** | **lat** | **PO** |
|---|---|---|---|---|---|
| 9711706 | *Streptopelia turtur* | Hessen | 8.75 | 49.93 | 1 |
| 9722248 | *Streptopelia turtur* | Hessen | 9.31 | 50.93 | 1 |
| 9727269 | *Streptopelia turtur* | Hessen | 8.73 | 51.15 | 1 |
| 9727328 | *Streptopelia turtur* | Hessen | 8.73 | 51.14 | 1 |
| 9727502 | *Streptopelia turtur* | Hessen | 8.80 | 51.21 | 1 |
| 9733164 | *Streptopelia turtur* | Hessen | 9.31 | 50.93 | 1 |
| 9739123 | *Streptopelia turtur* | Hessen | 8.85 | 51.05 | 1 |
| 9744417 | *Streptopelia turtur* | Hessen | 8.95 | 49.94 | 1 |
| 9744676 | *Streptopelia turtur* | Hessen | 8.67 | 50.90 | 1 |
| 9762812 | *Streptopelia turtur* | Hessen | 8.66 | 50.85 | 1 |
| 9784053 | *Streptopelia turtur* | Hessen | 8.64 | 50.78 | 1 |
| 9784059 | *Streptopelia turtur* | Hessen | 9.22 | 51.28 | 1 |
| 9802520 | *Streptopelia turtur* | Hessen | 9.85 | 50.62 | 1 |
| 9812645 | *Streptopelia turtur* | Hessen | 10.13 | 51.19 | 1 |
| 9829387 | *Streptopelia turtur* | Hessen | 8.64 | 50.34 | 1 |
| 9831356 | *Streptopelia turtur* | Hessen | 8.72 | 50.91 | 1 |
| 9840294 | *Streptopelia turtur* | Hessen | 9.16 | 50.84 | 1 |
| 9840501 | *Streptopelia turtur* | Hessen | 9.22 | 50.82 | 1 |

| **ID** | **species** | **Country** | **long** | **lat** | **PO** |
|---|---|---|---|---|---|
| 9882820 | *Streptopelia turtur* | Hessen | 9.64 | 50.52 | 1 |
| 9908794 | *Streptopelia turtur* | Hessen | 8.34 | 50.03 | 1 |
| 9915387 | *Streptopelia turtur* | Hessen | 8.92 | 50.45 | 1 |
| 9941744 | *Streptopelia turtur* | Hessen | 9.26 | 51.10 | 1 |
| 9946105 | *Streptopelia turtur* | Hessen | 8.92 | 49.94 | 1 |
| 9971083 | *Streptopelia turtur* | Hessen | 8.13 | 50.08 | 1 |
| 9980307 | *Streptopelia turtur* | Hessen | 8.13 | 50.20 | 1 |
| 9981331 | *Streptopelia turtur* | Hessen | 10.20 | 51.15 | 1 |
| 9984583 | *Streptopelia turtur* | Hessen | 8.45 | 50.05 | 1 |
| 9995012 | *Streptopelia turtur* | Hessen | 8.94 | 50.33 | 1 |
| 9999700 | *Streptopelia turtur* | Hessen | 10.20 | 51.15 | 1 |
| 10013025 | *Streptopelia turtur* | Hessen | 8.84 | 51.26 | 1 |
| 10015251 | *Streptopelia turtur* | Hessen | 8.45 | 50.10 | 1 |
| 10047193 | *Streptopelia turtur* | Hessen | 8.46 | 50.05 | 1 |
| 10047917 | *Streptopelia turtur* | Hessen | 8.54 | 49.90 | 1 |
| 10052440 | *Streptopelia turtur* | Hessen | 8.96 | 50.47 | 1 |
| 10065200 | *Streptopelia turtur* | Hessen | 8.87 | 50.96 | 1 |
| 10077484 | *Streptopelia turtur* | Hessen | 9.30 | 50.90 | 1 |

| ID | species | Country | long | lat | PO |
|---|---|---|---|---|---|
| 10094364 | *Streptopelia turtur* | Hessen | 8.37 | 49.87 | 1 |
| 10116365 | *Streptopelia turtur* | Hessen | 9.49 | 51.47 | 1 |
| 10139281 | *Streptopelia turtur* | Hessen | 9.22 | 50.38 | 1 |
| 10229946 | *Streptopelia turtur* | Hessen | 8.74 | 51.07 | 1 |
| 820168 | *Streptopelia turtur* | Mecklenburg-Vorpommern | 11.87 | 54.02 | 1 |
| 1686738 | *Streptopelia turtur* | Mecklenburg-Vorpommern | 10.82 | 53.72 | 1 |
| 1686741 | *Streptopelia turtur* | Mecklenburg-Vorpommern | 10.82 | 53.72 | 1 |
| 1722365 | *Streptopelia turtur* | Mecklenburg-Vorpommern | 10.99 | 53.48 | 1 |
| 1875158 | *Streptopelia turtur* | Mecklenburg-Vorpommern | 10.77 | 53.81 | 1 |
| 1936818 | *Streptopelia turtur* | Mecklenburg-Vorpommern | 11.07 | 53.29 | 1 |
| 1949017 | *Streptopelia turtur* | Mecklenburg-Vorpommern | 11.22 | 53.32 | 1 |
| 1957118 | *Streptopelia turtur* | Mecklenburg-Vorpommern | 13.12 | 54.60 | 1 |
| 2099715 | *Streptopelia turtur* | Mecklenburg-Vorpommern | 11.33 | 53.51 | 1 |
| 2728592 | *Streptopelia turtur* | Mecklenburg-Vorpommern | 13.36 | 54.05 | 1 |
| 4122163 | *Streptopelia turtur* | Mecklenburg-Vorpommern | 12.89 | 54.14 | 1 |
| 4122216 | *Streptopelia turtur* | Mecklenburg-Vorpommern | 12.84 | 54.12 | 1 |
| 4122231 | *Streptopelia turtur* | Mecklenburg-Vorpommern | 12.93 | 54.14 | 1 |
| 4122238 | *Streptopelia turtur* | Mecklenburg-Vorpommern | 12.91 | 54.15 | 1 |

| ID | species | Country | long | lat | PO |
|---|---|---|---|---|---|
| 4122365 | *Streptopelia turtur* | Mecklenburg-Vorpommern | 12.83 | 54.31 | 1 |
| 4122575 | *Streptopelia turtur* | Mecklenburg-Vorpommern | 12.80 | 54.23 | 1 |
| 4122623 | *Streptopelia turtur* | Mecklenburg-Vorpommern | 12.78 | 54.25 | 1 |
| 4122634 | *Streptopelia turtur* | Mecklenburg-Vorpommern | 12.79 | 54.25 | 1 |
| 4122671 | *Streptopelia turtur* | Mecklenburg-Vorpommern | 12.83 | 54.24 | 1 |
| 4122726 | *Streptopelia turtur* | Mecklenburg-Vorpommern | 12.83 | 54.26 | 1 |
| 5337183 | *Streptopelia turtur* | Mecklenburg-Vorpommern | 11.98 | 53.36 | 1 |
| 9501343 | *Streptopelia turtur* | Mecklenburg-Vorpommern | 12.81 | 54.25 | 1 |
| 9675895 | *Streptopelia turtur* | Mecklenburg-Vorpommern | 11.18 | 53.31 | 1 |
| 9999807 | *Streptopelia turtur* | Mecklenburg-Vorpommern | 13.77 | 53.55 | 1 |
| 10139069 | *Streptopelia turtur* | Mecklenburg-Vorpommern | 13.46 | 53.53 | 1 |
| 10583968 | *Streptopelia turtur* | Mecklenburg-Vorpommern | 11.47 | 53.25 | 1 |
| 3523 | *Streptopelia turtur* | Niedersachsen | 8.98 | 52.43 | 1 |
| 38489 | *Streptopelia turtur* | Niedersachsen | 7.43 | 52.58 | 1 |
| 38869 | *Streptopelia turtur* | Niedersachsen | 7.42 | 52.59 | 1 |
| 42612 | *Streptopelia turtur* | Niedersachsen | 7.42 | 52.59 | 1 |
| 167490 | *Streptopelia turtur* | Niedersachsen | 7.38 | 52.61 | 1 |
| 315193 | *Streptopelia turtur* | Niedersachsen | 9.90 | 52.02 | 1 |

| ID | species | Country | long | lat | PO |
|---|---|---|---|---|---|
| 430851 | *Streptopelia turtur* | Niedersachsen | 9.12 | 52.52 | 1 |
| 500617 | *Streptopelia turtur* | Niedersachsen | 7.47 | 53.09 | 1 |
| 591596 | *Streptopelia turtur* | Niedersachsen | 7.58 | 52.63 | 1 |
| 966899 | *Streptopelia turtur* | Niedersachsen | 7.40 | 52.87 | 1 |
| 1752917 | *Streptopelia turtur* | Niedersachsen | 9.67 | 51.77 | 1 |
| 1757904 | *Streptopelia turtur* | Niedersachsen | 11.12 | 53.18 | 1 |
| 1760821 | *Streptopelia turtur* | Niedersachsen | 7.48 | 52.90 | 1 |
| 1765872 | *Streptopelia turtur* | Niedersachsen | 10.07 | 51.55 | 1 |
| 1777810 | *Streptopelia turtur* | Niedersachsen | 7.44 | 52.90 | 1 |
| 1796439 | *Streptopelia turtur* | Niedersachsen | 9.89 | 52.55 | 1 |
| 1799092 | *Streptopelia turtur* | Niedersachsen | 9.81 | 52.60 | 1 |
| 1799455 | *Streptopelia turtur* | Niedersachsen | 9.88 | 52.61 | 1 |
| 1806025 | *Streptopelia turtur* | Niedersachsen | 11.03 | 53.27 | 1 |
| 1825135 | *Streptopelia turtur* | Niedersachsen | 10.78 | 53.09 | 1 |
| 1857923 | *Streptopelia turtur* | Niedersachsen | 7.21 | 52.41 | 1 |
| 1860664 | *Streptopelia turtur* | Niedersachsen | 9.19 | 52.28 | 1 |
| 1875978 | *Streptopelia turtur* | Niedersachsen | 10.54 | 52.85 | 1 |
| 1917120 | *Streptopelia turtur* | Niedersachsen | 9.64 | 51.58 | 1 |

| **ID** | **species** | **Country** | **long** | **lat** | **PO** |
|---|---|---|---|---|---|
| 1917943 | *Streptopelia turtur* | Niedersachsen | 10.58 | 52.85 | 1 |
| 1927689 | *Streptopelia turtur* | Niedersachsen | 8.16 | 52.43 | 1 |
| 1942561 | *Streptopelia turtur* | Niedersachsen | 7.20 | 52.42 | 1 |
| 1944327 | *Streptopelia turtur* | Niedersachsen | 7.43 | 52.88 | 1 |
| 1946896 | *Streptopelia turtur* | Niedersachsen | 10.30 | 52.03 | 1 |
| 1957022 | *Streptopelia turtur* | Niedersachsen | 10.80 | 52.30 | 1 |
| 1994866 | *Streptopelia turtur* | Niedersachsen | 8.04 | 52.71 | 1 |
| 2024528 | *Streptopelia turtur* | Niedersachsen | 9.77 | 52.11 | 1 |
| 2042971 | *Streptopelia turtur* | Niedersachsen | 7.22 | 52.43 | 1 |
| 2046819 | *Streptopelia turtur* | Niedersachsen | 8.16 | 52.43 | 1 |
| 2678531 | *Streptopelia turtur* | Niedersachsen | 7.64 | 53.01 | 1 |
| 3799798 | *Streptopelia turtur* | Niedersachsen | 7.06 | 52.54 | 1 |
| 5142781 | *Streptopelia turtur* | Niedersachsen | 10.52 | 52.59 | 1 |
| 5279592 | *Streptopelia turtur* | Niedersachsen | 9.25 | 52.60 | 1 |
| 5280523 | *Streptopelia turtur* | Niedersachsen | 8.04 | 52.92 | 1 |
| 5301162 | *Streptopelia turtur* | Niedersachsen | 10.43 | 52.94 | 1 |
| 5313850 | *Streptopelia turtur* | Niedersachsen | 10.43 | 52.94 | 1 |
| 5364579 | *Streptopelia turtur* | Niedersachsen | 10.55 | 52.03 | 1 |

| **ID** | **species** | **Country** | **long** | **lat** | **PO** |
|---|---|---|---|---|---|
| 5375045 | *Streptopelia turtur* | Niedersachsen | 8.85 | 52.52 | 1 |
| 5390390 | *Streptopelia turtur* | Niedersachsen | 9.76 | 52.10 | 1 |
| 5433076 | *Streptopelia turtur* | Niedersachsen | 9.34 | 52.88 | 1 |
| 5444647 | *Streptopelia turtur* | Niedersachsen | 10.51 | 52.63 | 1 |
| 5445488 | *Streptopelia turtur* | Niedersachsen | 10.73 | 52.83 | 1 |
| 5454374 | *Streptopelia turtur* | Niedersachsen | 10.08 | 51.55 | 1 |
| 5462759 | *Streptopelia turtur* | Niedersachsen | 9.71 | 51.81 | 1 |
| 5469975 | *Streptopelia turtur* | Niedersachsen | 10.45 | 52.51 | 1 |
| 5479864 | *Streptopelia turtur* | Niedersachsen | 10.43 | 52.93 | 1 |
| 5507850 | *Streptopelia turtur* | Niedersachsen | 9.82 | 52.73 | 1 |
| 5508948 | *Streptopelia turtur* | Niedersachsen | 9.82 | 52.71 | 1 |
| 5509406 | *Streptopelia turtur* | Niedersachsen | 9.79 | 52.72 | 1 |
| 5524911 | *Streptopelia turtur* | Niedersachsen | 10.68 | 53.17 | 1 |
| 5528341 | *Streptopelia turtur* | Niedersachsen | 7.23 | 52.43 | 1 |
| 5572288 | *Streptopelia turtur* | Niedersachsen | 10.30 | 53.11 | 1 |
| 5572290 | *Streptopelia turtur* | Niedersachsen | 10.30 | 53.12 | 1 |
| 5578950 | *Streptopelia turtur* | Niedersachsen | 9.99 | 51.40 | 1 |
| 5599488 | *Streptopelia turtur* | Niedersachsen | 10.68 | 53.20 | 1 |

| **ID** | **species** | **Country** | **long** | **lat** | **PO** |
|---|---|---|---|---|---|
| 5602690 | *Streptopelia turtur* | Niedersachsen | 8.12 | 52.66 | 1 |
| 5609612 | *Streptopelia turtur* | Niedersachsen | 9.33 | 52.68 | 1 |
| 5634065 | *Streptopelia turtur* | Niedersachsen | 9.86 | 52.62 | 1 |
| 5683710 | *Streptopelia turtur* | Niedersachsen | 7.67 | 53.47 | 1 |
| 5700150 | *Streptopelia turtur* | Niedersachsen | 10.81 | 52.91 | 1 |
| 5710598 | *Streptopelia turtur* | Niedersachsen | 10.73 | 52.92 | 1 |
| 5720322 | *Streptopelia turtur* | Niedersachsen | 10.69 | 53.21 | 1 |
| 5803762 | *Streptopelia turtur* | Niedersachsen | 8.58 | 53.85 | 1 |
| 5851599 | *Streptopelia turtur* | Niedersachsen | 7.11 | 52.62 | 1 |
| 5886468 | *Streptopelia turtur* | Niedersachsen | 10.00 | 51.93 | 1 |
| 6524621 | *Streptopelia turtur* | Niedersachsen | 8.86 | 52.50 | 1 |
| 6610185 | *Streptopelia turtur* | Niedersachsen | 9.79 | 52.14 | 1 |
| 7684733 | *Streptopelia turtur* | Niedersachsen | 9.78 | 52.71 | 1 |
| 8511572 | *Streptopelia turtur* | Niedersachsen | 9.78 | 52.92 | 1 |
| 8790429 | *Streptopelia turtur* | Niedersachsen | 10.00 | 52.90 | 1 |
| 8791007 | *Streptopelia turtur* | Niedersachsen | 9.76 | 53.00 | 1 |
| 9487261 | *Streptopelia turtur* | Niedersachsen | 10.72 | 52.98 | 1 |
| 9520640 | *Streptopelia turtur* | Niedersachsen | 9.16 | 52.53 | 1 |

| ID | species | Country | long | lat | PO |
|---|---|---|---|---|---|
| 9584224 | *Streptopelia turtur* | Niedersachsen | 11.25 | 52.96 | 1 |
| 9584242 | *Streptopelia turtur* | Niedersachsen | 11.19 | 52.92 | 1 |
| 9584265 | *Streptopelia turtur* | Niedersachsen | 10.93 | 53.04 | 1 |
| 9584292 | *Streptopelia turtur* | Niedersachsen | 10.94 | 53.03 | 1 |
| 9591106 | *Streptopelia turtur* | Niedersachsen | 10.80 | 53.07 | 1 |
| 9629639 | *Streptopelia turtur* | Niedersachsen | 11.05 | 53.21 | 1 |
| 9646588 | *Streptopelia turtur* | Niedersachsen | 7.86 | 52.85 | 1 |
| 9668644 | *Streptopelia turtur* | Niedersachsen | 8.41 | 52.74 | 1 |
| 9669320 | *Streptopelia turtur* | Niedersachsen | 10.25 | 53.10 | 1 |
| 9682349 | *Streptopelia turtur* | Niedersachsen | 11.04 | 53.05 | 1 |
| 9685748 | *Streptopelia turtur* | Niedersachsen | 10.99 | 53.03 | 1 |
| 9701350 | *Streptopelia turtur* | Niedersachsen | 10.97 | 53.03 | 1 |
| 9701497 | *Streptopelia turtur* | Niedersachsen | 10.95 | 53.02 | 1 |
| 9712718 | *Streptopelia turtur* | Niedersachsen | 9.20 | 52.75 | 1 |
| 9729346 | *Streptopelia turtur* | Niedersachsen | 9.79 | 52.62 | 1 |
| 9757416 | *Streptopelia turtur* | Niedersachsen | 10.70 | 52.90 | 1 |
| 9774566 | *Streptopelia turtur* | Niedersachsen | 10.71 | 52.88 | 1 |
| 9787709 | *Streptopelia turtur* | Niedersachsen | 7.12 | 52.59 | 1 |

| ID | species | Country | long | lat | PO |
|---|---|---|---|---|---|
| 9790045 | *Streptopelia turtur* | Niedersachsen | 10.77 | 53.08 | 1 |
| 9802306 | *Streptopelia turtur* | Niedersachsen | 7.47 | 52.37 | 1 |
| 9805501 | *Streptopelia turtur* | Niedersachsen | 9.67 | 53.09 | 1 |
| 9809793 | *Streptopelia turtur* | Niedersachsen | 7.03 | 52.61 | 1 |
| 9849844 | *Streptopelia turtur* | Niedersachsen | 10.80 | 53.10 | 1 |
| 9877790 | *Streptopelia turtur* | Niedersachsen | 11.03 | 53.10 | 1 |
| 9878224 | *Streptopelia turtur* | Niedersachsen | 10.98 | 53.07 | 1 |
| 9903058 | *Streptopelia turtur* | Niedersachsen | 10.10 | 52.46 | 1 |
| 9917932 | *Streptopelia turtur* | Niedersachsen | 9.36 | 52.71 | 1 |
| 9930884 | *Streptopelia turtur* | Niedersachsen | 8.33 | 52.92 | 1 |
| 9933755 | *Streptopelia turtur* | Niedersachsen | 7.09 | 52.54 | 1 |
| 9933807 | *Streptopelia turtur* | Niedersachsen | 7.18 | 52.43 | 1 |
| 9938966 | *Streptopelia turtur* | Niedersachsen | 10.14 | 53.10 | 1 |
| 9943096 | *Streptopelia turtur* | Niedersachsen | 10.52 | 51.95 | 1 |
| 9954428 | *Streptopelia turtur* | Niedersachsen | 9.50 | 52.13 | 1 |
| 9963996 | *Streptopelia turtur* | Niedersachsen | 8.31 | 52.55 | 1 |
| 9973725 | *Streptopelia turtur* | Niedersachsen | 9.46 | 52.17 | 1 |
| 9987281 | *Streptopelia turtur* | Niedersachsen | 7.09 | 52.54 | 1 |

| **ID** | **species** | **Country** | **long** | **lat** | **PO** |
|---|---|---|---|---|---|
| 9995553 | *Streptopelia turtur* | Niedersachsen | 7.19 | 52.41 | 1 |
| 3331 | *Streptopelia turtur* | Nordrhein-Westfalen | 7.60 | 51.84 | 1 |
| 634949 | *Streptopelia turtur* | Nordrhein-Westfalen | 8.18 | 51.76 | 1 |
| 642749 | *Streptopelia turtur* | Nordrhein-Westfalen | 8.27 | 51.78 | 1 |
| 783334 | *Streptopelia turtur* | Nordrhein-Westfalen | 8.17 | 51.74 | 1 |
| 783363 | *Streptopelia turtur* | Nordrhein-Westfalen | 8.17 | 51.74 | 1 |
| 1214426 | *Streptopelia turtur* | Nordrhein-Westfalen | 8.31 | 51.71 | 1 |
| 1706550 | *Streptopelia turtur* | Nordrhein-Westfalen | 7.43 | 50.83 | 1 |
| 1707364 | *Streptopelia turtur* | Nordrhein-Westfalen | 6.74 | 51.82 | 1 |
| 1708516 | *Streptopelia turtur* | Nordrhein-Westfalen | 6.97 | 51.78 | 1 |
| 1750480 | *Streptopelia turtur* | Nordrhein-Westfalen | 8.22 | 51.73 | 1 |
| 1758186 | *Streptopelia turtur* | Nordrhein-Westfalen | 8.68 | 51.18 | 1 |
| 1773602 | *Streptopelia turtur* | Nordrhein-Westfalen | 6.49 | 50.39 | 1 |
| 1800284 | *Streptopelia turtur* | Nordrhein-Westfalen | 9.05 | 52.00 | 1 |
| 1821734 | *Streptopelia turtur* | Nordrhein-Westfalen | 7.69 | 51.87 | 1 |
| 1833470 | *Streptopelia turtur* | Nordrhein-Westfalen | 7.43 | 51.75 | 1 |
| 1851489 | *Streptopelia turtur* | Nordrhein-Westfalen | 8.64 | 51.20 | 1 |
| 1859459 | *Streptopelia turtur* | Nordrhein-Westfalen | 8.68 | 51.20 | 1 |

| **ID** | **species** | **Country** | **long** | **lat** | **PO** |
|---|---|---|---|---|---|
| 1859720 | *Streptopelia turtur* | Nordrhein-Westfalen | 6.30 | 51.53 | 1 |
| 1888221 | *Streptopelia turtur* | Nordrhein-Westfalen | 7.31 | 52.25 | 1 |
| 1901821 | *Streptopelia turtur* | Nordrhein-Westfalen | 6.69 | 51.32 | 1 |
| 1915682 | *Streptopelia turtur* | Nordrhein-Westfalen | 6.76 | 50.75 | 1 |
| 1917016 | *Streptopelia turtur* | Nordrhein-Westfalen | 8.70 | 51.20 | 1 |
| 1926989 | *Streptopelia turtur* | Nordrhein-Westfalen | 8.24 | 51.74 | 1 |
| 1973245 | *Streptopelia turtur* | Nordrhein-Westfalen | 7.13 | 50.89 | 1 |
| 1976507 | *Streptopelia turtur* | Nordrhein-Westfalen | 9.19 | 51.90 | 1 |
| 2015417 | *Streptopelia turtur* | Nordrhein-Westfalen | 7.98 | 51.69 | 1 |
| 2037205 | *Streptopelia turtur* | Nordrhein-Westfalen | 9.00 | 52.11 | 1 |
| 2114879 | *Streptopelia turtur* | Nordrhein-Westfalen | 8.23 | 51.72 | 1 |
| 2416452 | *Streptopelia turtur* | Nordrhein-Westfalen | 8.23 | 51.53 | 1 |
| 3043319 | *Streptopelia turtur* | Nordrhein-Westfalen | 8.75 | 51.20 | 1 |
| 3608599 | *Streptopelia turtur* | Nordrhein-Westfalen | 8.19 | 51.74 | 1 |
| 4758801 | *Streptopelia turtur* | Nordrhein-Westfalen | 6.92 | 51.25 | 1 |
| 5306716 | *Streptopelia turtur* | Nordrhein-Westfalen | 6.82 | 50.60 | 1 |
| 5351834 | *Streptopelia turtur* | Nordrhein-Westfalen | 6.65 | 51.31 | 1 |
| 5366921 | *Streptopelia turtur* | Nordrhein-Westfalen | 6.19 | 51.34 | 1 |

| **ID** | **species** | **Country** | **long** | **lat** | **PO** |
|---|---|---|---|---|---|
| 5366925 | *Streptopelia turtur* | Nordrhein-Westfalen | 7.75 | 51.77 | 1 |
| 5370006 | *Streptopelia turtur* | Nordrhein-Westfalen | 6.37 | 51.37 | 1 |
| 5404568 | *Streptopelia turtur* | Nordrhein-Westfalen | 6.28 | 50.64 | 1 |
| 5417472 | *Streptopelia turtur* | Nordrhein-Westfalen | 8.21 | 51.27 | 1 |
| 5438361 | *Streptopelia turtur* | Nordrhein-Westfalen | 6.67 | 51.82 | 1 |
| 5462148 | *Streptopelia turtur* | Nordrhein-Westfalen | 9.21 | 51.94 | 1 |
| 5462287 | *Streptopelia turtur* | Nordrhein-Westfalen | 9.12 | 51.94 | 1 |
| 5481633 | *Streptopelia turtur* | Nordrhein-Westfalen | 6.92 | 52.05 | 1 |
| 5509263 | *Streptopelia turtur* | Nordrhein-Westfalen | 6.27 | 50.63 | 1 |
| 5522036 | *Streptopelia turtur* | Nordrhein-Westfalen | 7.12 | 50.89 | 1 |
| 5538780 | *Streptopelia turtur* | Nordrhein-Westfalen | 6.69 | 51.79 | 1 |
| 5540133 | *Streptopelia turtur* | Nordrhein-Westfalen | 6.28 | 51.33 | 1 |
| 5555640 | *Streptopelia turtur* | Nordrhein-Westfalen | 8.30 | 51.30 | 1 |
| 5565351 | *Streptopelia turtur* | Nordrhein-Westfalen | 7.09 | 50.79 | 1 |
| 5581687 | *Streptopelia turtur* | Nordrhein-Westfalen | 9.17 | 51.87 | 1 |
| 5582155 | *Streptopelia turtur* | Nordrhein-Westfalen | 6.37 | 50.64 | 1 |
| 5616972 | *Streptopelia turtur* | Nordrhein-Westfalen | 5.99 | 51.82 | 1 |
| 5638944 | *Streptopelia turtur* | Nordrhein-Westfalen | 7.16 | 50.87 | 1 |

| **ID** | **species** | **Country** | **long** | **lat** | **PO** |
|---|---|---|---|---|---|
| 5668540 | *Streptopelia turtur* | Nordrhein-Westfalen | 7.03 | 50.80 | 1 |
| 5675317 | *Streptopelia turtur* | Nordrhein-Westfalen | 6.29 | 50.66 | 1 |
| 5676277 | *Streptopelia turtur* | Nordrhein-Westfalen | 7.13 | 51.82 | 1 |
| 5678416 | *Streptopelia turtur* | Nordrhein-Westfalen | 6.26 | 51.35 | 1 |
| 5684048 | *Streptopelia turtur* | Nordrhein-Westfalen | 6.23 | 50.57 | 1 |
| 5712600 | *Streptopelia turtur* | Nordrhein-Westfalen | 6.52 | 50.74 | 1 |
| 5770475 | *Streptopelia turtur* | Nordrhein-Westfalen | 7.16 | 50.89 | 1 |
| 5950731 | *Streptopelia turtur* | Nordrhein-Westfalen | 7.60 | 51.39 | 1 |
| 6257866 | *Streptopelia turtur* | Nordrhein-Westfalen | 6.62 | 51.39 | 1 |
| 6502667 | *Streptopelia turtur* | Nordrhein-Westfalen | 7.80 | 51.47 | 1 |
| 6880578 | *Streptopelia turtur* | Nordrhein-Westfalen | 8.68 | 51.19 | 1 |
| 7217701 | *Streptopelia turtur* | Nordrhein-Westfalen | 9.24 | 51.80 | 1 |
| 7277191 | *Streptopelia turtur* | Nordrhein-Westfalen | 7.95 | 51.41 | 1 |
| 9326017 | *Streptopelia turtur* | Nordrhein-Westfalen | 7.40 | 51.80 | 1 |
| 9326020 | *Streptopelia turtur* | Nordrhein-Westfalen | 7.40 | 51.80 | 1 |
| 9326044 | *Streptopelia turtur* | Nordrhein-Westfalen | 7.45 | 51.76 | 1 |
| 9493850 | *Streptopelia turtur* | Nordrhein-Westfalen | 9.02 | 52.11 | 1 |
| 9494959 | *Streptopelia turtur* | Nordrhein-Westfalen | 7.75 | 51.77 | 1 |

| **ID** | **species** | **Country** | **long** | **lat** | **PO** |
|---|---|---|---|---|---|
| 9496560 | *Streptopelia turtur* | Nordrhein-Westfalen | 8.20 | 51.20 | 1 |
| 9498634 | *Streptopelia turtur* | Nordrhein-Westfalen | 8.55 | 52.45 | 1 |
| 9524951 | *Streptopelia turtur* | Nordrhein-Westfalen | 6.32 | 50.67 | 1 |
| 9535823 | *Streptopelia turtur* | Nordrhein-Westfalen | 9.23 | 51.95 | 1 |
| 9550337 | *Streptopelia turtur* | Nordrhein-Westfalen | 6.24 | 51.13 | 1 |
| 9567636 | *Streptopelia turtur* | Nordrhein-Westfalen | 6.57 | 51.84 | 1 |
| 9573340 | *Streptopelia turtur* | Nordrhein-Westfalen | 7.16 | 51.82 | 1 |
| 9577381 | *Streptopelia turtur* | Nordrhein-Westfalen | 7.17 | 50.88 | 1 |
| 9585564 | *Streptopelia turtur* | Nordrhein-Westfalen | 7.10 | 52.09 | 1 |
| 9588055 | *Streptopelia turtur* | Nordrhein-Westfalen | 6.87 | 51.62 | 1 |
| 9595529 | *Streptopelia turtur* | Nordrhein-Westfalen | 6.03 | 51.70 | 1 |
| 9612149 | *Streptopelia turtur* | Nordrhein-Westfalen | 8.35 | 51.17 | 1 |
| 9620185 | *Streptopelia turtur* | Nordrhein-Westfalen | 6.55 | 50.38 | 1 |
| 9626515 | *Streptopelia turtur* | Nordrhein-Westfalen | 6.19 | 51.01 | 1 |
| 9628203 | *Streptopelia turtur* | Nordrhein-Westfalen | 6.21 | 51.32 | 1 |
| 9646459 | *Streptopelia turtur* | Nordrhein-Westfalen | 8.23 | 51.28 | 1 |
| 9655707 | *Streptopelia turtur* | Nordrhein-Westfalen | 6.15 | 51.30 | 1 |
| 9656658 | *Streptopelia turtur* | Nordrhein-Westfalen | 7.53 | 51.86 | 1 |

| **ID** | **species** | **Country** | **long** | **lat** | **PO** |
|---|---|---|---|---|---|
| 9666292 | *Streptopelia turtur* | Nordrhein-Westfalen | 6.85 | 51.72 | 1 |
| 9695038 | *Streptopelia turtur* | Nordrhein-Westfalen | 6.39 | 50.54 | 1 |
| 9695518 | *Streptopelia turtur* | Nordrhein-Westfalen | 7.09 | 50.84 | 1 |
| 9702469 | *Streptopelia turtur* | Nordrhein-Westfalen | 6.37 | 50.76 | 1 |
| 9704023 | *Streptopelia turtur* | Nordrhein-Westfalen | 6.73 | 51.80 | 1 |
| 9705678 | *Streptopelia turtur* | Nordrhein-Westfalen | 7.87 | 51.86 | 1 |
| 9711491 | *Streptopelia turtur* | Nordrhein-Westfalen | 6.21 | 51.11 | 1 |
| 9712996 | *Streptopelia turtur* | Nordrhein-Westfalen | 6.59 | 50.99 | 1 |
| 9713078 | *Streptopelia turtur* | Nordrhein-Westfalen | 6.10 | 51.71 | 1 |
| 9718964 | *Streptopelia turtur* | Nordrhein-Westfalen | 6.53 | 50.48 | 1 |
| 9719536 | *Streptopelia turtur* | Nordrhein-Westfalen | 6.61 | 50.72 | 1 |
| 9719735 | *Streptopelia turtur* | Nordrhein-Westfalen | 6.60 | 50.69 | 1 |
| 9723481 | *Streptopelia turtur* | Nordrhein-Westfalen | 8.22 | 51.30 | 1 |
| 9732104 | *Streptopelia turtur* | Nordrhein-Westfalen | 7.32 | 52.28 | 1 |
| 9738194 | *Streptopelia turtur* | Nordrhein-Westfalen | 6.54 | 50.71 | 1 |
| 9741921 | *Streptopelia turtur* | Nordrhein-Westfalen | 6.76 | 50.73 | 1 |
| 9745948 | *Streptopelia turtur* | Nordrhein-Westfalen | 8.90 | 51.61 | 1 |
| 9745953 | *Streptopelia turtur* | Nordrhein-Westfalen | 8.92 | 51.60 | 1 |

| **ID** | **species** | **Country** | **long** | **lat** | **PO** |
|---|---|---|---|---|---|
| 9748563 | *Streptopelia turtur* | Nordrhein-Westfalen | 9.21 | 51.71 | 1 |
| 9765998 | *Streptopelia turtur* | Nordrhein-Westfalen | 6.21 | 51.01 | 1 |
| 9790412 | *Streptopelia turtur* | Nordrhein-Westfalen | 6.60 | 50.40 | 1 |
| 9795327 | *Streptopelia turtur* | Nordrhein-Westfalen | 8.27 | 51.19 | 1 |
| 9806952 | *Streptopelia turtur* | Nordrhein-Westfalen | 8.26 | 51.30 | 1 |
| 9816044 | *Streptopelia turtur* | Nordrhein-Westfalen | 6.05 | 51.70 | 1 |
| 9823751 | *Streptopelia turtur* | Nordrhein-Westfalen | 6.77 | 51.84 | 1 |
| 9849858 | *Streptopelia turtur* | Nordrhein-Westfalen | 8.51 | 51.40 | 1 |
| 9849890 | *Streptopelia turtur* | Nordrhein-Westfalen | 8.53 | 51.40 | 1 |
| 9858931 | *Streptopelia turtur* | Nordrhein-Westfalen | 8.84 | 51.42 | 1 |
| 9859927 | *Streptopelia turtur* | Nordrhein-Westfalen | 6.66 | 50.66 | 1 |
| 9877760 | *Streptopelia turtur* | Nordrhein-Westfalen | 6.76 | 50.57 | 1 |
| 9902036 | *Streptopelia turtur* | Nordrhein-Westfalen | 6.20 | 51.56 | 1 |
| 9921109 | *Streptopelia turtur* | Nordrhein-Westfalen | 9.00 | 52.44 | 1 |
| 9924841 | *Streptopelia turtur* | Nordrhein-Westfalen | 8.07 | 51.33 | 1 |
| 9940645 | *Streptopelia turtur* | Nordrhein-Westfalen | 8.71 | 51.17 | 1 |
| 9945109 | *Streptopelia turtur* | Nordrhein-Westfalen | 6.23 | 51.01 | 1 |
| 9946770 | *Streptopelia turtur* | Nordrhein-Westfalen | 6.50 | 50.38 | 1 |

| **ID** | **species** | **Country** | **long** | **lat** | **PO** |
|---|---|---|---|---|---|
| 9954003 | *Streptopelia turtur* | Nordrhein-Westfalen | 8.25 | 51.18 | 1 |
| 9974571 | *Streptopelia turtur* | Nordrhein-Westfalen | 7.02 | 51.69 | 1 |
| 9976881 | *Streptopelia turtur* | Nordrhein-Westfalen | 6.27 | 50.62 | 1 |
| 9980577 | *Streptopelia turtur* | Nordrhein-Westfalen | 8.12 | 51.35 | 1 |
| 9984543 | *Streptopelia turtur* | Nordrhein-Westfalen | 8.66 | 51.19 | 1 |
| 9997597 | *Streptopelia turtur* | Nordrhein-Westfalen | 7.17 | 50.87 | 1 |
| 10015983 | *Streptopelia turtur* | Nordrhein-Westfalen | 6.14 | 51.28 | 1 |
| 10016062 | *Streptopelia turtur* | Nordrhein-Westfalen | 6.13 | 51.27 | 1 |
| 10041828 | *Streptopelia turtur* | Nordrhein-Westfalen | 6.66 | 50.42 | 1 |
| 10064074 | *Streptopelia turtur* | Nordrhein-Westfalen | 6.71 | 50.66 | 1 |
| 10817690 | *Streptopelia turtur* | Nordrhein-Westfalen | 8.70 | 51.13 | 1 |
| 11123792 | *Streptopelia turtur* | Nordrhein-Westfalen | 8.73 | 51.22 | 1 |
| 129647 | *Streptopelia turtur* | Rheinland-Pfalz | 7.44 | 49.23 | 1 |
| 137928 | *Streptopelia turtur* | Rheinland-Pfalz | 7.22 | 49.57 | 1 |
| 1712173 | *Streptopelia turtur* | Rheinland-Pfalz | 6.77 | 50.45 | 1 |
| 1715073 | *Streptopelia turtur* | Rheinland-Pfalz | 6.91 | 49.83 | 1 |
| 1734067 | *Streptopelia turtur* | Rheinland-Pfalz | 7.68 | 49.57 | 1 |
| 1813008 | *Streptopelia turtur* | Rheinland-Pfalz | 7.72 | 49.09 | 1 |

| **ID** | **species** | **Country** | **long** | **lat** | **PO** |
|---|---|---|---|---|---|
| 1846545 | *Streptopelia turtur* | Rheinland-Pfalz | 7.80 | 50.59 | 1 |
| 1859389 | *Streptopelia turtur* | Rheinland-Pfalz | 6.53 | 49.64 | 1 |
| 1865759 | *Streptopelia turtur* | Rheinland-Pfalz | 7.80 | 50.57 | 1 |
| 1870046 | *Streptopelia turtur* | Rheinland-Pfalz | 7.01 | 50.16 | 1 |
| 1872943 | *Streptopelia turtur* | Rheinland-Pfalz | 7.87 | 50.62 | 1 |
| 1883142 | *Streptopelia turtur* | Rheinland-Pfalz | 7.99 | 50.38 | 1 |
| 1897659 | *Streptopelia turtur* | Rheinland-Pfalz | 6.19 | 49.99 | 1 |
| 1898211 | *Streptopelia turtur* | Rheinland-Pfalz | 6.70 | 49.75 | 1 |
| 1903772 | *Streptopelia turtur* | Rheinland-Pfalz | 6.57 | 49.77 | 1 |
| 1910462 | *Streptopelia turtur* | Rheinland-Pfalz | 8.36 | 49.83 | 1 |
| 1914988 | *Streptopelia turtur* | Rheinland-Pfalz | 6.59 | 50.29 | 1 |
| 1920170 | *Streptopelia turtur* | Rheinland-Pfalz | 8.37 | 49.84 | 1 |
| 1920762 | *Streptopelia turtur* | Rheinland-Pfalz | 7.69 | 50.63 | 1 |
| 1920813 | *Streptopelia turtur* | Rheinland-Pfalz | 6.70 | 49.81 | 1 |
| 1933011 | *Streptopelia turtur* | Rheinland-Pfalz | 8.33 | 49.89 | 1 |
| 1948331 | *Streptopelia turtur* | Rheinland-Pfalz | 6.54 | 49.76 | 1 |
| 1951459 | *Streptopelia turtur* | Rheinland-Pfalz | 8.31 | 49.84 | 1 |
| 1963331 | *Streptopelia turtur* | Rheinland-Pfalz | 7.79 | 50.66 | 1 |

| **ID** | **species** | **Country** | **long** | **lat** | **PO** |
|---|---|---|---|---|---|
| 1980121 | *Streptopelia turtur* | Rheinland-Pfalz | 7.15 | 50.43 | 1 |
| 1981791 | *Streptopelia turtur* | Rheinland-Pfalz | 6.98 | 50.22 | 1 |
| 1994533 | *Streptopelia turtur* | Rheinland-Pfalz | 8.28 | 49.53 | 1 |
| 2000379 | *Streptopelia turtur* | Rheinland-Pfalz | 8.17 | 49.97 | 1 |
| 2013631 | *Streptopelia turtur* | Rheinland-Pfalz | 6.90 | 50.03 | 1 |
| 2015285 | *Streptopelia turtur* | Rheinland-Pfalz | 8.16 | 49.90 | 1 |
| 2027144 | *Streptopelia turtur* | Rheinland-Pfalz | 6.72 | 49.81 | 1 |
| 2035156 | *Streptopelia turtur* | Rheinland-Pfalz | 7.52 | 50.52 | 1 |
| 2037463 | *Streptopelia turtur* | Rheinland-Pfalz | 6.46 | 49.61 | 1 |
| 2040832 | *Streptopelia turtur* | Rheinland-Pfalz | 8.29 | 49.56 | 1 |
| 2041161 | *Streptopelia turtur* | Rheinland-Pfalz | 6.26 | 50.21 | 1 |
| 2044005 | *Streptopelia turtur* | Rheinland-Pfalz | 7.75 | 49.57 | 1 |
| 2063029 | *Streptopelia turtur* | Rheinland-Pfalz | 7.77 | 50.60 | 1 |
| 2084825 | *Streptopelia turtur* | Rheinland-Pfalz | 8.38 | 49.81 | 1 |
| 2086964 | *Streptopelia turtur* | Rheinland-Pfalz | 7.70 | 50.56 | 1 |
| 2087142 | *Streptopelia turtur* | Rheinland-Pfalz | 8.28 | 49.53 | 1 |
| 2087657 | *Streptopelia turtur* | Rheinland-Pfalz | 7.51 | 50.41 | 1 |
| 2164007 | *Streptopelia turtur* | Rheinland-Pfalz | 7.31 | 50.06 | 1 |

| **ID** | **species** | **Country** | **long** | **lat** | **PO** |
|---|---|---|---|---|---|
| 2465367 | *Streptopelia turtur* | Rheinland-Pfalz | 8.41 | 49.27 | 1 |
| 2538844 | *Streptopelia turtur* | Rheinland-Pfalz | 8.03 | 50.35 | 1 |
| 3212378 | *Streptopelia turtur* | Rheinland-Pfalz | 8.21 | 49.47 | 1 |
| 5296871 | *Streptopelia turtur* | Rheinland-Pfalz | 8.04 | 49.97 | 1 |
| 5305680 | *Streptopelia turtur* | Rheinland-Pfalz | 7.52 | 49.15 | 1 |
| 5332179 | *Streptopelia turtur* | Rheinland-Pfalz | 6.79 | 50.45 | 1 |
| 5337621 | *Streptopelia turtur* | Rheinland-Pfalz | 7.84 | 50.64 | 1 |
| 5369301 | *Streptopelia turtur* | Rheinland-Pfalz | 7.19 | 49.65 | 1 |
| 5388722 | *Streptopelia turtur* | Rheinland-Pfalz | 7.29 | 49.42 | 1 |
| 5405770 | *Streptopelia turtur* | Rheinland-Pfalz | 8.28 | 49.53 | 1 |
| 5405782 | *Streptopelia turtur* | Rheinland-Pfalz | 8.23 | 49.52 | 1 |
| 5405816 | *Streptopelia turtur* | Rheinland-Pfalz | 8.24 | 49.48 | 1 |
| 5457207 | *Streptopelia turtur* | Rheinland-Pfalz | 7.21 | 49.63 | 1 |
| 5472464 | *Streptopelia turtur* | Rheinland-Pfalz | 8.31 | 49.94 | 1 |
| 5488299 | *Streptopelia turtur* | Rheinland-Pfalz | 7.70 | 49.56 | 1 |
| 5524917 | *Streptopelia turtur* | Rheinland-Pfalz | 6.76 | 49.74 | 1 |
| 5528688 | *Streptopelia turtur* | Rheinland-Pfalz | 7.21 | 49.66 | 1 |
| 5536190 | *Streptopelia turtur* | Rheinland-Pfalz | 7.31 | 49.42 | 1 |

| **ID** | **species** | **Country** | **long** | **lat** | **PO** |
|---|---|---|---|---|---|
| 5539415 | *Streptopelia turtur* | Rheinland-Pfalz | 7.82 | 50.64 | 1 |
| 5582898 | *Streptopelia turtur* | Rheinland-Pfalz | 8.15 | 49.96 | 1 |
| 5599921 | *Streptopelia turtur* | Rheinland-Pfalz | 7.55 | 50.69 | 1 |
| 5606971 | *Streptopelia turtur* | Rheinland-Pfalz | 6.48 | 49.89 | 1 |
| 5656997 | *Streptopelia turtur* | Rheinland-Pfalz | 7.80 | 50.63 | 1 |
| 5683280 | *Streptopelia turtur* | Rheinland-Pfalz | 6.57 | 49.77 | 1 |
| 5716046 | *Streptopelia turtur* | Rheinland-Pfalz | 8.28 | 49.62 | 1 |
| 5862641 | *Streptopelia turtur* | Rheinland-Pfalz | 7.77 | 50.03 | 1 |
| 7365216 | *Streptopelia turtur* | Rheinland-Pfalz | 8.10 | 50.59 | 1 |
| 9490133 | *Streptopelia turtur* | Rheinland-Pfalz | 6.45 | 49.57 | 1 |
| 9535518 | *Streptopelia turtur* | Rheinland-Pfalz | 7.57 | 49.54 | 1 |
| 9588703 | *Streptopelia turtur* | Rheinland-Pfalz | 8.31 | 49.95 | 1 |
| 9612201 | *Streptopelia turtur* | Rheinland-Pfalz | 7.98 | 50.47 | 1 |
| 9623452 | *Streptopelia turtur* | Rheinland-Pfalz | 6.97 | 50.00 | 1 |
| 9683923 | *Streptopelia turtur* | Rheinland-Pfalz | 7.49 | 50.70 | 1 |
| 9686082 | *Streptopelia turtur* | Rheinland-Pfalz | 8.13 | 49.56 | 1 |
| 9715170 | *Streptopelia turtur* | Rheinland-Pfalz | 8.36 | 49.74 | 1 |
| 9723554 | *Streptopelia turtur* | Rheinland-Pfalz | 8.37 | 49.85 | 1 |

| **ID** | **species** | **Country** | **long** | **lat** | **PO** |
|---|---|---|---|---|---|
| 9743295 | *Streptopelia turtur* | Rheinland-Pfalz | 6.53 | 49.70 | 1 |
| 9771337 | *Streptopelia turtur* | Rheinland-Pfalz | 7.72 | 49.76 | 1 |
| 9786399 | *Streptopelia turtur* | Rheinland-Pfalz | 6.98 | 49.98 | 1 |
| 9788712 | *Streptopelia turtur* | Rheinland-Pfalz | 6.52 | 49.55 | 1 |
| 9809799 | *Streptopelia turtur* | Rheinland-Pfalz | 6.67 | 49.64 | 1 |
| 9810231 | *Streptopelia turtur* | Rheinland-Pfalz | 8.20 | 49.48 | 1 |
| 9814830 | *Streptopelia turtur* | Rheinland-Pfalz | 7.16 | 49.98 | 1 |
| 9814842 | *Streptopelia turtur* | Rheinland-Pfalz | 7.15 | 49.96 | 1 |
| 9816276 | *Streptopelia turtur* | Rheinland-Pfalz | 6.23 | 50.06 | 1 |
| 9825642 | *Streptopelia turtur* | Rheinland-Pfalz | 7.43 | 50.33 | 1 |
| 9832866 | *Streptopelia turtur* | Rheinland-Pfalz | 6.27 | 49.99 | 1 |
| 9856843 | *Streptopelia turtur* | Rheinland-Pfalz | 6.54 | 49.69 | 1 |
| 9870346 | *Streptopelia turtur* | Rheinland-Pfalz | 7.36 | 50.51 | 1 |
| 9879993 | *Streptopelia turtur* | Rheinland-Pfalz | 8.16 | 49.36 | 1 |
| 9880470 | *Streptopelia turtur* | Rheinland-Pfalz | 6.30 | 50.16 | 1 |
| 9882436 | *Streptopelia turtur* | Rheinland-Pfalz | 6.97 | 49.99 | 1 |
| 9900555 | *Streptopelia turtur* | Rheinland-Pfalz | 7.67 | 50.57 | 1 |
| 9908059 | *Streptopelia turtur* | Rheinland-Pfalz | 7.75 | 50.60 | 1 |

| **ID** | **species** | **Country** | **long** | **lat** | **PO** |
|---|---|---|---|---|---|
| 9929103 | *Streptopelia turtur* | Rheinland-Pfalz | 8.39 | 49.39 | 1 |
| 9932721 | *Streptopelia turtur* | Rheinland-Pfalz | 8.03 | 49.75 | 1 |
| 9948623 | *Streptopelia turtur* | Rheinland-Pfalz | 7.68 | 50.60 | 1 |
| 9957671 | *Streptopelia turtur* | Rheinland-Pfalz | 7.93 | 49.94 | 1 |
| 9979763 | *Streptopelia turtur* | Rheinland-Pfalz | 7.37 | 50.38 | 1 |
| 9995794 | *Streptopelia turtur* | Rheinland-Pfalz | 6.86 | 49.90 | 1 |
| 133352 | *Streptopelia turtur* | Saarland | 7.21 | 49.55 | 1 |
| 133414 | *Streptopelia turtur* | Saarland | 7.22 | 49.54 | 1 |
| 137932 | *Streptopelia turtur* | Saarland | 7.22 | 49.55 | 1 |
| 558794 | *Streptopelia turtur* | Saarland | 7.29 | 49.22 | 1 |
| 1707020 | *Streptopelia turtur* | Saarland | 6.64 | 49.35 | 1 |
| 1743998 | *Streptopelia turtur* | Saarland | 7.28 | 49.17 | 1 |
| 1780312 | *Streptopelia turtur* | Saarland | 7.01 | 49.57 | 1 |
| 1797806 | *Streptopelia turtur* | Saarland | 7.02 | 49.51 | 1 |
| 1810421 | *Streptopelia turtur* | Saarland | 6.75 | 49.28 | 1 |
| 1828266 | *Streptopelia turtur* | Saarland | 6.78 | 49.52 | 1 |
| 1829505 | *Streptopelia turtur* | Saarland | 6.95 | 49.48 | 1 |
| 1855398 | *Streptopelia turtur* | Saarland | 6.64 | 49.31 | 1 |

| **ID** | **species** | **Country** | **long** | **lat** | **PO** |
|---|---|---|---|---|---|
| 1862789 | *Streptopelia turtur* | Saarland | 6.73 | 49.25 | 1 |
| 1908203 | *Streptopelia turtur* | Saarland | 6.94 | 49.51 | 1 |
| 1910373 | *Streptopelia turtur* | Saarland | 6.71 | 49.42 | 1 |
| 2006416 | *Streptopelia turtur* | Saarland | 7.13 | 49.17 | 1 |
| 2010024 | *Streptopelia turtur* | Saarland | 7.21 | 49.51 | 1 |
| 2033349 | *Streptopelia turtur* | Saarland | 6.36 | 49.52 | 1 |
| 5302277 | *Streptopelia turtur* | Saarland | 7.20 | 49.16 | 1 |
| 5322027 | *Streptopelia turtur* | Saarland | 7.23 | 49.54 | 1 |
| 5345574 | *Streptopelia turtur* | Saarland | 6.36 | 49.53 | 1 |
| 5354520 | *Streptopelia turtur* | Saarland | 7.27 | 49.19 | 1 |
| 5368520 | *Streptopelia turtur* | Saarland | 7.31 | 49.18 | 1 |
| 5409866 | *Streptopelia turtur* | Saarland | 7.28 | 49.21 | 1 |
| 5422749 | *Streptopelia turtur* | Saarland | 7.21 | 49.16 | 1 |
| 5505411 | *Streptopelia turtur* | Saarland | 6.76 | 49.27 | 1 |
| 5524216 | *Streptopelia turtur* | Saarland | 7.22 | 49.18 | 1 |
| 5542780 | *Streptopelia turtur* | Saarland | 6.80 | 49.43 | 1 |
| 5560891 | *Streptopelia turtur* | Saarland | 7.29 | 49.19 | 1 |
| 5561514 | *Streptopelia turtur* | Saarland | 7.23 | 49.22 | 1 |

| **ID** | **species** | **Country** | **long** | **lat** | **PO** |
|---|---|---|---|---|---|
| 5564311 | *Streptopelia turtur* | Saarland | 6.70 | 49.40 | 1 |
| 5569700 | *Streptopelia turtur* | Saarland | 6.59 | 49.39 | 1 |
| 5647159 | *Streptopelia turtur* | Saarland | 6.70 | 49.49 | 1 |
| 5647217 | *Streptopelia turtur* | Saarland | 6.70 | 49.45 | 1 |
| 5647236 | *Streptopelia turtur* | Saarland | 6.70 | 49.44 | 1 |
| 5647260 | *Streptopelia turtur* | Saarland | 6.69 | 49.44 | 1 |
| 5660712 | *Streptopelia turtur* | Saarland | 6.75 | 49.39 | 1 |
| 5691939 | *Streptopelia turtur* | Saarland | 6.54 | 49.44 | 1 |
| 6706655 | *Streptopelia turtur* | Saarland | 6.55 | 49.40 | 1 |
| 6706683 | *Streptopelia turtur* | Saarland | 6.55 | 49.40 | 1 |
| 6819570 | *Streptopelia turtur* | Saarland | 6.69 | 49.48 | 1 |
| 6819743 | *Streptopelia turtur* | Saarland | 6.40 | 49.47 | 1 |
| 6819759 | *Streptopelia turtur* | Saarland | 6.55 | 49.40 | 1 |
| 6852152 | *Streptopelia turtur* | Saarland | 6.47 | 49.47 | 1 |
| 9487144 | *Streptopelia turtur* | Saarland | 6.75 | 49.42 | 1 |
| 9544998 | *Streptopelia turtur* | Saarland | 6.41 | 49.48 | 1 |
| 9545028 | *Streptopelia turtur* | Saarland | 6.41 | 49.50 | 1 |
| 9545038 | *Streptopelia turtur* | Saarland | 6.43 | 49.48 | 1 |

| **ID** | **species** | **Country** | **long** | **lat** | **PO** |
|---|---|---|---|---|---|
| 9637093 | *Streptopelia turtur* | Saarland | 7.20 | 49.53 | 1 |
| 9638971 | *Streptopelia turtur* | Saarland | 7.20 | 49.53 | 1 |
| 9845549 | *Streptopelia turtur* | Saarland | 6.84 | 49.58 | 1 |
| 9962318 | *Streptopelia turtur* | Saarland | 6.76 | 49.30 | 1 |
| 146475 | *Streptopelia turtur* | Sachsen | 12.91 | 51.02 | 1 |
| 146491 | *Streptopelia turtur* | Sachsen | 12.68 | 50.95 | 1 |
| 153822 | *Streptopelia turtur* | Sachsen | 13.52 | 50.65 | 1 |
| 153938 | *Streptopelia turtur* | Sachsen | 12.77 | 50.92 | 1 |
| 217177 | *Streptopelia turtur* | Sachsen | 12.22 | 51.16 | 1 |
| 217273 | *Streptopelia turtur* | Sachsen | 12.43 | 51.24 | 1 |
| 358785 | *Streptopelia turtur* | Sachsen | 14.26 | 51.01 | 1 |
| 359345 | *Streptopelia turtur* | Sachsen | 14.26 | 51.05 | 1 |
| 574966 | *Streptopelia turtur* | Sachsen | 14.45 | 51.54 | 1 |
| 574999 | *Streptopelia turtur* | Sachsen | 14.46 | 51.55 | 1 |
| 580344 | *Streptopelia turtur* | Sachsen | 14.96 | 51.22 | 1 |
| 640517 | *Streptopelia turtur* | Sachsen | 14.27 | 51.04 | 1 |
| 640546 | *Streptopelia turtur* | Sachsen | 14.27 | 51.03 | 1 |
| 640604 | *Streptopelia turtur* | Sachsen | 14.28 | 51.02 | 1 |

| **ID** | **species** | **Country** | **long** | **lat** | **PO** |
|---|---|---|---|---|---|
| 754503 | *Streptopelia turtur* | Sachsen | 14.54 | 51.29 | 1 |
| 1730868 | *Streptopelia turtur* | Sachsen | 12.76 | 50.68 | 1 |
| 1746195 | *Streptopelia turtur* | Sachsen | 12.71 | 50.69 | 1 |
| 1747516 | *Streptopelia turtur* | Sachsen | 14.92 | 51.23 | 1 |
| 1754365 | *Streptopelia turtur* | Sachsen | 12.76 | 50.88 | 1 |
| 1764525 | *Streptopelia turtur* | Sachsen | 14.63 | 51.32 | 1 |
| 1779633 | *Streptopelia turtur* | Sachsen | 15.02 | 51.28 | 1 |
| 1779670 | *Streptopelia turtur* | Sachsen | 15.03 | 51.28 | 1 |
| 1784648 | *Streptopelia turtur* | Sachsen | 14.87 | 51.17 | 1 |
| 1795249 | *Streptopelia turtur* | Sachsen | 12.55 | 50.78 | 1 |
| 1800309 | *Streptopelia turtur* | Sachsen | 14.93 | 51.09 | 1 |
| 1823768 | *Streptopelia turtur* | Sachsen | 14.85 | 51.41 | 1 |
| 1844405 | *Streptopelia turtur* | Sachsen | 14.90 | 51.27 | 1 |
| 1864509 | *Streptopelia turtur* | Sachsen | 13.94 | 50.82 | 1 |
| 1884791 | *Streptopelia turtur* | Sachsen | 14.75 | 50.96 | 1 |
| 1925112 | *Streptopelia turtur* | Sachsen | 14.20 | 50.99 | 1 |
| 1925219 | *Streptopelia turtur* | Sachsen | 14.10 | 51.07 | 1 |
| 1952839 | *Streptopelia turtur* | Sachsen | 14.24 | 51.05 | 1 |

| **ID** | **species** | **Country** | **long** | **lat** | **PO** |
|---|---|---|---|---|---|
| 1962862 | *Streptopelia turtur* | Sachsen | 14.99 | 51.32 | 1 |
| 1962863 | *Streptopelia turtur* | Sachsen | 14.98 | 51.33 | 1 |
| 1973685 | *Streptopelia turtur* | Sachsen | 14.96 | 51.23 | 1 |
| 1981178 | *Streptopelia turtur* | Sachsen | 12.82 | 50.97 | 1 |
| 1985024 | *Streptopelia turtur* | Sachsen | 12.90 | 50.70 | 1 |
| 2002694 | *Streptopelia turtur* | Sachsen | 12.40 | 51.55 | 1 |
| 2018641 | *Streptopelia turtur* | Sachsen | 14.77 | 50.95 | 1 |
| 2020852 | *Streptopelia turtur* | Sachsen | 14.87 | 50.99 | 1 |
| 2055513 | *Streptopelia turtur* | Sachsen | 14.91 | 51.40 | 1 |
| 3046123 | *Streptopelia turtur* | Sachsen | 12.80 | 50.95 | 1 |
| 3087543 | *Streptopelia turtur* | Sachsen | 14.68 | 50.86 | 1 |
| 3087560 | *Streptopelia turtur* | Sachsen | 14.68 | 50.87 | 1 |
| 3905194 | *Streptopelia turtur* | Sachsen | 13.56 | 50.96 | 1 |
| 4522022 | *Streptopelia turtur* | Sachsen | 13.80 | 50.95 | 1 |
| 5305795 | *Streptopelia turtur* | Sachsen | 14.12 | 51.33 | 1 |
| 5323121 | *Streptopelia turtur* | Sachsen | 12.71 | 51.20 | 1 |
| 5367189 | *Streptopelia turtur* | Sachsen | 14.91 | 51.08 | 1 |
| 5367212 | *Streptopelia turtur* | Sachsen | 14.89 | 51.08 | 1 |

| **ID** | **species** | **Country** | **long** | **lat** | **PO** |
|---|---|---|---|---|---|
| 5376992 | *Streptopelia turtur* | Sachsen | 14.91 | 51.27 | 1 |
| 5395360 | *Streptopelia turtur* | Sachsen | 14.06 | 51.21 | 1 |
| 5401957 | *Streptopelia turtur* | Sachsen | 12.87 | 50.94 | 1 |
| 5402743 | *Streptopelia turtur* | Sachsen | 14.41 | 51.06 | 1 |
| 5413944 | *Streptopelia turtur* | Sachsen | 14.91 | 51.08 | 1 |
| 5424840 | *Streptopelia turtur* | Sachsen | 14.76 | 50.88 | 1 |
| 5425710 | *Streptopelia turtur* | Sachsen | 14.59 | 51.30 | 1 |
| 5455854 | *Streptopelia turtur* | Sachsen | 12.71 | 51.20 | 1 |
| 5476866 | *Streptopelia turtur* | Sachsen | 13.36 | 50.72 | 1 |
| 5547025 | *Streptopelia turtur* | Sachsen | 14.88 | 51.39 | 1 |
| 5561698 | *Streptopelia turtur* | Sachsen | 12.71 | 51.20 | 1 |
| 5565641 | *Streptopelia turtur* | Sachsen | 14.84 | 51.40 | 1 |
| 5571265 | *Streptopelia turtur* | Sachsen | 13.21 | 51.39 | 1 |
| 5615268 | *Streptopelia turtur* | Sachsen | 14.97 | 51.11 | 1 |
| 5643020 | *Streptopelia turtur* | Sachsen | 14.94 | 51.29 | 1 |
| 5656451 | *Streptopelia turtur* | Sachsen | 14.69 | 50.93 | 1 |
| 5659908 | *Streptopelia turtur* | Sachsen | 14.12 | 51.25 | 1 |
| 5729880 | *Streptopelia turtur* | Sachsen | 13.13 | 51.55 | 1 |

| **ID** | **species** | **Country** | **long** | **lat** | **PO** |
|---|---|---|---|---|---|
| 6336313 | *Streptopelia turtur* | Sachsen | 14.37 | 51.47 | 1 |
| 7225595 | *Streptopelia turtur* | Sachsen | 13.90 | 50.98 | 1 |
| 7503305 | *Streptopelia turtur* | Sachsen | 13.48 | 50.99 | 1 |
| 7508884 | *Streptopelia turtur* | Sachsen | 13.48 | 50.98 | 1 |
| 7591503 | *Streptopelia turtur* | Sachsen | 14.87 | 51.39 | 1 |
| 7620162 | *Streptopelia turtur* | Sachsen | 14.89 | 51.36 | 1 |
| 9488063 | *Streptopelia turtur* | Sachsen | 14.94 | 51.23 | 1 |
| 9543320 | *Streptopelia turtur* | Sachsen | 12.44 | 51.12 | 1 |
| 9545523 | *Streptopelia turtur* | Sachsen | 12.66 | 50.87 | 1 |
| 9553873 | *Streptopelia turtur* | Sachsen | 11.95 | 50.59 | 1 |
| 9614236 | *Streptopelia turtur* | Sachsen | 14.14 | 51.51 | 1 |
| 9615803 | *Streptopelia turtur* | Sachsen | 12.85 | 50.66 | 1 |
| 9653477 | *Streptopelia turtur* | Sachsen | 14.97 | 51.21 | 1 |
| 9653521 | *Streptopelia turtur* | Sachsen | 14.98 | 51.27 | 1 |
| 9673611 | *Streptopelia turtur* | Sachsen | 14.88 | 51.22 | 1 |
| 9747076 | *Streptopelia turtur* | Sachsen | 12.75 | 50.72 | 1 |
| 9753826 | *Streptopelia turtur* | Sachsen | 14.08 | 51.23 | 1 |
| 9805495 | *Streptopelia turtur* | Sachsen | 11.95 | 50.59 | 1 |

| **ID** | **species** | **Country** | **long** | **lat** | **PO** |
|---|---|---|---|---|---|
| 9813906 | *Streptopelia turtur* | Sachsen | 12.71 | 50.67 | 1 |
| 9838420 | *Streptopelia turtur* | Sachsen | 14.08 | 51.21 | 1 |
| 9856675 | *Streptopelia turtur* | Sachsen | 12.35 | 51.16 | 1 |
| 9884461 | *Streptopelia turtur* | Sachsen | 13.11 | 51.16 | 1 |
| 9891799 | *Streptopelia turtur* | Sachsen | 12.49 | 51.08 | 1 |
| 9908956 | *Streptopelia turtur* | Sachsen | 11.99 | 50.59 | 1 |
| 9908968 | *Streptopelia turtur* | Sachsen | 11.97 | 50.60 | 1 |
| 9964094 | *Streptopelia turtur* | Sachsen | 11.99 | 50.60 | 1 |
| 9969726 | *Streptopelia turtur* | Sachsen | 13.73 | 50.74 | 1 |
| 9973646 | *Streptopelia turtur* | Sachsen | 13.53 | 50.68 | 1 |
| 10011456 | *Streptopelia turtur* | Sachsen | 14.98 | 51.23 | 1 |
| 10045423 | *Streptopelia turtur* | Sachsen | 14.82 | 51.01 | 1 |
| 10060165 | *Streptopelia turtur* | Sachsen | 11.96 | 50.40 | 1 |
| 10081478 | *Streptopelia turtur* | Sachsen | 12.98 | 50.73 | 1 |
| 10434920 | *Streptopelia turtur* | Sachsen | 14.02 | 50.86 | 1 |
| 10434925 | *Streptopelia turtur* | Sachsen | 14.02 | 50.82 | 1 |
| 10568187 | *Streptopelia turtur* | Sachsen | 12.82 | 50.65 | 1 |
| 10627878 | *Streptopelia turtur* | Sachsen | 12.80 | 50.66 | 1 |

| **ID** | **species** | **Country** | **long** | **lat** | **PO** |
|---|---|---|---|---|---|
| 1701709 | *Streptopelia turtur* | Sachsen-Anhalt | 11.17 | 52.69 | 1 |
| 1721240 | *Streptopelia turtur* | Sachsen-Anhalt | 11.41 | 52.64 | 1 |
| 1721844 | *Streptopelia turtur* | Sachsen-Anhalt | 11.39 | 51.80 | 1 |
| 1733420 | *Streptopelia turtur* | Sachsen-Anhalt | 11.60 | 51.44 | 1 |
| 1754040 | *Streptopelia turtur* | Sachsen-Anhalt | 11.68 | 51.78 | 1 |
| 1780313 | *Streptopelia turtur* | Sachsen-Anhalt | 11.18 | 52.09 | 1 |
| 1780337 | *Streptopelia turtur* | Sachsen-Anhalt | 11.24 | 51.97 | 1 |
| 1782066 | *Streptopelia turtur* | Sachsen-Anhalt | 11.33 | 52.61 | 1 |
| 1822211 | *Streptopelia turtur* | Sachsen-Anhalt | 11.84 | 51.32 | 1 |
| 1822267 | *Streptopelia turtur* | Sachsen-Anhalt | 11.82 | 51.32 | 1 |
| 1828861 | *Streptopelia turtur* | Sachsen-Anhalt | 13.02 | 51.66 | 1 |
| 1832599 | *Streptopelia turtur* | Sachsen-Anhalt | 11.14 | 52.69 | 1 |
| 1857678 | *Streptopelia turtur* | Sachsen-Anhalt | 11.60 | 52.57 | 1 |
| 1865681 | *Streptopelia turtur* | Sachsen-Anhalt | 11.48 | 52.64 | 1 |
| 1906183 | *Streptopelia turtur* | Sachsen-Anhalt | 10.69 | 51.92 | 1 |
| 1908407 | *Streptopelia turtur* | Sachsen-Anhalt | 11.64 | 52.53 | 1 |
| 1919733 | *Streptopelia turtur* | Sachsen-Anhalt | 11.92 | 52.75 | 1 |
| 1919812 | *Streptopelia turtur* | Sachsen-Anhalt | 11.14 | 52.69 | 1 |

| ID | species | Country | long | lat | PO |
|---|---|---|---|---|---|
| 1931180 | *Streptopelia turtur* | Sachsen-Anhalt | 11.98 | 51.31 | 1 |
| 1935337 | *Streptopelia turtur* | Sachsen-Anhalt | 11.65 | 52.34 | 1 |
| 1989511 | *Streptopelia turtur* | Sachsen-Anhalt | 11.64 | 52.57 | 1 |
| 2002784 | *Streptopelia turtur* | Sachsen-Anhalt | 11.71 | 52.24 | 1 |
| 2027528 | *Streptopelia turtur* | Sachsen-Anhalt | 11.43 | 51.79 | 1 |
| 2060853 | *Streptopelia turtur* | Sachsen-Anhalt | 11.73 | 51.68 | 1 |
| 3811088 | *Streptopelia turtur* | Sachsen-Anhalt | 11.66 | 52.04 | 1 |
| 5271163 | *Streptopelia turtur* | Sachsen-Anhalt | 12.37 | 51.91 | 1 |
| 5339600 | *Streptopelia turtur* | Sachsen-Anhalt | 12.10 | 51.36 | 1 |
| 5342482 | *Streptopelia turtur* | Sachsen-Anhalt | 11.33 | 52.72 | 1 |
| 5379062 | *Streptopelia turtur* | Sachsen-Anhalt | 11.28 | 52.59 | 1 |
| 5381162 | *Streptopelia turtur* | Sachsen-Anhalt | 11.47 | 51.39 | 1 |
| 5404171 | *Streptopelia turtur* | Sachsen-Anhalt | 12.12 | 51.12 | 1 |
| 5404999 | *Streptopelia turtur* | Sachsen-Anhalt | 12.12 | 51.14 | 1 |
| 5415852 | *Streptopelia turtur* | Sachsen-Anhalt | 12.17 | 51.16 | 1 |
| 5416086 | *Streptopelia turtur* | Sachsen-Anhalt | 12.16 | 51.17 | 1 |
| 5418944 | *Streptopelia turtur* | Sachsen-Anhalt | 11.73 | 51.21 | 1 |
| 5434225 | *Streptopelia turtur* | Sachsen-Anhalt | 12.16 | 51.13 | 1 |

| **ID** | **species** | **Country** | **long** | **lat** | **PO** |
|---|---|---|---|---|---|
| 5446501 | *Streptopelia turtur* | Sachsen-Anhalt | 11.77 | 52.55 | 1 |
| 5506900 | *Streptopelia turtur* | Sachsen-Anhalt | 11.92 | 52.60 | 1 |
| 5562795 | *Streptopelia turtur* | Sachsen-Anhalt | 11.69 | 51.69 | 1 |
| 5570276 | *Streptopelia turtur* | Sachsen-Anhalt | 11.78 | 51.49 | 1 |
| 5578892 | *Streptopelia turtur* | Sachsen-Anhalt | 11.90 | 51.32 | 1 |
| 5581637 | *Streptopelia turtur* | Sachsen-Anhalt | 11.21 | 52.03 | 1 |
| 5633050 | *Streptopelia turtur* | Sachsen-Anhalt | 11.94 | 52.59 | 1 |
| 5636070 | *Streptopelia turtur* | Sachsen-Anhalt | 11.88 | 51.63 | 1 |
| 5656431 | *Streptopelia turtur* | Sachsen-Anhalt | 11.98 | 51.31 | 1 |
| 5689892 | *Streptopelia turtur* | Sachsen-Anhalt | 11.73 | 52.17 | 1 |
| 5704993 | *Streptopelia turtur* | Sachsen-Anhalt | 11.78 | 51.35 | 1 |
| 5727735 | *Streptopelia turtur* | Sachsen-Anhalt | 11.74 | 52.19 | 1 |
| 9568309 | *Streptopelia turtur* | Sachsen-Anhalt | 11.28 | 51.93 | 1 |
| 9568374 | *Streptopelia turtur* | Sachsen-Anhalt | 11.85 | 51.57 | 1 |
| 9593886 | *Streptopelia turtur* | Sachsen-Anhalt | 10.83 | 52.70 | 1 |
| 9665963 | *Streptopelia turtur* | Sachsen-Anhalt | 11.39 | 51.80 | 1 |
| 9665977 | *Streptopelia turtur* | Sachsen-Anhalt | 11.38 | 51.81 | 1 |
| 9667002 | *Streptopelia turtur* | Sachsen-Anhalt | 11.87 | 51.30 | 1 |

| **ID** | **species** | **Country** | **long** | **lat** | **PO** |
|---|---|---|---|---|---|
| 9667356 | *Streptopelia turtur* | Sachsen-Anhalt | 11.76 | 51.49 | 1 |
| 9690309 | *Streptopelia turtur* | Sachsen-Anhalt | 11.28 | 52.59 | 1 |
| 9729419 | *Streptopelia turtur* | Sachsen-Anhalt | 11.92 | 51.53 | 1 |
| 9740107 | *Streptopelia turtur* | Sachsen-Anhalt | 11.73 | 52.44 | 1 |
| 9751607 | *Streptopelia turtur* | Sachsen-Anhalt | 11.76 | 51.69 | 1 |
| 9776781 | *Streptopelia turtur* | Sachsen-Anhalt | 11.92 | 51.54 | 1 |
| 9794647 | *Streptopelia turtur* | Sachsen-Anhalt | 12.07 | 51.37 | 1 |
| 9863408 | *Streptopelia turtur* | Sachsen-Anhalt | 11.85 | 51.30 | 1 |
| 9867659 | *Streptopelia turtur* | Sachsen-Anhalt | 11.57 | 51.90 | 1 |
| 9892969 | *Streptopelia turtur* | Sachsen-Anhalt | 11.90 | 51.53 | 1 |
| 9894067 | *Streptopelia turtur* | Sachsen-Anhalt | 12.09 | 51.77 | 1 |
| 9907059 | *Streptopelia turtur* | Sachsen-Anhalt | 11.95 | 52.60 | 1 |
| 9934972 | *Streptopelia turtur* | Sachsen-Anhalt | 12.04 | 51.38 | 1 |
| 9937789 | *Streptopelia turtur* | Sachsen-Anhalt | 11.84 | 51.35 | 1 |
| 9985709 | *Streptopelia turtur* | Sachsen-Anhalt | 11.81 | 51.31 | 1 |
| 9985725 | *Streptopelia turtur* | Sachsen-Anhalt | 11.85 | 51.31 | 1 |
| 10001152 | *Streptopelia turtur* | Sachsen-Anhalt | 11.80 | 51.48 | 1 |
| 10004260 | *Streptopelia turtur* | Sachsen-Anhalt | 12.16 | 51.13 | 1 |

| ID | species | Country | long | lat | PO |
|---|---|---|---|---|---|
| 10013289 | *Streptopelia turtur* | Sachsen-Anhalt | 11.95 | 52.50 | 1 |
| 10029862 | *Streptopelia turtur* | Sachsen-Anhalt | 11.88 | 51.33 | 1 |
| 10032878 | *Streptopelia turtur* | Sachsen-Anhalt | 11.89 | 51.54 | 1 |
| 10043422 | *Streptopelia turtur* | Sachsen-Anhalt | 11.95 | 52.24 | 1 |
| 10049588 | *Streptopelia turtur* | Sachsen-Anhalt | 12.39 | 51.66 | 1 |
| 10066102 | *Streptopelia turtur* | Sachsen-Anhalt | 11.74 | 52.20 | 1 |
| 10069007 | *Streptopelia turtur* | Sachsen-Anhalt | 11.82 | 51.31 | 1 |
| 10073466 | *Streptopelia turtur* | Sachsen-Anhalt | 11.70 | 51.66 | 1 |
| 10082243 | *Streptopelia turtur* | Sachsen-Anhalt | 11.70 | 51.48 | 1 |
| 1591462 | *Streptopelia turtur* | Thüringen | 10.87 | 51.15 | 1 |
| 1639118 | *Streptopelia turtur* | Thüringen | 10.88 | 51.14 | 1 |
| 1703180 | *Streptopelia turtur* | Thüringen | 10.59 | 51.09 | 1 |
| 1703567 | *Streptopelia turtur* | Thüringen | 10.65 | 51.05 | 1 |
| 1721997 | *Streptopelia turtur* | Thüringen | 10.20 | 50.85 | 1 |
| 1746093 | *Streptopelia turtur* | Thüringen | 10.57 | 50.39 | 1 |
| 1770313 | *Streptopelia turtur* | Thüringen | 10.44 | 51.31 | 1 |
| 1771391 | *Streptopelia turtur* | Thüringen | 11.21 | 51.32 | 1 |
| 1808876 | *Streptopelia turtur* | Thüringen | 12.04 | 50.76 | 1 |

| ID | species | Country | long | lat | PO |
|---|---|---|---|---|---|
| 1808882 | *Streptopelia turtur* | Thüringen | 11.97 | 50.76 | 1 |
| 1820780 | *Streptopelia turtur* | Thüringen | 10.91 | 51.19 | 1 |
| 1828055 | *Streptopelia turtur* | Thüringen | 11.28 | 51.32 | 1 |
| 1828057 | *Streptopelia turtur* | Thüringen | 11.26 | 51.31 | 1 |
| 1829237 | *Streptopelia turtur* | Thüringen | 10.35 | 50.80 | 1 |
| 1853838 | *Streptopelia turtur* | Thüringen | 11.21 | 51.34 | 1 |
| 1855755 | *Streptopelia turtur* | Thüringen | 11.65 | 50.81 | 1 |
| 1857347 | *Streptopelia turtur* | Thüringen | 10.50 | 51.05 | 1 |
| 1857697 | *Streptopelia turtur* | Thüringen | 10.65 | 50.47 | 1 |
| 1871821 | *Streptopelia turtur* | Thüringen | 11.98 | 50.78 | 1 |
| 1878027 | *Streptopelia turtur* | Thüringen | 11.16 | 51.35 | 1 |
| 1881462 | *Streptopelia turtur* | Thüringen | 10.81 | 50.43 | 1 |
| 1890374 | *Streptopelia turtur* | Thüringen | 10.53 | 51.10 | 1 |
| 1900168 | *Streptopelia turtur* | Thüringen | 10.73 | 51.07 | 1 |
| 1907137 | *Streptopelia turtur* | Thüringen | 10.20 | 50.83 | 1 |
| 1926551 | *Streptopelia turtur* | Thüringen | 10.69 | 51.17 | 1 |
| 1946967 | *Streptopelia turtur* | Thüringen | 10.79 | 51.22 | 1 |
| 2025153 | *Streptopelia turtur* | Thüringen | 10.73 | 50.50 | 1 |

| ID | species | Country | long | lat | PO |
|---|---|---|---|---|---|
| 2034985 | *Streptopelia turtur* | Thüringen | 12.00 | 50.80 | 1 |
| 2035900 | *Streptopelia turtur* | Thüringen | 11.15 | 51.35 | 1 |
| 2055659 | *Streptopelia turtur* | Thüringen | 10.61 | 50.37 | 1 |
| 2058186 | *Streptopelia turtur* | Thüringen | 10.49 | 51.10 | 1 |
| 2061174 | *Streptopelia turtur* | Thüringen | 11.16 | 50.94 | 1 |
| 2061251 | *Streptopelia turtur* | Thüringen | 11.25 | 50.87 | 1 |
| 2078564 | *Streptopelia turtur* | Thüringen | 10.45 | 51.03 | 1 |
| 2086131 | *Streptopelia turtur* | Thüringen | 10.87 | 51.13 | 1 |
| 2088184 | *Streptopelia turtur* | Thüringen | 10.59 | 50.54 | 1 |
| 5334718 | *Streptopelia turtur* | Thüringen | 9.97 | 51.35 | 1 |
| 5336763 | *Streptopelia turtur* | Thüringen | 10.31 | 51.23 | 1 |
| 5340159 | *Streptopelia turtur* | Thüringen | 10.57 | 50.54 | 1 |
| 5368236 | *Streptopelia turtur* | Thüringen | 10.64 | 50.37 | 1 |
| 5368339 | *Streptopelia turtur* | Thüringen | 10.60 | 50.36 | 1 |
| 5371780 | *Streptopelia turtur* | Thüringen | 10.76 | 51.20 | 1 |
| 5439092 | *Streptopelia turtur* | Thüringen | 10.43 | 51.40 | 1 |
| 5453146 | *Streptopelia turtur* | Thüringen | 10.36 | 50.81 | 1 |
| 5463815 | *Streptopelia turtur* | Thüringen | 10.48 | 50.38 | 1 |

| **ID** | **species** | **Country** | **long** | **lat** | **PO** |
|---|---|---|---|---|---|
| 5499202 | *Streptopelia turtur* | Thüringen | 10.86 | 50.40 | 1 |
| 5515058 | *Streptopelia turtur* | Thüringen | 10.33 | 51.39 | 1 |
| 5547059 | *Streptopelia turtur* | Thüringen | 10.68 | 50.44 | 1 |
| 5547062 | *Streptopelia turtur* | Thüringen | 10.66 | 50.43 | 1 |
| 5548251 | *Streptopelia turtur* | Thüringen | 10.48 | 50.39 | 1 |
| 5549917 | *Streptopelia turtur* | Thüringen | 10.89 | 50.81 | 1 |
| 5550991 | *Streptopelia turtur* | Thüringen | 10.46 | 50.43 | 1 |
| 5583990 | *Streptopelia turtur* | Thüringen | 11.13 | 51.20 | 1 |
| 5583996 | *Streptopelia turtur* | Thüringen | 11.26 | 51.13 | 1 |
| 5592995 | *Streptopelia turtur* | Thüringen | 10.56 | 50.43 | 1 |
| 5605941 | *Streptopelia turtur* | Thüringen | 11.82 | 50.68 | 1 |
| 5623639 | *Streptopelia turtur* | Thüringen | 10.87 | 50.41 | 1 |
| 5650956 | *Streptopelia turtur* | Thüringen | 10.15 | 51.38 | 1 |
| 5653674 | *Streptopelia turtur* | Thüringen | 11.51 | 50.47 | 1 |
| 5654874 | *Streptopelia turtur* | Thüringen | 11.93 | 50.92 | 1 |
| 5692766 | *Streptopelia turtur* | Thüringen | 10.91 | 50.87 | 1 |
| 5709389 | *Streptopelia turtur* | Thüringen | 10.61 | 50.87 | 1 |
| 5718373 | *Streptopelia turtur* | Thüringen | 10.00 | 51.36 | 1 |

| **ID** | **species** | **Country** | **long** | **lat** | **PO** |
|---|---|---|---|---|---|
| 7054394 | *Streptopelia turtur* | Thüringen | 10.44 | 51.01 | 1 |
| 9483203 | *Streptopelia turtur* | Thüringen | 9.97 | 51.37 | 1 |
| 9493981 | *Streptopelia turtur* | Thüringen | 10.89 | 51.18 | 1 |
| 9584692 | *Streptopelia turtur* | Thüringen | 11.46 | 51.12 | 1 |
| 9659891 | *Streptopelia turtur* | Thüringen | 11.98 | 50.81 | 1 |
| 9680500 | *Streptopelia turtur* | Thüringen | 10.43 | 51.11 | 1 |
| 9682035 | *Streptopelia turtur* | Thüringen | 9.95 | 51.37 | 1 |
| 9794386 | *Streptopelia turtur* | Thüringen | 12.04 | 50.97 | 1 |
| 9822672 | *Streptopelia turtur* | Thüringen | 10.67 | 50.41 | 1 |
| 9822724 | *Streptopelia turtur* | Thüringen | 10.76 | 50.25 | 1 |
| 9859321 | *Streptopelia turtur* | Thüringen | 10.02 | 51.36 | 1 |
| 9900084 | *Streptopelia turtur* | Thüringen | 10.65 | 50.48 | 1 |
| 9980843 | *Streptopelia turtur* | Thüringen | 11.15 | 51.33 | 1 |
| 9990994 | *Streptopelia turtur* | Thüringen | 10.23 | 51.04 | 1 |
| 9994025 | *Streptopelia turtur* | Thüringen | 11.08 | 50.89 | 1 |
| 10095326 | *Streptopelia turtur* | Thüringen | 11.88 | 50.46 | 1 |
| 1751869 | *Streptopelia turtur* | Thüringen | 10.21 | 51.13 | 1 |
| 1851777 | *Streptopelia turtur* | Schleswig-Holstein | 10.21 | 54.02 | 1 |

| **ID** | **species** | **Country** | **long** | **lat** | **PO** |
|---|---|---|---|---|---|
| 1957241 | *Streptopelia turtur* | Schleswig-Holstein | 10.75 | 53.51 | 1 |
| 5436041 | *Streptopelia turtur* | Schleswig-Holstein | 10.76 | 53.50 | 1 |
| 9543969 | *Streptopelia turtur* | Schleswig-Holstein | 9.17 | 54.83 | 1 |
| 9878789 | *Streptopelia turtur* | Schleswig-Holstein | 9.52 | 54.40 | 1 |
